# AOO-ANFIS: A Novel Bio-Inspired Neuro-Fuzzy Controller for Hydrostatic Transmission Stroke Control

**DOI:** 10.3390/biomimetics11100732

**Published:** 2026-10-13

**Authors:** Hakan Ülker

**Affiliations:** Department of Mechanical Engineering, Bursa Technical University, Bursa 16310, Türkiye; hakan.ulker@btu.edu.tr

**Keywords:** animated oat optimization, ANFIS, AOO-ANFIS, bio-inspired neuro-fuzzy controller, hydrostatic transmission, stroke control, reference tracking, disturbance rejection

## Abstract

Hydrostatic transmissions (HSTs) rely on coordinated pump and motor displacement control to achieve smooth stroke regulation. This study proposes AOO-ANFIS, a bio-inspired neuro-fuzzy controller in which the 66 premise and consequent parameters of a two-input, two-output first-order Takagi–Sugeno adaptive neuro-fuzzy inference system (ANFIS) are tuned by the animated oat optimization (AOO) algorithm rather than by ANFIS’s conventional gradient-based hybrid learning rule. The controller is trained as a data-driven surrogate for a previously validated multiple model predictive controller (MMPC): closed-loop input–output samples generated by the MMPC are used as supervised targets, enabling the resulting fuzzy controller to reproduce the MMPC control policy without solving an online optimization problem. On this identical tuning task, AOO was benchmarked against Harris Hawks Optimization (HHO), particle swarm optimization (PSO), a real-coded genetic algorithm (GA) and the covariance matrix adaptation evolution strategy (CMA-ES). It reached the lowest mean validation error, significantly lower than HHO, GA and CMA-ES and statistically indistinguishable from PSO. The AOO-ANFIS controller was subsequently deployed in a processor-in-the-loop simulation on a BeagleBone Black target, replacing the previously reported MMPC under identical deterministic and stochastic torque disturbance profiles. Relative to the MMPC, the AOO-ANFIS controller reduced the maximum overshoot from 25% to 14% under deterministic disturbances and from 24% to 14% under stochastic disturbances, reduced the closed-loop tracking RMSE by 7.8% and 30.1%, respectively, and reduced the integral squared-error indices ISE and ITSE by 15% and 21% under deterministic and by 51% and 41% under stochastic disturbances. It did so with an average control effort about 10 percentage lower and an average computational load 76–84% lower, at the cost of a longer peak time under stochastic disturbances: 1.8 s instead of 1 s. In an extended 300 s scenario containing reference levels, ramp rates, a sinusoidal segment and trapezoidal disturbances absent from the training data, the AOO-ANFIS maintained bounded tracking with an RMSE of 0.035 over the unseen segment, lower than that of a previously published ANN surrogate trained on the same data, 0.056. Both surrogates remained stable when the plant’s volumetric-efficiency maps were degraded by about 15%, with the AOO-ANFIS showing lower error. Moreover, this is the first application of the AOO algorithm to ANFIS tuning and the first bio-inspired-optimization-tuned ANFIS controller for HST stroke control.

## 1. Introduction

Hydrostatic transmission (HST) technology is extensively deployed across a broad spectrum of engineering applications due to its high-power density, flexible structural layout and continuously variable transmission capabilities [1,2]. The applications most closely related to the present work are the propulsion and working-function drives of heavy off-road vehicles, where the HST must track a stroke command. At the same time, the engine and load torques vary abruptly: construction machinery such as wheel loaders [3,4], agricultural tractors and self-propelled vehicles [5,6,7] and forestry forwarders subjected to transient load fluctuations and terrain bumps [8]. The same displacement-control problem arises, with different bandwidth requirements, in HST-based renewable-energy drivetrains such as low-head hydropower units [9] and wind turbines [10]. In all of these cases the pump and motor displacements must be regulated jointly, in real time, on embedded hardware of modest computational capacity, which is the setting addressed here.

Control of HST stroke and speed has evolved from classical feedback regulation toward robust, intelligent and predictive architectures that address the system’s nonlinearities, fluid compressibility and parameter uncertainty. Robust designs include $H_{\infty }$ speed regulation under variable torsional loads [11], $H_{\infty }$ velocity tracking of displacement-controlled actuators [12], mixed-sensitivity $H_{\infty }$ velocity control of variable-speed hydrostatic transmissions with robustness analysis under parametric uncertainty [13] and active disturbance rejection control of power-split units [14]. Model-free and learning-based designs include neural-network feedforward compensation of motor speed [15], adaptive fuzzy sliding mode control (SMC) of secondary-controlled HSTs [16,17], radial basis function sliding mode displacement matching [3] and multivariable fuzzy SMC of dual-motor HSTs [4]. For the specific problem considered in this paper, which is simultaneous primary (pump) and secondary (motor) displacement regulation of the Carlsson HST model [8], the author previously established a multiple model predictive controller (MMPC) [18] and showed that an artificial neural network (ANN) trained on MMPC closed-loop data can replicate its behavior at a fraction of the online computational cost [19]. Three limitations of that ANN surrogate motivate the present work: its internal representation is not interpretable, its hidden-layer size and training procedure must be selected by trial and error, and it was trained with gradient-based back-propagation, which is sensitive to initialization. A fuzzy-rule-based surrogate whose parameters are tuned by a global, population-based optimizer addresses all three.

The adaptive-network-based fuzzy inference system (ANFIS) [20] combines the learning capability of a feed-forward network with the transparent rule structure of a Takagi–Sugeno fuzzy model. ANFIS controllers have been applied to power converters and microgrids [21,22,23], electrical machine drives [24,25,26,27,28] and hydraulic and electro-hydraulic actuators such as the following: Essa et al. [29] achieved nearly overshoot-free cylinder position and force control with an ANFIS identified from ARX data; Yu et al. [30] mapped force error directly to valve current with an ANFIS force controller; Ursu et al. [31] used a neuro-fuzzy scheme for pump-controlled cylinder displacement; and Han et al. [32] tuned the gains of a PID controller with an ANFIS for a four-degree-of-freedom hydraulic manipulator. None of these applied ANFIS to the coupled pump-and-motor displacement problem of a hydrostatic transmission, and all of them trained the ANFIS with Jang’s gradient-based hybrid learning rule.

The animated oat optimization (AOO) algorithm was initially introduced as a nature-inspired metaheuristic simulating the seed dispersal, hygroscopic awn rolling and obstacle ejection dynamics of *Avena sterilis* L. [33], followed by critical methodological audits that re-evaluated its mathematical formulation and established simplified diagnostic baselines [34]. To resolve premature convergence and enhance population diversity, structural modifications such as sinusoidal chaotic mapping, adaptive disturbance factors and t-distribution mutations have been introduced for constrained engineering design problems [35]. In parallel with algorithmic advancements, AOO has gained substantial traction in optimal controller tuning, successfully determining the nonlinear parameters of an algebraic saturation-based PID controller for automatic voltage regulator systems to achieve ultra-fast, overshoot-free transient responses [36], optimizing all six parameters of a two-degree-of-freedom PID controller for high-precision DC motor speed regulation validated through real-time hardware-in-the-loop experimentation [37], optimizing a cascaded simplified fuzzy logic and fractional-order PID control strategy for distribution static compensators in electric vehicle-integrated microgrids to achieve rapid voltage regulation and superior power quality enhancement [38] and tuning a multi-objective frequency-decoupled fractional-order PID (FOPID) and SMC scheme for joint wind power and flywheel energy storage regulation [39]. Furthermore, the algorithm’s global exploration and fine local exploitation capabilities have proven highly effective in modern power electronic systems, including the optimization of five-parameter FOPID controllers for capacitor voltage balancing and total harmonic distortion reduction in packed u-cell multilevel inverters [40], the formulation of a hybrid AOO featuring adaptive contraction and reseeding for maximum power point tracking duty-cycle regulation in PMSG-based wind turbines equipped with SEPIC converters [41] and the development of an AOO-optimized PI control scheme for precise voltage regulation and dynamic power sharing in multi-port TriPort X-Gain Boost Converters integrating photovoltaic and battery sources [42].

The Harris Hawks optimization (HHO) algorithm was originally introduced as a nature-inspired, population-based metaheuristic that mathematically models the cooperative foraging behaviors and surprise pounce tactics of Harris’ hawks to solve diverse continuous and constrained real-world engineering design optimization problems [43]. Furthermore, HHO has proven highly effective in control systems engineering as an intelligent parameter tuning mechanism; specifically, it has been deployed to optimize the membership function coefficients of type-I and type-II fuzzy logic controllers in quarter-car active suspension systems, thereby effectively suppressing road-induced vehicle vibrations and substantially enhancing dynamic ride comfort [44].

Replacing the gradient-based update of the ANFIS premise parameters with a population-based metaheuristic is a well-established idea [45,46,47,48,49,50]; hybrid ANFIS models tuned by genetic algorithms, particle swarm optimization, ant colony optimization, the marine predators’ algorithm and HHO have been reported for forecasting, identification and control tasks [49,51,52,53,54,55,56,57,58,59,60,61,62,63,64,65]. The contribution of the present work is therefore not the general concept of metaheuristic ANFIS tuning. It lies in the specific pairing of ANFIS with the AOO algorithm [33], a plant-inspired metaheuristic introduced in 2025 whose reported benchmark performance on the CEC-2022 suite and on constrained engineering design problems has not yet been examined on a fuzzy-controller design problem, and the application of the resulting controller to HST stroke control, a problem previously solved only with model-based predictive control [18] or an ANN surrogate [19].

Because no optimizer is superior on all problems [66], the choice of AOO must be justified empirically on the problem at hand rather than by benchmark results reported elsewhere. Five optimizers were therefore run on the identical 66-parameter task with the identical evaluation budget: AOO; HHO [43], a swarm-based algorithm widely used as a comparison baseline and previously combined with ANFIS [60,61,62,63,64,65]; particle swarm optimization (PSO) [67] and a real-coded genetic algorithm (GA) [68], the two long-established population-based methods most often used to tune ANFIS [45,49,56]; and the covariance matrix adaptation evolution strategy (CMA-ES) [69], which is generally regarded as the state-of-the-art evolutionary method for continuous non-separable problems of moderate dimension and adapts its search distribution to the local curvature of the objective. This design allows the question raised in [66], which is whether a recently proposed metaheuristic offers a measurable advantage over established alternatives on a given problem, to be answered directly for the HST controller design problem.

The main contributions of this study are summarized as follows:

1. AOO-ANFIS, a novel controller architecture in which the complete 66-dimensional premise-and-consequent parameter vector of a two-input, two-output first-order Takagi–Sugeno ANFIS with parameters tuned by the AOO algorithm as a single black-box optimization problem, is proposed. AOO has previously been benchmarked as one of several metaheuristics tuning a cascaded simplified PD-like fuzzy logic controller integrated with a fractional-order PID controller in a power-electronics application [38]. AOO-ANFIS is consequently a new, previously unproposed architecture rather than an incremental variation of existing metaheuristic-ANFIS hybrids.
2. This is also the first study to apply any bio-inspired-optimization-tuned ANFIS controller to hydrostatic transmission stroke control, extending a control problem previously addressed with MMPC [18] into the neuro-fuzzy domain.
3. AOO is compared with HHO, PSO, GA and CMA-ES on the identical tuning task and evaluation budget, with statistical significance assessed by Wilcoxon rank-sum tests with Holm correction. AOO attains the lowest mean validation error among the population-based optimizers in terms of being significantly lower than HHO, GA and CMA-ES; statistically tied with PSO.
4. The AOO-ANFIS controller is validated in processor-in-the-loop simulation on a BeagleBone Black target, manufactured by BeagleBoard.org Foundation, MI, USA, under the deterministic and stochastic disturbance profiles used for the previously published MMPC [18] and ANN [19], enabling a direct, with transient, integral-error, control-effort and computational-load metrics, like-for-like performance comparison. Its generalization beyond the training data is examined in an extended 300 s scenario with unseen reference levels, ramp rates, a sinusoidal segment and trapezoidal disturbances, in comparison with the ANN surrogate of [19], and its sensitivity to a 15% degradation of the pump and motor volumetric efficiencies is quantified for both surrogates.

## 2. Materials and Methods

### 2.1. Hydrostatic Transmission Plant

**Figure 1:**
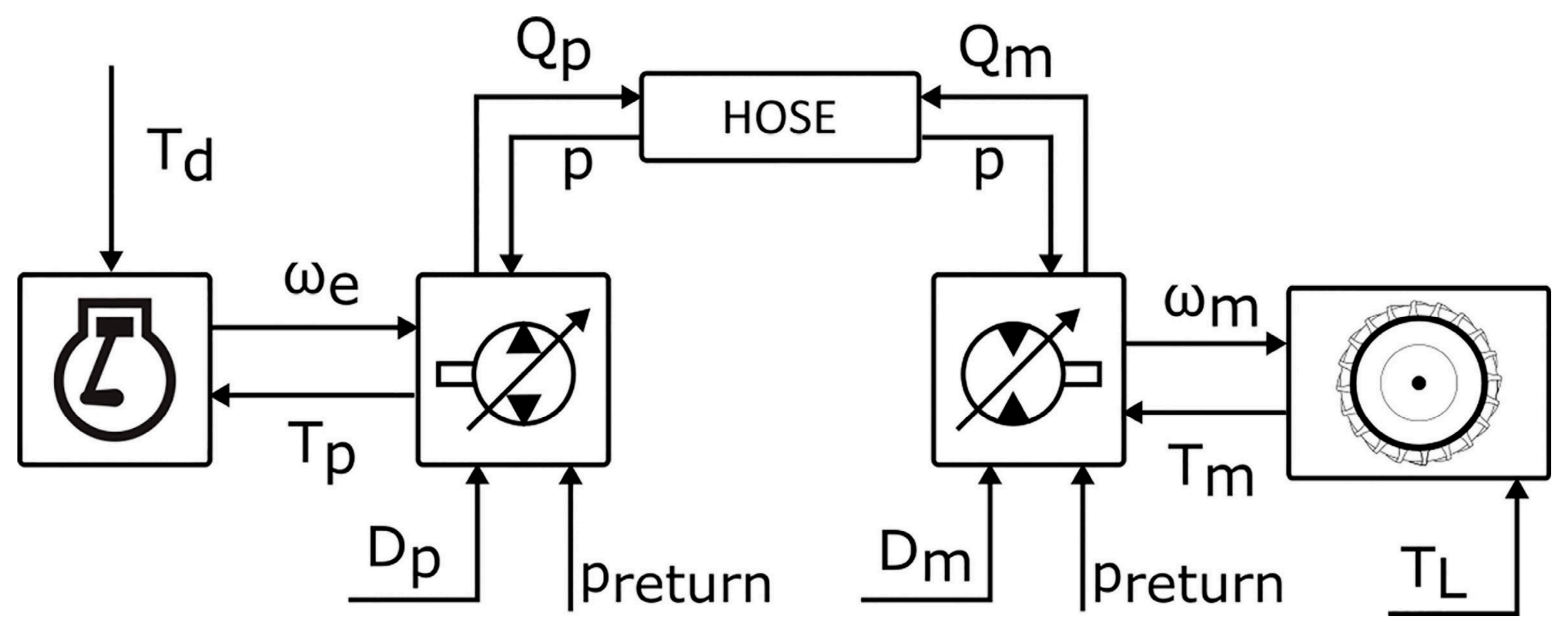
Block diagram of the HST system [18,19].

The nonlinear dynamics of the HST system [8] in Figure 1 utilizing variable-displacement hydraulic units are formulated in accordance with the foundational framework established in [18,19]. The system dynamics are dictated by three fundamental state variables. Owing to the rigid shaft connection between the primary mover (diesel engine) and the hydraulic pump, the engine rotational speed $\omega_{e}$ directly matches the pump shaft speed $\omega_{p}$. To simplify the rotational dynamic analysis, the moments of inertia of the engine $J_{d}$ and the pump $J_{p}$ are merged into a combined equivalent inertia $J_{pd}$. The dynamic differential equation representing the pump side is expressed in Equation (1).

(1) $${\dot{\omega }}_{p}=\frac{1}{J_{pd}}\left(T_{d}-\mu_{p}\;\omega_{p}-K_{p}-\frac{D_{p}\;∆p}{\eta_{tp}}\right)$$

where $T_{d}$ represents the torque generated by the engine, $\mu_{p}$ and $K_{p}$ denote the Coulomb friction coefficient and viscous friction parameter of the pump shaft, respectively, $D_{p}$ indicates the pump displacement, ∆p defines the differential pressure between the high-pressure working line and return line and $\eta_{tp}$ signifies the mechanical efficiency of the pump unit. On the actuator side, the vehicle drive dynamic properties are integrated by combining the hydraulic motor inertia $J_{m}$ and the wheel inertia $J_{w}$ into a unified lumped parameter $J_{mw}$. Because the vehicle operational velocity is strictly tied to the rotation of the hydraulic motor shaft $\omega_{m}$, the governing differential equation for the motor side is given by

(2) $${\dot{\omega }}_{m}=\frac{1}{J_{mw}}\left[\eta_{tm}D_{m}\;∆p-\mu_{m}\;\omega_{m}-K_{m}\;sign(\omega_{m})-T_{L}\right]$$

where $T_{L}$ denotes the total external load torque imposed on the vehicle, $\mu_{m}$ is the motor shaft Coulomb friction, $K_{m}\;sign\left(\omega_{m}\right)$ models the directional viscous resistance, $D_{m}$ is the variable displacement of the motor and $\eta_{tm}$ represents the mechanical efficiency of the motor unit. The transient behavior of the working fluid within the high-pressure line establishes the pressure differential equation as

(3) $$∆\dot{p}=\frac{\beta \;D_{p}\omega_{p}\eta_{vp}}{V}-\frac{\beta \;\omega_{m}\;D_{m}}{V\;\eta_{vm}}-{\dot{p}}_{return}$$

where V corresponds to the internal volume of the connecting hose, β denotes the effective bulk modulus of the fluid and $\eta_{vp}$ and $\eta_{vm}$ represent the volumetric efficiencies of the pump and motor, respectively, which depend on operating speed and displacement settings. The pressure rate ${\dot{p}}_{return}$ reflects variations in the low-pressure return line, maintained by an auxiliary flushing pump to minimize thermal accumulation and particulate contamination. Equations (1)–(3) are cast into a control-affine nonlinear state-space formulation in Equation (4).

(4) $$\begin{array}{c} \left[\begin{array}{c} \dot{p}\\ {\dot{\omega }}_{m}\\ {\dot{\omega }}_{p} \end{array}\right]=\left[\begin{array}{c} 0\\ -\frac{\mu_{m}\;}{J_{mw}}\omega_{m}-\frac{K_{m}\;}{J_{mw}}sign(\omega_{m})\\ -\frac{\mu_{p}\;}{J_{pd}}\omega_{p}-\frac{K_{p}}{J_{pd}} \end{array}\right]+\left[\begin{array}{cc} \frac{\beta \;\eta_{vp}}{V}\omega_{p} & -\frac{\;\beta \;}{V\;\eta_{vm}}\omega_{m}\\ 0 & \frac{\;\eta_{tm}}{J_{mw}\;}p\\ -\frac{\;1}{J_{pd}\;\eta_{tp}}p & 0 \end{array}\right]\left[\begin{array}{c} D_{p}\\ D_{m} \end{array}\right]\\ \;+\left[\begin{array}{cc} 0 & 0\\ 0 & -\frac{1}{J_{mw}}\\ \frac{1}{J_{pd}} & 0 \end{array}\right]\left[\begin{array}{c} T_{d}\\ T_{L} \end{array}\right]\; \end{array}$$

Under standard operating regimes characterized by low-speed forward operation, the directional dependence of friction simplifies, rendering $sign(\omega_{m})$ unnecessary, while torque efficiencies can be approximated as constant values. Furthermore, assuming a constant low-pressure line state ($p_{return}=20\;bar$), its derivative vanishes (${\dot{p}}_{return}=0$).

The values of all HST plant parameters and time-varying parameters of volumetric efficiencies of the pump and motor modeled using two-dimensional lookup tables were obtained from Carlsson [8] as in previous studies [18,19].

### 2.2. Adaptive Neuro-Fuzzy Inference System

#### 2.2.1. Fuzzy Inference Models and the ANFIS Architecture

Two fuzzy inference paradigms dominate the control-engineering literature: the Mamdani model, in which both the antecedent and the consequent of each rule are fuzzy sets combined through a fuzzy implication [70], and the Takagi–Sugeno (T–S) model, in which the consequent of each rule is a crisp function (typically affine) of the inputs rather than a fuzzy set [71]. The T–S formulation is preferred whenever the fuzzy system must be tuned from numerical data and embedded in a real-time control loop, because its output is a closed-form, differentiable combination of the input variables and does not require a defuzzification step, which reduces both the computational burden and the number of design choices (defuzzification method, output fuzzy-set shapes) relative to a Mamdani system [20,71]. For a two-input, two-rule T–S system, the rule base takes the form

(5) $$\mathrm{Rule}\;k:\;\mathrm{IF}\;x_{1}\;\mathrm{is}\;A_{k}\;\mathrm{AND}\;x_{2}\;\mathrm{is}\;B_{k}\;\mathrm{THEN}\;f_{k}=p_{k}x_{1}+q_{k}x_{2}+r_{k},k=1,2,$$

where $x_{1},x_{2}$ are the crisp inputs, $A_{k}$ and $B_{k}$ are fuzzy sets described by membership functions, and $p_{k},q_{k},r_{k}$ are the consequent coefficients of the k-th rule’s linear output function $f_{k}$.

Jang’s ANFIS [20] implements a first-order T-S model as a five-layer feed-forward adaptive network whose parameters can be identified from input–output data using gradient-based learning. For the illustrative two-rule case of Equation (5), the layer-by-layer computation is [20]

(6) $$\mathrm{Layer}\;1\;(\mathrm{fuzzification}):\;O_{1,i}=\mu_{A_{i}}\left(x_{1}\right),\;i=1,2;\;O_{1,i}=\mu_{B_{i-2}}\left(x_{2}\right),\;i=3,4,$$

(7) $$\mathrm{Layer}\;2\;(\mathrm{rule}\;\mathrm{firing}\;\mathrm{strength}):\;O_{2,k}=w_{k}=\mu_{A_{k}}\left(x_{1}\right)\;\mu_{B_{k}}\left(x_{2}\right),\;\;k=1,2,$$

(8) $$\mathrm{Layer}\;3\;(\mathrm{normalization}):\;O_{3,k}=\bar{w_{k}}=\frac{w_{k}}{w_{1}+w_{2}},\;\;k=1,2,$$

(9) $$\mathrm{Layer}\;4\;(\mathrm{defuzzification}):\;O_{4,k}=\bar{w_{k}}f_{k}=\bar{w_{k}}\left(p_{k}x_{1}+q_{k}x_{2}+r_{k}\right),\;\;k=1,2,$$

(10) $$\mathrm{Layer}\;5\;(\mathrm{aggregation}):\;O_{5}=\sum_{k=1}^{2}\bar{w_{k}}f_{k},$$

where $\mu_{A_{i}}\left(\cdot \right)$ and $\mu_{B_{i}}\left(\cdot \right)$ denote the membership degree of an input in the i-th fuzzy set of the corresponding antecedent variable, $O_{l,i}$ denotes the output of the i-th node in layer l, $w_{k}$ is the (unnormalized) firing strength of rule k, $\bar{w_{k}}$ is its normalized firing strength and $O_{5}$ is the overall crisp output of the network. In ANFIS’s original hybrid learning rule, the consequent parameters $p_{k},q_{k},r_{k}$ are identified in closed form by least-squares estimation during a forward pass, while the membership-function parameters are updated by gradient descent during a backward pass [20]. This hybrid scheme is a local search procedure: because the backward pass is a gradient-based update, its outcome depends on the initial parameter values, and it can converge to a poor local optimum on nonconvex, multimodal error surfaces.

#### 2.2.2. Two-Output Sugeno Structure

The controller designed in this study must produce two simultaneous outputs, $D_{p}$ and $D_{m}$, whereas the classical ANFIS architecture in [20] is defined for a single output. Therefore, a single multi-output first-order Sugeno fuzzy inference system with two inputs ($i_{s}$, $i_{s,ref}$) and two outputs ($D_{p}$, $D_{m}$) is constructed, rather than training two independent single-output ANFIS networks; this keeps the controller as a single fuzzy-logic unit.

Each input is described by M = 3 Gaussian membership functions,

(11) $$\mu_{A_{j}}\left(x_{i}\right)=\exp\left(-\frac{{\left(x_{i}-c_{i,j}\right)}^{2}}{2\sigma_{i,j}^{2}}\right),\;\;i\in \{1,2\},\;j=1,\ldots ,M,$$

where $x_{i}$ denotes the i-th input ($x_{1}=i_{s}$, $x_{2}=i_{s,ref}$), $\mu_{A_{j}}\left(x_{i}\right)$ is the membership degree of $x_{i}$ in the j-th fuzzy set of input i and $c_{i,j}$ and $\sigma_{i,j}$ are, respectively, the center and standard deviation of the j-th Gaussian membership function of input i. With three membership functions per input, the rule base has $R=M^{2}=9$ rules, one for every combination of input-1 and input-2 membership functions. The firing strength of rule k is the product of the two membership degrees selected by that rule,

(12) $$w_{k}=\mu_{A_{j}}\left(i_{s}\right)\;\mu_{B_{l}}\left(i_{s,ref}\right),\;\;k=1,\ldots ,R,$$

where j and l index the input-1 and input-2 membership functions assigned to rule k. The firing strengths are normalized as

(13) $$\bar{w_{k}}=\frac{w_{k}}{\sum_{n=1}^{R}w_{n}},$$

where $\bar{w_{k}}$ is the normalized firing strength of rule k, i.e., its relative contribution to the overall output. Each rule produces two first-order linear consequent outputs, one per controller output,

(14) $$f_{k}^{D_{p}}=a_{k}^{D_{p}}i_{s}+b_{k}^{D_{p}}i_{s,ref}+c_{k}^{D_{p}},\;\;\;\;\;\;f_{k}^{D_{m}}=a_{k}^{D_{m}}i_{s}+b_{k}^{D_{m}}i_{s,ref}+c_{k}^{D_{m}},$$

where $a_{k}^{\left(\cdot \right)},b_{k}^{\left(\cdot \right)},c_{k}^{\left(\cdot \right)}$ are the consequent coefficients of rule k for the corresponding output. The two crisp controller outputs are obtained by the weighted sum of the rule outputs,

(15) $$D_{p}=\sum_{k=1}^{R}\bar{w_{k}}f_{k}^{D_{p}},\;\;D_{m}=\sum_{k=1}^{R}\bar{w_{k}}f_{k}^{D_{m}}.$$

The complete set of tunable parameters of the resulting structure comprises 12 premise parameters (Equation (11): $c_{i,j},\sigma_{i,j}$ for i=1,2 and j=1,2,3) and 54 consequent parameters (Equation (14): 3 coefficients × 2 outputs × 9 rules), for a total of D = 66 real-valued parameters, collected into a single decision vector $x\in R^{66}$ for optimization.

#### 2.2.3. The Surrogate-Controller Formulation

The AOO-ANFIS controller does not observe the HST model and does not solve an optimization problem online. It is a surrogate controller, which means a static map from the measured stroke $i_{s}$ and its reference $i_{s,ref}$ to the two displacement commands ($D_{p}$, $D_{m}$) that is fitted, in a supervised manner, to the closed-loop behavior of the MMPC of [18]. The transfer of information from the MMPC to the ANFIS proceeds in three steps.

Step 1 is data generation: The MMPC was run in closed loop on the validated nonlinear HST model under the reference trajectory of [18]. At every controller sampling instant, the pair of controller inputs ($i_{s}$, $i_{s,ref}$) and the pair of MMPC outputs ($D_{p}$, $D_{m}$) were logged. Each sample is thus an example of the action the MMPC took in a particular state of the closed loop; the dataset is the same one used to train the ANN surrogate in [19].

Step 2 is supervised tuning: The ANFIS parameters are chosen so that the fuzzy system’s outputs reproduce the MMPC outputs on unseen samples. The fitness of a candidate parameter vector in Equation (39) is the validation-set mean squared error between the fuzzy outputs and the MMPC targets; no closed-loop simulation is required during tuning, so every fitness evaluation costs only a forward pass of the fuzzy system. This is the learning of an approximate MPC or MPC policy imitation paradigm [72,73], in which an expensive predictive controller is used offline as a teacher for a cheap parametric controller.

Step 3 is stand-alone deployment: After tuning, the fuzzy system is placed in the loop, replacing the MMPC in the closed-loop configuration of Section 3.2. The MMPC, its internal plant models and its optimizer are no longer part of the running controller. Consequently, the online cost of the AOO-ANFIS is that of evaluating nine Gaussian rules, independent of the prediction horizon and of the number of local models of the MMPC.

Two consequences of this formulation are important for interpreting the results. First, because the fuzzy system is a smooth, low-order approximation of the MMPC’s policy, it inherits the MMPC’s steady-state behavior but regularizes its transient behavior; the switching between the MMPC’s local models and the receding-horizon re-optimization at every sample produce a control signal with rapid, small-amplitude changes, whereas the nine-rule fuzzy interpolation produces a control signal that varies smoothly with ($i_{s}$, $i_{s,ref}$). This is why a surrogate can, in some metrics, outperform its teacher. Second, the surrogate is only as good as the region of the input space covered by the training data; its behavior outside that region is an extrapolation.

### 2.3. Bio-Inspired Optimization Framework for ANFIS Tuning

Jang’s hybrid learning rule updates the antecedent parameters by gradient descent [20], a local search procedure whose outcome depends on the initial parameter values and which can converge to a poor local optimum, particularly for nonconvex, multimodal error surfaces. A growing body of literature has consequently replaced this gradient-based update, in whole or in part, with population-based metaheuristic optimization: recent examples include a systematic hybridization framework combining ANFIS with ant colony optimization, particle swarm optimization, a genetic algorithm and simulated annealing [49]; a hybrid ANFIS–particle-swarm-optimization model for estimating the load-carrying characteristics of porous air bearings, benchmarked directly against an ANN [56]; and a hybrid ANFIS–Marine-Predators-Algorithm model for time-series forecasting published in this journal [59]. Metaheuristic training has also been reported to alleviate the local-minimum trap of gradient descent in classification settings using, e.g., an Artificial Bee Colony optimizer in place of back-propagation [74]. Furthermore, because no single optimization algorithm can be guaranteed to outperform all others across every possible problem instance [66], the present study evaluates several different nature-inspired algorithms on the same ANFIS-tuning task rather than committing to one a priori.

Following this line of work, the present study replaces the gradient-based antecedent update and, for consistency, the least-squares consequent update with a population-based, nature-inspired metaheuristic that searches the full 66-dimensional parameter vector x as a single black-box optimization problem, without distinguishing antecedent from consequent parameters during the search. Every algorithm below operates on the same generic population $\left\{x_{1},\ldots ,x_{N}\right\}$ of N candidate decision vectors, refined over T iterations, indexed by t=1,…,T; the vector $x_{\mathrm{bs}}^{t}$ denotes the best, which means lowest-fitness, candidate found up to and including iteration t.

Five population-based, nature-inspired metaheuristic algorithms were evaluated as candidate optimizers for the ANFIS parameter vector: HHO [43], a well-established swarm-based metaheuristic widely used as a comparison baseline in the recent optimization literature, and the AOO [33], a recently proposed plant-inspired metaheuristic, together with three long-established baselines such as PSO, a real-coded GA and CMA-ES. All algorithms were run with the same population size N and the same maximum number of iterations T, and their performance was evaluated using the identical fitness function and the identical encoding of the decision vector, for a fair comparison. Each algorithm used a population of *N* = 30 candidates and *T* = 100 iterations, and was run independently 10 times. With these settings, each run evaluates approximately 3000 candidate controllers; HHO requires somewhat more evaluations because two candidates are evaluated in each of its rapid-dive phases. All settings are listed in Table A1 (Appendix A).

#### 2.3.1. Animated Oat Optimization Algorithm (AOO)

AOO models the seed-dispersal strategy of the plant *Avena sterilis*, which combines wind-, water- and animal-mediated dispersal, hygroscopic-awn-driven rolling in the absence of obstacles, and energy-storing ejection upon encountering an obstacle [33]. At every iteration, four auxiliary quantities are recomputed for each candidate,

(16) $$m=\frac{0.5\;r}{d},\;\;L=\frac{N\;r}{d},\;\;e=\frac{0.5\;r}{d},\;\;c=1-{\left(\frac{t}{T}\right)}^{3},$$

where d is the dimensionality of the decision vector, which is d=66 in this study, $r\in \left[0,1\right]$ denotes a freshly drawn uniform random number for each of m, L and e, m and L are, respectively, the modeled mass and main-awn length of the seed, e is the eccentric rotation coefficient during rolling and c is a dynamic adjustment factor that decreases monotonically from 1 to 0 as t→T, shifting the search from exploration toward exploitation as the run progresses.

Dispersal (exploration): With probability 0.5, a candidate updates its position as

(17) $$x_{i}^{t+1}=\left\{\begin{array}{cc} \frac{1}{N}\sum_{n=1}^{N}x_{n}^{t}+W, & \mathrm{if}\;i\;mod\left\lfloor N/10\right\rfloor =0,\\ x_{\mathrm{best}}^{t}+W, & \mathrm{if}\;i\;mod\left\lfloor N/10\right\rfloor =1,\\ x_{i}^{t}+W, & \mathrm{otherwise}, \end{array}\right.\;W=\frac{c}{\pi }\left(2r-1\right)⊗ub$$

where r is a vector of independent uniform random numbers in $\left[0,1\right]$ with the same dimensionality as x, 1 is the all-ones vector, ub is the vector of upper bounds of the search space, ⊗ denotes the element-wise (Hadamard) product and W is the resulting random dispersal step.

Rolling (exploitation without obstacles): With the complementary probability, and with a further probability of 0.5, a candidate rolls under hygroscopic-awn action,

(18) $$A=ub-\left|ub\cdot t\cdot sin(2\pi r)/T\right|,\;\;R=\frac{\left(m\;e+L^{2}\right)}{d}\;u\left(-A,A\right),$$

(19) $$\mathrm{Levy}\left(d\right)=0.01\;\frac{\mu \;\sigma }{{\left|v\right|}^{1/\beta }},\;\;\;\sigma ={\left[\frac{\Gamma \left(1+\beta \right)\sin\left(\pi \beta /2\right)}{\Gamma \left(\frac{1+\beta }{2}\right)\;\beta \;2^{\left(\beta -1\right)/2}}\right]}^{1/\beta }$$

(20) $$x_{i}^{t+1}=x_{\mathrm{best}}^{t}+R+c\cdot \mathrm{Levy}\left(d\right)⊗x_{\mathrm{best}}^{t},$$

where A is an iteration-dependent bound on the rolling amplitude, $r\in \left[0,1\right]$ is a uniform random number, $u\left(-A,A\right)$ is a vector of independent uniform random numbers on $\left[-A,A\right]$ of dimensionality d, $\mathrm{Levy}\left(d\right)$ is a Lévy-flight step vector, $\mu \in \left[0,1\right]$ is a random scale factor, v is the velocity vector, β=1.5 is the stability-distribution shape parameter and $\Gamma \left(\cdot \right)$ is the Gamma function.

Ejection (exploitation upon an obstacle): Otherwise, the candidate models a spring-driven projectile ejection,

(21) $$\begin{array}{c} B=ub-\left|ub\cdot t\cdot cos(2\pi r)/T\right|\;,\\ \;\;\;\;\;\;\;\;\;\;\;\;\;\;\;\;\;\;\;\;\;\;\;\;k=0.5+0.5r,\;x=\frac{3r}{d},\;\theta =\pi r,\;\alpha =\frac{1}{\pi }\exp\left(\frac{r^{\prime }}{T}\right), \end{array}$$

(22) $$J=\frac{2k\;x^{2}\sin\left(2\theta \right)}{m\;g}\cdot \frac{u\left(-B,B\right)}{d}\cdot \left(1-\alpha \right),\;\;\;\;\;x_{i}^{t+1}=x_{\mathrm{best}}^{t}+J+c\cdot \mathrm{Levy}\left(d\right)⊗x_{\mathrm{best}}^{t}$$

where B is an iteration-dependent bound on the ejection amplitude, k is the modeled elasticity coefficient of the main awn, x is the modeled awn extension during energy storage, θ is the ejection angle relative to the ground, α is an air-resistance coefficient with $r^{\prime }$ a uniform random numberon $\left[0,T\right]$, g is the gravitational acceleration constant [42] and J is the resulting ejection displacement. In all three mechanisms, each candidate’s fitness is evaluated after the update, and the population and $x_{\mathrm{best}}^{t}$ are updated.

#### 2.3.2. Harris Hawks Optimization (HHO)

HHO models the cooperative surprise pounce hunting strategy of Harris’ hawks, in which the search alternates between exploration and exploitation according to the prey’s escaping energy [43]. The decreasing energy of the prey and the resulting escaping energy are

(23) $$E_{1}=2\left(1-\frac{t}{T}\right),\;\;E=E_{1}E_{0},\;\;E_{0}∼U\left(-1,1\right),$$

where $E_{1}$ decreases linearly from 2 to 0 over the run, $E_{0}$ is drawn anew at every iteration and E is the resulting escaping energy of the prey at iteration t. When $\left|E\right|\geq 1$, the population explores

(24) $$x_{i}^{t+1}=\left\{\begin{array}{cc} x_{\mathrm{rand}}^{t}-r_{1}\left|x_{\mathrm{rand}}^{t}-2r_{2}x_{i}^{t}\right|, & q<0.5\\ \left(x_{\mathrm{best}}^{t}-x_{m}^{t}\right)-r_{3}\left(lb+r_{4}\left(ub-lb\right)\right), & q\geq 0.5 \end{array}\right.$$

where $q,r_{1},r_{2},r_{3},r_{4}\in \left[0,1\right]$ are independent uniform random numbers, $x_{\mathrm{rand}}^{t}$ is a randomly selected member of the population, $x_{m}^{t}$ is the mean position of the current population and lb,ub are the lower and upper bounds of the search space, and r ∈ [0,1] is a random number denoting the chance of the prey escaping. When $\left|E\right|<1$, the population exploits. For r≥0.5 and $\left|E\right|\geq 0.5$,

(25) $$x_{i}^{t+1}=\Delta x_{i}^{t}-E\left|J\;x_{\mathrm{best}}^{t}-x_{i}^{t}\right|,\;\;\Delta x_{i}^{t}=x_{\mathrm{best}}^{t}-x_{i}^{t},\;\;J=2\left(1-r_{5}\right),$$

where $\Delta x_{i}^{t}$ is the distance between the best solution and candidate i, J is a random jump strength representing the prey’s erratic motion and $r_{5}\in \left[0,1\right]$ is a uniform random number. For r≥0.5 and $\left|E\right|<0.5$,

(26) $$x_{i}^{t+1}=x_{\mathrm{best}}^{t}-E\left|\Delta x_{i}^{t}\right|.$$

For r<0.5 and $\left|E\right|\geq 0.5$, two candidate positions are generated,

(27) $$y=x_{\mathrm{best}}^{t}-E\left|J\;x_{\mathrm{best}}^{t}-x_{i}^{t}\right|,\;\;z=y+s⊗\mathrm{LF}\left(d\right),$$

where s is a random vector and $\mathrm{LF}\left(d\right)$ is a Lévy-flight step vector of dimensionality d. For r<0.5 and $\left|E\right|<0.5$, the same construction is applied around the population mean,

(28) $$y=x_{\mathrm{best}}^{t}-E\left|J\;x_{\mathrm{best}}^{t}-x_{m}^{t}\right|,\;\;\;\;\;\;z=y+s⊗\mathrm{LF}\left(d\right),$$

In both rapid-dive cases, the candidate here greedily adopts whichever of y, z or its current position $x_{i}^{t}$ has the lowest fitness,

(29) $$x_{i}^{t+1}=\left\{\begin{array}{cc} y, & J\left(y\right)<J\left(x_{i}^{t}\right),\\ z, & J\left(z\right)<J\left(x_{i}^{t}\right)\;\mathrm{and}\;J\left(z\right)<J\left(y\right),\\ x_{i}^{t}, & \mathrm{otherwise}, \end{array}\right.$$

where $J\left(\cdot \right)$ denotes the fitness function value of the candidate position in question.

#### 2.3.3. Particle Swarm Optimization (PSO)

PSO models a swarm of N particles that move through the search space under the combined influence of their own experience and that of their neighbors [67]. Each particle i has a position $x_{i}^{t}$, a velocity $v_{i}^{t}$ and a personal best position $p_{i}^{t}$, i.e., the lowest-fitness position it has visited up to iteration t. The velocity and position are updated as follows:

(30) $$v_{i}^{t+1}=W^{t}\;v_{i}^{t}+c_{1}\;r_{1}⊗\left(p_{i}^{t}-x_{i}^{t}\right)+c_{2}\;r_{2}⊗\left(g_{i}^{t}-x_{i}^{t}\right),\;\;x_{i}^{t+1}=x_{i}^{t}+v_{i}^{t+1},$$

where $W^{t}$ is the inertia weight [75], $c_{1}$ and $c_{2}$ are the cognitive or self-adjustment and social-adjustment coefficients, $r_{1}$ and $r_{2}$ are vectors of independent uniform random numbers in $\left[0,1\right]$ of dimension d, ⊗ denotes the element-wise product and $g_{i}^{t}$ is the best position found within the neighborhood of particle i. In the implementation used [76], the neighborhood is a randomly selected subset of the swarm whose initial size is $max\left(2,\lfloor 0.25N\rfloor \right)=7$ particles, and the inertia weight is adapted according to a stall counter s that records the number of consecutive iterations without improvement in the swarm’s best fitness:

(31) $$W^{t+1}=\left\{\begin{array}{ll} min\left(2W^{t},\;W_{max}\right), & s<2,\\ max\left(W^{t}/2,\;W_{min}\right), & s>5,\\ W^{t}, & \mathrm{otherwise}, \end{array}\right.$$

with $\left[W_{min},W_{max}\right]=\left[0.1,1.1\right]$. Thus, the swarm becomes more exploratory while it keeps improving, and more exploitative when it stalls. Initial positions are drawn uniformly within the bounds and initial velocities uniformly within $\left[-\left(ub-lb\right),\;ub-lb\right]$. A component of $x_{i}^{t+1}$ that leaves the bounds is set to the violated bound, and the corresponding velocity component is set to zero if it points outwards. The coefficients were $c_{1}=c_{2}=1.49$.

#### 2.3.4. Genetic Algorithm (GA)

The GA evolves a population of real-valued chromosomes, each being a candidate vector x, through selection, recombination and mutation inspired by natural evolution [68]. At each generation, the raw fitness values are converted into expectation values by rank-based scaling, and parents are chosen by tournament selection, in which the best of four randomly drawn individuals is selected [76]. The next generation of N individuals is composed of E elite individuals copied unchanged, $n_{c}=round\left(p_{c}\left(N-E\right)\right)$ crossover children and $N-E-n_{c}$ mutation children, where $p_{c}$ is the crossover fraction. With N=30, E=2 and $p_{c}=0.8$, each generation contains 2 elite, 22 crossover and 6 mutation children. Crossover is performed by the scattered (uniform) operator,

(32) $$x_{\mathrm{child},j}=\left\{\begin{array}{ll} x_{\mathrm{parent}\;1,j}, & b_{j}=1,\\ x_{\mathrm{parent}\;2,j}, & b_{j}=0, \end{array}\right.\;\;j=1,\ldots ,d,$$

where $b_{j}$ is an independent Bernoulli variable with probability 0.5, so that each gene of the child is inherited from one of the two parents. Mutation children are generated by the adaptive-feasible operator of [76], which perturbs a parent along a randomly generated direction whose orientation adapts to the success of the previous generation, with a step length chosen such that the child remains within the bounds.

#### 2.3.5. Covariance Matrix Adaptation Evolution Strategy (CMA-ES)

CMA-ES samples candidate solutions from a multivariate normal distribution and adapts the mean, the global step size and the full covariance matrix of that distribution from the ranking of the sampled candidates [69,77,78]. This makes it invariant to rotations of the search space and particularly effective on non-separable, ill-conditioned problems. At generation t, λ candidates are sampled around the mean $m^{t}$:

(33) $$x_{k}^{t}=m^{t}+\sigma^{t}B^{t}D^{t}z_{k},\;\;z_{k}∼N\left(0,I\right),\;\;k=1,\ldots ,\lambda ,$$

where $\sigma^{t}$ is the global step size and $C^{t}=B^{t}{\left(D^{t}\right)}^{2}{\left(B^{t}\right)}^{T}$ is the eigen-decomposition of the covariance matrix. Each candidate is projected onto the box $\left[lb,ub\right]$ before evaluation, and the projected candidates are used in the updates below. The candidates are sorted by fitness, $x_{1:\lambda }^{t},\ldots ,x_{\lambda :\lambda }^{t}$, and the mean is moved to a weighted recombination of the best μ=λ/2 of them:

(34) $$m^{t+1}=\sum_{i=1}^{\mu }w_{i}\;x_{i:\lambda }^{t},\;\;w_{i}=\frac{ln\left(\mu +1/2\right)-lni}{\sum_{j=1}^{\mu }\left(ln\left(\mu +1/2\right)-lnj\right)},\;\;\mu_{\mathrm{eff}}={\left(\sum_{i=1}^{\mu }w_{i}^{2}\right)}^{-1}.$$

Two evolution paths accumulate the successive mean shifts:

(35) $$p_{\sigma }^{t+1}=\left(1-c_{\sigma }\right)\;p_{\sigma }^{t}+\sqrt{c_{\sigma }\left(2-c_{\sigma }\right)\;\mu_{\mathrm{eff}}}\;{\left(C^{t}\right)}^{-1/2}\;\frac{m^{t+1}-m^{t}}{\sigma^{t}},$$

(36) $$p_{c}^{t+1}=\left(1-c_{c}\right)\;p_{c}^{t}+h_{\sigma }\sqrt{c_{c}\left(2-c_{c}\right)\;\mu_{\mathrm{eff}}}\;\frac{m^{t+1}-m^{t}}{\sigma^{t}},$$

where $h_{\sigma }\in \{0,1\}$ stalls the update of $p_{c}$ when $∥p_{\sigma }∥$ is large [78]. The covariance matrix is updated by a rank-one term built from $p_{c}$ and a rank-μ term built from the selected steps [77],

(37) $$\begin{array}{c} C^{t+1}=\left(1-c_{1}-c_{\mu }\right)\;C^{t}+c_{1}\left(p_{c}^{t+1}{\left(p_{c}^{t+1}\right)}^{T}+\left(1-h_{\sigma }\right)\;c_{c}\left(2-c_{c}\right)\;C^{t}\right)\\ +c_{\mu }\sum_{i=1}^{\mu }w_{i}\;y_{i}\;y_{i}^{T},\;\;y_{i}=\frac{x_{i:\lambda }^{t}-m^{t}}{\sigma^{t}}, \end{array}$$

and the step size by cumulative step-size adaptation,

(38) $$\sigma^{t+1}=\sigma^{t}exp\left(\frac{c_{\sigma }}{d_{\sigma }}\left(\frac{∥p_{\sigma }^{t+1}∥}{E∥N\left(0,I\right)∥}-1\right)\right).$$

The learning rates were set to the standard default values of the CMA-ES, which follow the scalings $c_{1}\approx 2/d^{2}$ and $c_{\mu }\approx \mu_{\mathrm{eff}}/d^{2}$ [78], namely $c_{c}=\frac{4+\mu_{\mathrm{eff}}/d}{d+4+2\mu_{\mathrm{eff}}/d}$, $c_{\sigma }=\frac{\mu_{\mathrm{eff}}+2}{d+\mu_{\mathrm{eff}}+5}$, $c_{1}=\frac{2}{{\left(d+1.3\right)}^{2}+\mu_{\mathrm{eff}}}$, $c_{\mu }=min\left(1-c_{1},\;\frac{2\left(\mu_{\mathrm{eff}}-2+1/\mu_{\mathrm{eff}}\right)}{{\left(d+2\right)}^{2}+\mu_{\mathrm{eff}}}\right)$ and $d_{\sigma }=1+2max\left(0,\sqrt{\frac{\mu_{\mathrm{eff}}-1}{d+1}}-1\right)+c_{\sigma }$. For d=66 and λ=30 (μ=15, $\mu_{\mathrm{eff}}=8.64$), these give $c_{c}=0.059$, $c_{\sigma }=0.134$, $c_{1}=4.4\times 10^{-4}$, $c_{\mu }=2.9\times 10^{-3}$ and $d_{\sigma }=1.13$. The initial mean was drawn uniformly within the bounds, and the initial step size was $\sigma^{0}=0.3$ times the mean width of the search intervals ($\sigma^{0}=1.39$). The covariance learning rates imply an adaptation time scale of the order of $1/\left(c_{1}+c_{\mu }\right)\approx 300$ generations, longer than the 100 generations available, so within this budget CMA-ES operates mainly through its mean and step-size adaptation.

#### 2.3.6. Decision-Vector Encoding

Each candidate solution produced by an optimization algorithm is a real-valued vector $x=\left[x_{1},\ldots ,x_{66}\right]$, decoded into the ANFIS parameters of Equations (11) and (14) as summarized in Table 1.

**Table 1:** Encoding of the 66-dimensional ANFIS parameter vector optimized by each algorithm.

Segment	Indices	Parameters	Count	Bounds
$i_{s}$ centers	$x_{1:3}$	$c_{1,1},c_{1,2},c_{1,3}$	3	$\left[-1,1\right]$
$i_{s}$ widths	$x_{4:6}$	$\sigma_{1,1},\sigma_{1,2},\sigma_{1,3}$	3	$\left[0.15,1.0\right]$
$i_{s,ref}$ centers	$x_{7:9}$	$c_{2,1},c_{2,2},c_{2,3}$	3	$\left[-1,1\right]$
$i_{s,ref}$ widths	$x_{10:12}$	$\sigma_{2,1},\sigma_{2,2},\sigma_{2,3}$	3	$\left[0.15,1.0\right]$
Consequent coefficients	$x_{13:66}$	$a_{k}^{\left(\cdot \right)},b_{k}^{\left(\cdot \right)},c_{k}^{\left(\cdot \right)},k=1,\ldots ,9$	54	$a,b\in \left[-3,3\right];c\in \left[-2,2\right]$

The center and width bounds correspond to the min–max-normalized input domain, and the consequent-coefficient bounds were chosen wide enough not to constrain the attainable output range while keeping the search space bounded. The lower bound on the membership-function width, $\sigma_{min}=0.15,$ was selected after a sensitivity sweep over $\sigma_{min}\in \{0.10,0.15,0.20,0.25\}$; values of $\sigma_{min}$ below this range produced Gaussian membership functions narrow enough to create sharp, piecewise-linear transitions between adjacent rules, which manifested as overshoot and local oscillations in the closed-loop response even when the corresponding offline validation error was statistically indistinguishable from that of wider settings.

#### 2.3.7. Fitness Function

Every candidate vector x is decoded into a complete Sugeno fuzzy inference system in Equations (11)–(15) and evaluated on a held-out validation subset $D_{val}=\{\left(u_{n},y_{n}\right){\}}_{n=1}^{N_{val}}$, disjoint from the training and test subsets. The fitness assigned to x is the mean squared error between the fuzzy system’s predicted outputs and the corresponding targets, averaged over both controller outputs,

(39) $$J\left(x\right)=\frac{1}{2N_{val}}\sum_{n=1}^{N_{val}}\left[{\left(D_{p,n}\left(x\right)-\hat{D_{p,n}}\right)}^{2}+{\left(D_{m,n}\left(x\right)-\hat{D_{m,n}}\right)}^{2}\right],$$

where $D_{p,n}\left(x\right)$ and $D_{m,n}\left(x\right)$ are the fuzzy system’s outputs in Equation (15) for the n-th validation input pair, $\hat{D_{p,n}}$ and $\hat{D_{m,n}}$ are the corresponding target values from the MMPC-generated dataset and $N_{val}$ is the number of validation samples. Each optimization algorithm seeks $x^{*}=\arg\underset{x}{\min}\;J\left(x\right)$ subject to the bounds in Table 1. Using the validation subset, rather than the training subset, to score candidates mirrors the early-stopping principle used to train the ANN controller in [19] and discourages the optimizer from converging to parameter vectors that overfit the training data.

#### 2.3.8. Data Preprocessing

The dataset $\left\{\left(i_{s,n},i_{s,ref,n},D_{p,n},D_{m,n}\right)\right\}$, generated by the validated MMPC closed-loop simulation of [18] and reused for the ANN controller of [19], was normalized to the range $\left[-1,1\right]$ prior to optimization. For a generic variable v with known physical bounds $\left[v_{min},v_{max}\right]$, the normalized variable is

(40) $$\tilde{v}=2\;\frac{v-v_{min}}{v_{max}-v_{min}}-1,$$

where $\tilde{v}$ denotes the normalized value of v, and $v_{min},v_{max}$ are the corresponding physical bounds: the sample minima and maxima for the inputs $i_{s}$ and $i_{s,ref}$, and the physical actuator limits $D_{p}\in \left[0,147\right]$ and $D_{m}\in \left[68,160\right]$ for the outputs. This is the same min–max range used by the mapminmax input/output processing layers of the ANN controller in [19], so that the two data-driven controllers are trained and evaluated under an identical normalization convention. The de-normalization used to recover physical units from a fuzzy-system output $\tilde{v}$ is the inverse of Equation (40),

(41) $$v=\;\frac{\tilde{v}+1}{2}\left(v_{max}-v_{min}\right)+v_{min}.$$

The normalized dataset was partitioned into training, validation and test subsets using a 70%/15%/15% split (1261/270/270 samples) with a fixed random seed, matching the partition ratio used for the ANN controller in [19].

The three subsets play distinct roles. The training subset is not used by the fitness function of the metaheuristics: every candidate is scored only on the validation subset in Equation (39), which therefore performs model selection, and the test subset is used once, after tuning, to report the offline metrics of in Section 3.1; it never influences any design choice. Because the partition is made by random sampling from a simulated time series, samples adjacent in time can fall into different subsets, and the offline test metrics may therefore be optimistic. For this reason, the offline metrics are used only to compare the tuning methods under identical conditions. In contrast, the evaluation of the controller relies on the closed-loop simulations, which are independent of the partition: the stochastic scenario uses disturbance realizations absent from the data, and the extended scenario uses reference segments and disturbance events never seen during tuning.

## 3. Results

The tuned Sugeno fuzzy inference system was deployed in the same Simulink model, running in MATLAB/Simulink R2024b from The MathWorks Inc., Natick, MA, USA, and in the same processor-in-the-loop (PIL) configuration (BeagleBone Black target) used in [18,19], replacing the MMPC controller block with a fuzzy logic controller block referencing the tuned fuzzy structure. Gain-and-bias blocks were placed immediately before and after the fuzzy logic controller block to convert between physical and normalized units, and saturation blocks enforcing $D_{p}\in \left[0,147\right]$ and $D_{m}\in \left[68,160\right]$ were placed at the controller output. The closed-loop system was evaluated under the same deterministic and stochastic torque disturbance profiles used in [18,19], and its performance was quantified using the same metrics reported therein (maximum overshoot, peak time and closed-loop tracking root-mean squared error), enabling a direct comparison with the previously published MMPC results. The controller execution time on the target was measured with the PIL profiler.

The performance of each controller was quantified by the maximum overshoot (MO) and peak time ($t_{p})$ of the stroke ($i_{s}$) response, the root-mean-square tracking error $RMSE=\sqrt{\left(1/N_{s}\right)\sum_{k}{\left(i_{s,ref}\left(k\right)-i_{s}\left(k\right)\right)}^{2}}$, the integral error indices $IAE=\int \left|e\left(t\right)\right|\;dt$, $ISE=\int e{\left(t\right)}^{2}\;dt$, $ITAE=\int t\;\left|e\left(t\right)\right|\;dt$ and $ITSE=\int t\;e{\left(t\right)}^{2}\;dt$, with $e=i_{s,ref}-i_{s}$ integrated over the logged run, the average control effort (AE) and the average computational load (ACL). The AE used in [18,19] is defined as follows:

(42) $$AE\;=\frac{1}{2N_{s}}\sum_{k=1}^{N_{s}}\left[\frac{D_{p}\left(k\right)}{D_{p,max}}+\frac{D_{m}\left(k\right)-D_{m,min}}{D_{m,max}-D_{m,min}}\right]$$

i.e., the time average over the $N_{s}$ samples of the run of the mean of the two normalized displacement commands, where $D_{p,max}=147$ cm^3^/rev, $D_{m,min}=68$ cm^3^/rev and $D_{m,max}=160$ cm^3^/rev and ACL is the mean execution time of one controller step on the BeagleBone Black target, measured by the PIL profiler over the run. AE measures the magnitude of the actuator commands, not the hydraulic or mechanical energy consumed. All metrics of Tables 9, 11 and 14 were computed from responses logged at 0.1 s intervals.

### 3.1. ANFIS Tuning with AOO, HHO, PSO, GA and CMA-ES

All five population-based algorithms, namely AOO, HHO, PSO, GA and CMA-ES, were run 10 independent times with a population size of N=30 and T=100 iterations, using the identical fitness function and decision-vector encoding in Table 1. Table 2 reports the mean, standard deviation and best validation-set fitness achieved across the 10 runs, together with the held-out test-set root-mean-square error (RMSE) of the corresponding best-found ANFIS for each of the two controller outputs, $D_{p}$ and $D_{m}$, in physical units.

Among the five population-based optimizers, AOO attains the lowest mean validation fitness. Its advantage over HHO, GA and CMA-ES is statistically significant after Holm correction, whereas AOO and PSO are statistically indistinguishable according to the Wilcoxon rank-sum tests reported below; PSO reaches a slightly better single best run and a lower Dm test RMSE, AOO shows a slightly lower mean. CMA-ES shows the smallest run-to-run spread but converges to a higher error level within the budget of 3000 evaluations, which is small for adapting a full covariance matrix in 66 dimensions.

**Figure 2:**
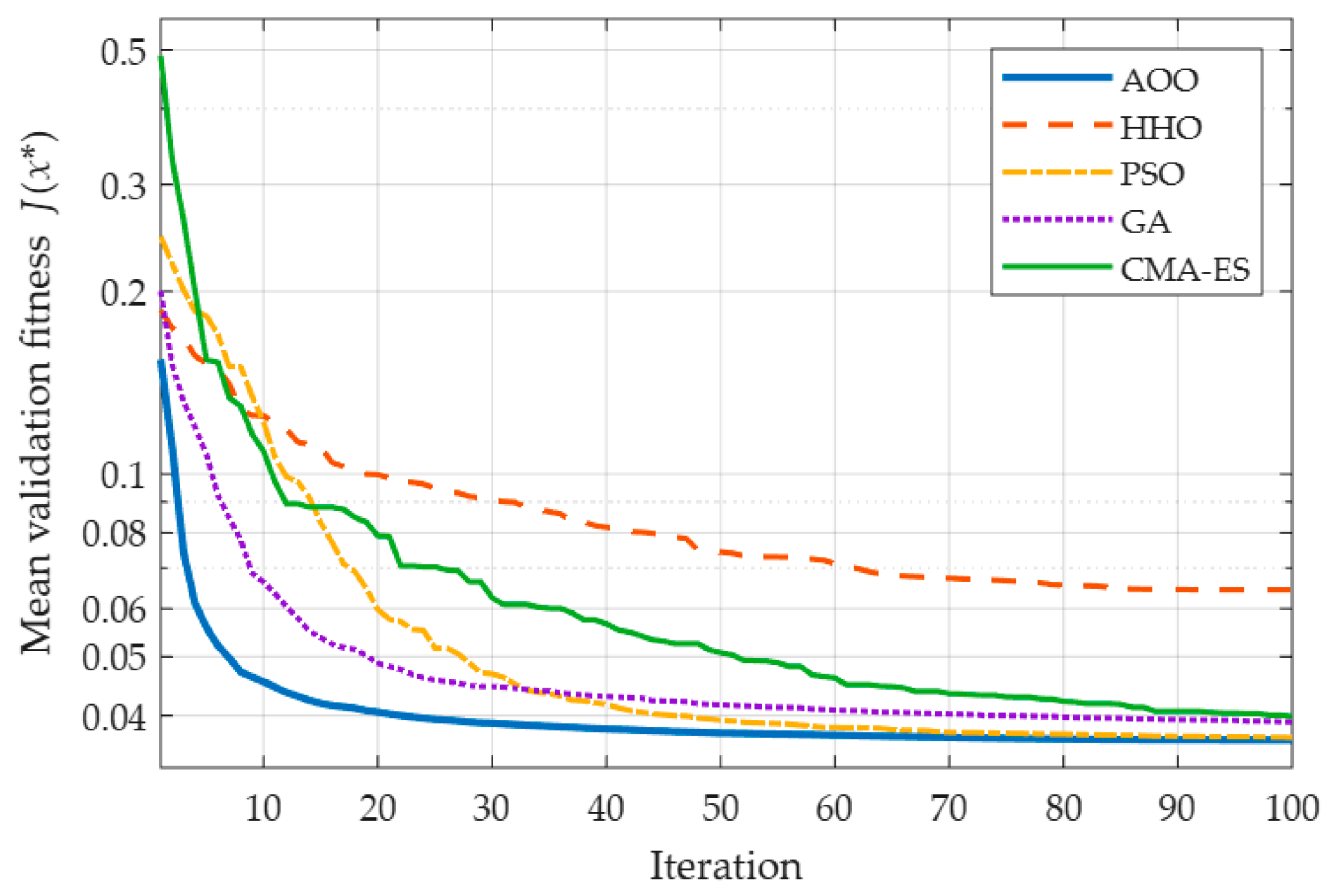
Mean validation-fitness convergence of the five population-based optimizers over 10 independent runs (N=30, T=100 iterations).

Figure 2 shows the mean validation-fitness trajectory of the five population-based optimizers over the 100 iterations, averaged across the 10 independent runs.

**Figure 3:**
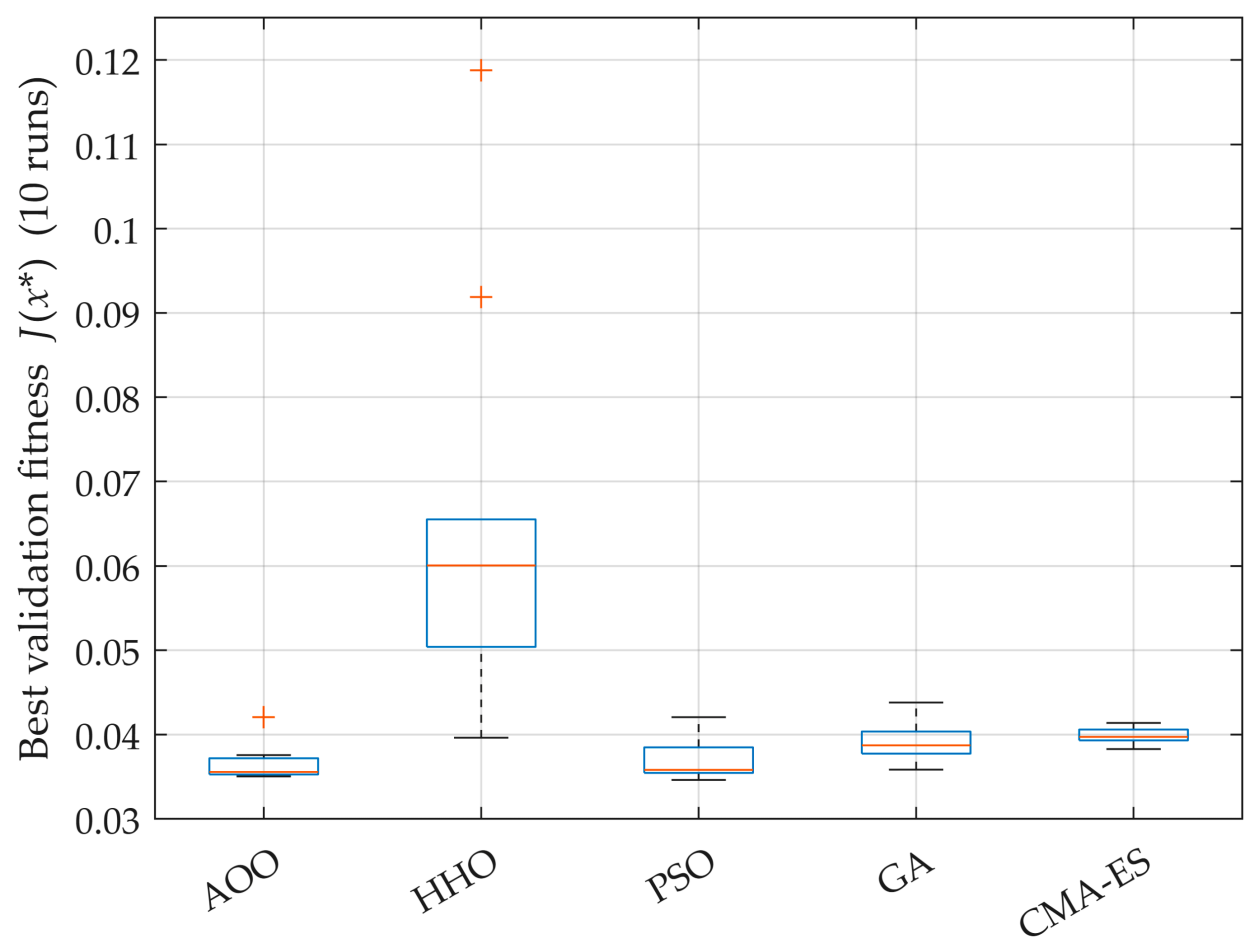
Distribution of best validation fitness $J\left(x^{*}\right)$ over 10 independent runs for each optimizer.

To assess whether the differences between the optimizers are statistically significant rather than a product of chance, two-sided Wilcoxon rank-sum tests [79] were applied to the 10 best-fitness values of AOO and of each alternative, with Holm–Bonferroni correction for the four comparisons. The Holm-corrected *p*-values are 9.85 × 10^−4^ (AOO versus HHO), 0.473 (AOO versus PSO), 1.46 × 10^−2^ (AOO versus GA) and 8.48 × 10^−3^ (AOO versus CMA-ES): AOO is significantly better than HHO, GA and CMA-ES and statistically tied with PSO. Figure 3 shows the corresponding distribution of best-fitness values as a box plot. Each box spans the interquartile range with the median shown as a red line; the whiskers extend to the most extreme runs within 1.5 times the interquartile range, and red crosses mark outlying runs.

To illustrate concretely what each algorithm identified as the optimal fuzzy-controller parameterization, Table 3 and Table 5 report the best-found membership-function and consequent parameters of the AOO-tuned ANFIS, and Table 4 and Table 6 those of the four baseline optimizers.

**Table 3:** Tuned Gaussian membership-function parameters with AOO.

MF Index	$i_{s}$ Center	$i_{s}$ σ	$i_{s,ref}$ Center	$i_{s,ref}$ σ
1	0.8456	0.3848	−0.4021	0.5078
2	0.1910	0.6458	0.3266	0.9904
3	0.8955	0.9580	−0.2961	0.5164

**Table 4:** Tuned Gaussian membership-function parameters with the four baseline optimizers HHO, PSO, GA and CMA-ES.

Optimizer, MF Index	$i_{s}$ Center	$i_{s}$ σ	$i_{s,ref}$ Center	$i_{s,ref}$ σ
HHO 1	0.7472	0.9975	−0.6662	0.9975
HHO 2	−0.1636	0.8136	−0.2427	0.7147
HHO 3	−0.3038	0.9975	0.0242	0.9307
PSO 1	0.9812	0.9376	−0.5393	0.9958
PSO 2	−0.1337	1.0000	−0.7560	0.6865
PSO 3	0.9588	0.6342	−0.3303	0.8244
GA 1	−0.7816	0.3052	0.1269	0.9456
GA 2	−0.9114	0.7766	−0.4423	0.4597
GA 3	−0.8660	0.8738	0.1508	0.5607
CMA-ES 1	−0.6856	1.0000	0.2377	0.8087
CMA-ES 2	0.1161	0.8755	0.6079	0.7843
CMA-ES 3	0.2659	0.9994	0.3710	0.5394

**Table 5:** Tuned Sugeno consequent coefficients with AOO.

Rule	$a_{D_{p}}$	$b_{D_{p}}$	$c_{D_{p}}$	$a_{D_{m}}$	$b_{D_{m}}$	$c_{D_{m}}$
1	−0.4293	−0.0043	0.5693	0.0277	0.4930	−0.9376
2	−2.8697	1.5466	1.3349	0.1088	−2.2529	−1.3170
3	−0.1626	−2.5168	−1.3693	−1.6891	−1.6225	1.1402
4	−1.4827	2.1933	0.8191	−1.4594	−1.5804	0.7485
5	0.5811	1.9960	0.5234	2.5041	−2.6729	−1.5484
6	−0.9372	−0.3273	1.9994	2.1223	−2.8092	1.4127
7	2.9867	2.7492	−0.1804	−1.0238	−0.6106	1.4911
8	−0.6742	1.7732	0.0020	0.3354	1.8266	0.3758
9	−1.0966	0.0115	1.2023	2.4091	1.8900	1.4105

**Table 6:** Tuned Sugeno consequent coefficients with the four baseline optimizers HHO, PSO, GA and CMA-ES.

Optimizer, Rule	$a_{D_{p}}$	$b_{D_{p}}$	$c_{D_{p}}$	$a_{D_{m}}$	$b_{D_{m}}$	$c_{D_{m}}$
HHO 1	−2.5165	1.3489	1.0095	1.1354	1.5136	−0.1458
HHO 2	−1.9207	−2.1112	1.6973	−1.1814	−2.8948	1.4757
HHO 3	−1.5398	2.5620	1.8885	−1.6567	0.0944	1.7294
HHO 4	−0.8461	2.6029	1.9234	1.5808	−1.9803	1.1090
HHO 5	0.4407	1.8986	−0.4710	1.1284	−0.7107	0.9448
HHO 6	2.8086	−1.0779	−0.5248	−2.5932	−1.0080	−1.9356
HHO 7	2.7035	−0.2044	0.3595	1.5799	−1.7262	0.3683
HHO 8	1.4832	0.4933	1.4345	−1.6374	−1.4668	−0.6912
HHO 9	−2.0404	−0.3604	0.0381	−2.6673	2.9295	0.6349
PSO 1	2.8329	0.4812	−0.5066	2.0812	−1.4481	1.3706
PSO 2	−0.3866	0.6798	1.7982	−2.9570	1.7053	−1.9018
PSO 3	1.3557	0.8567	1.6357	−1.3093	−3.0000	1.6256
PSO 4	2.1547	0.1051	1.1862	−0.6288	−2.1250	1.7222
PSO 5	−0.7340	2.6049	−0.8338	1.5576	2.7500	1.1021
PSO 6	−2.9159	1.1194	1.6862	−0.1862	−3.0000	−1.7024
PSO 7	−1.8036	−1.7278	−1.9391	3.0000	−1.4003	1.9855
PSO 8	0.3036	0.4712	1.5291	−2.8589	1.5475	−0.2270
PSO 9	3.0000	−3.0000	1.7637	−1.6994	−1.5641	−0.2551
GA 1	1.8906	2.8395	1.7185	−1.9406	0.0389	0.7725
GA 2	2.4921	−2.0146	−0.5093	1.2128	0.3594	−1.0208
GA 3	−0.3000	1.2447	0.7469	−1.9417	−1.6410	0.8661
GA 4	2.2931	−0.4511	1.3998	0.5125	−2.5632	1.9105
GA 5	−1.8339	−0.7238	0.5099	0.0228	−2.8783	−0.3099
GA 6	0.1236	−1.3345	0.7782	−2.3107	−2.9044	1.6894
GA 7	−1.7878	0.8176	0.4232	1.9658	−1.7781	−1.2489
GA 8	0.5488	2.1697	0.1735	−0.4065	2.4287	1.7804
GA 9	−0.7639	1.9805	1.1331	0.4851	1.3868	−0.5386
CMA-ES 1	−1.3822	1.1661	0.5822	1.4633	−0.6679	−0.0631
CMA-ES 2	0.6256	1.7196	1.2964	0.9264	−0.6881	−0.9430
CMA-ES 3	−0.1916	2.0686	1.5132	0.8416	−1.6975	1.7205
CMA-ES 4	0.7167	2.6478	1.0405	1.5801	−2.6586	1.3898
CMA-ES 5	−1.7914	1.0971	−0.7615	−1.3237	0.2738	−0.4500
CMA-ES 6	−1.8545	1.5406	0.4505	−1.1349	−0.2159	0.2873
CMA-ES 7	−1.3984	2.8461	−0.0820	−0.8887	−1.4960	1.3719
CMA-ES 8	−1.0849	−0.5864	1.6190	2.9042	−2.2032	−0.0537
CMA-ES 9	−0.1535	0.0915	0.8265	−2.0724	−1.1932	0.0053

**Figure 4:**
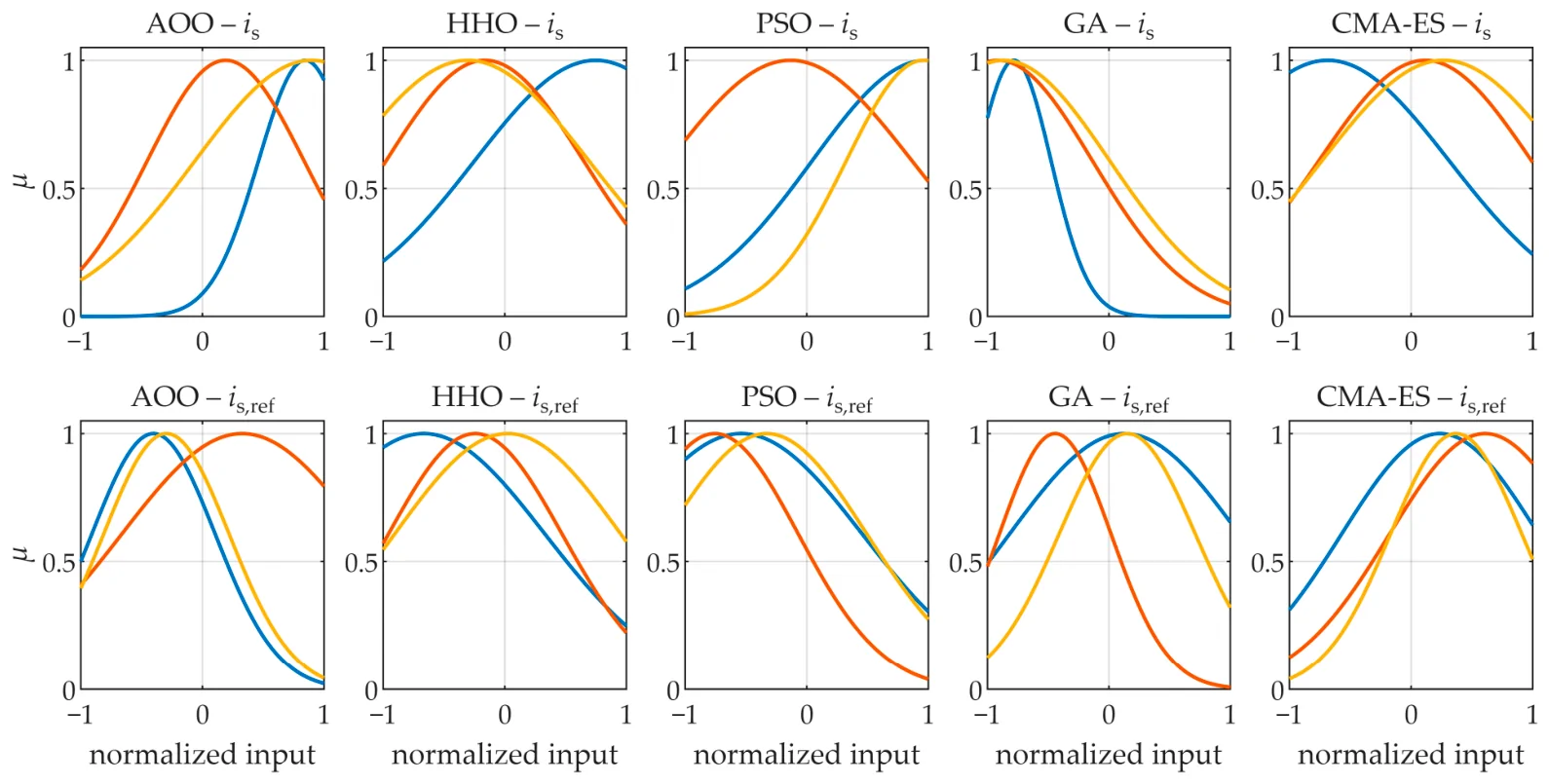
Tuned Gaussian membership functions with AOO, HHO, PSO, GA and CMA-ES for $i_{s}$ and $i_{s,ref}$.

Figure 4 plots the resulting Gaussian membership functions for the five optimizers, thereby making the numerical contrast in Table 3 and Table 4 directly visible. The blue, red and yellow curves correspond to membership functions 1, 2 and 3, respectively, as indexed in Table 3 and Table 4.

To complement the RMSE values in Table 2, Table 7 reports two further standard regression metrics for the best run of each of the five optimizerson the held-out test set, namely the mean absolute error (MAE) and the coefficient of determination ($R^{2}$), for both controller outputs.

The ranking mirrors that of Table 2; PSO and AOO attain the lowest absolute errors and the highest coefficients of determination on the held-out test set; GA follows closely, and CMA-ES and HHO trail. The differences between PSO and AOO are within the run-to-run spread reported in Table 2.

**Figure 5:**
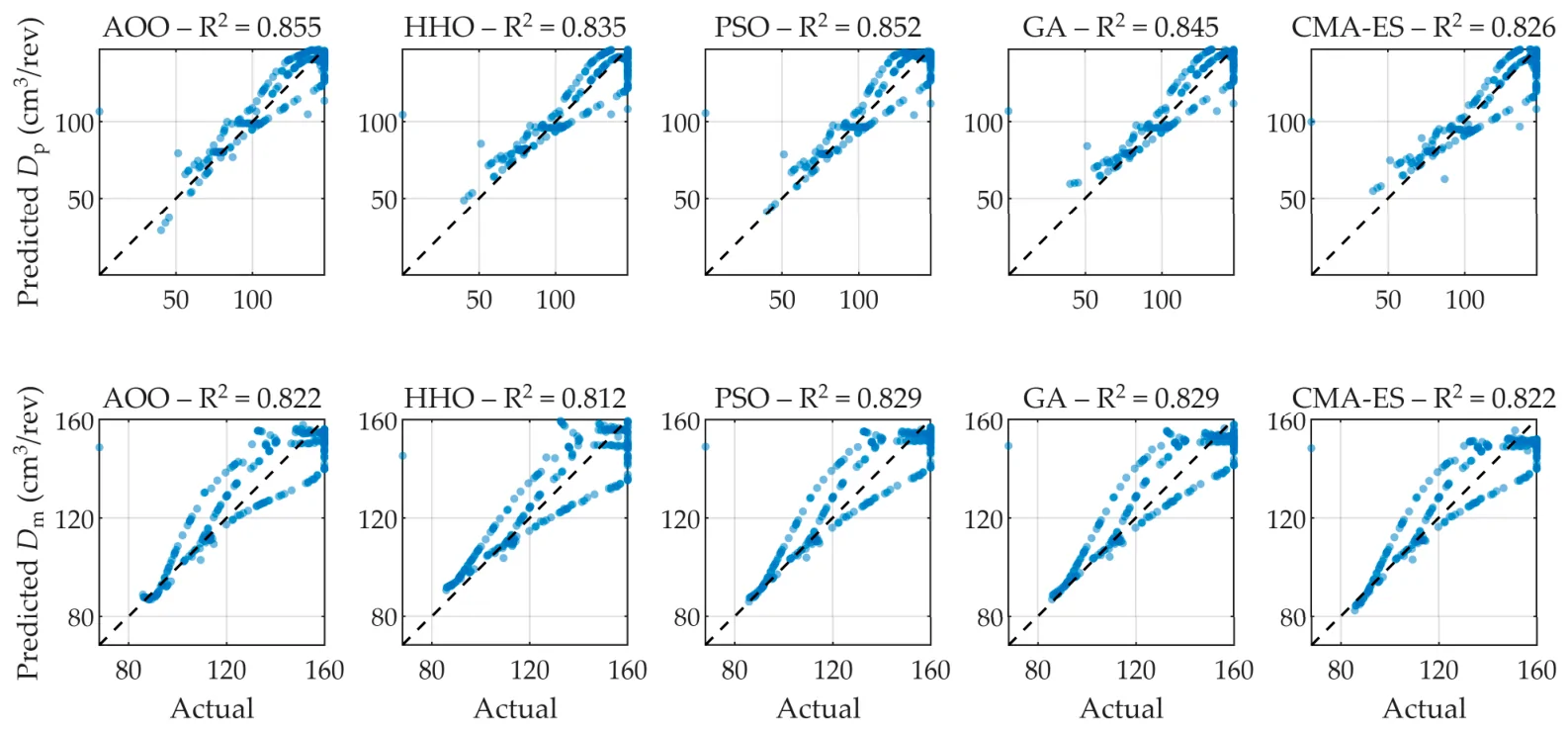
Predicted versus actual $D_{p}$ and $D_{m}$ on the held-out test set, for AOO, HHO, PSO, GA and CMA-ES.

Figure 5 shows the corresponding predicted-versus-actual scatter for both outputs and all five optimizers. The dashed line is the identity line.

### 3.2. Closed-Loop Processor-in-the-Loop (PIL) Simulation Performance of AOO-ANFIS and Comparison with MMPC and ANN

**Figure 6:**
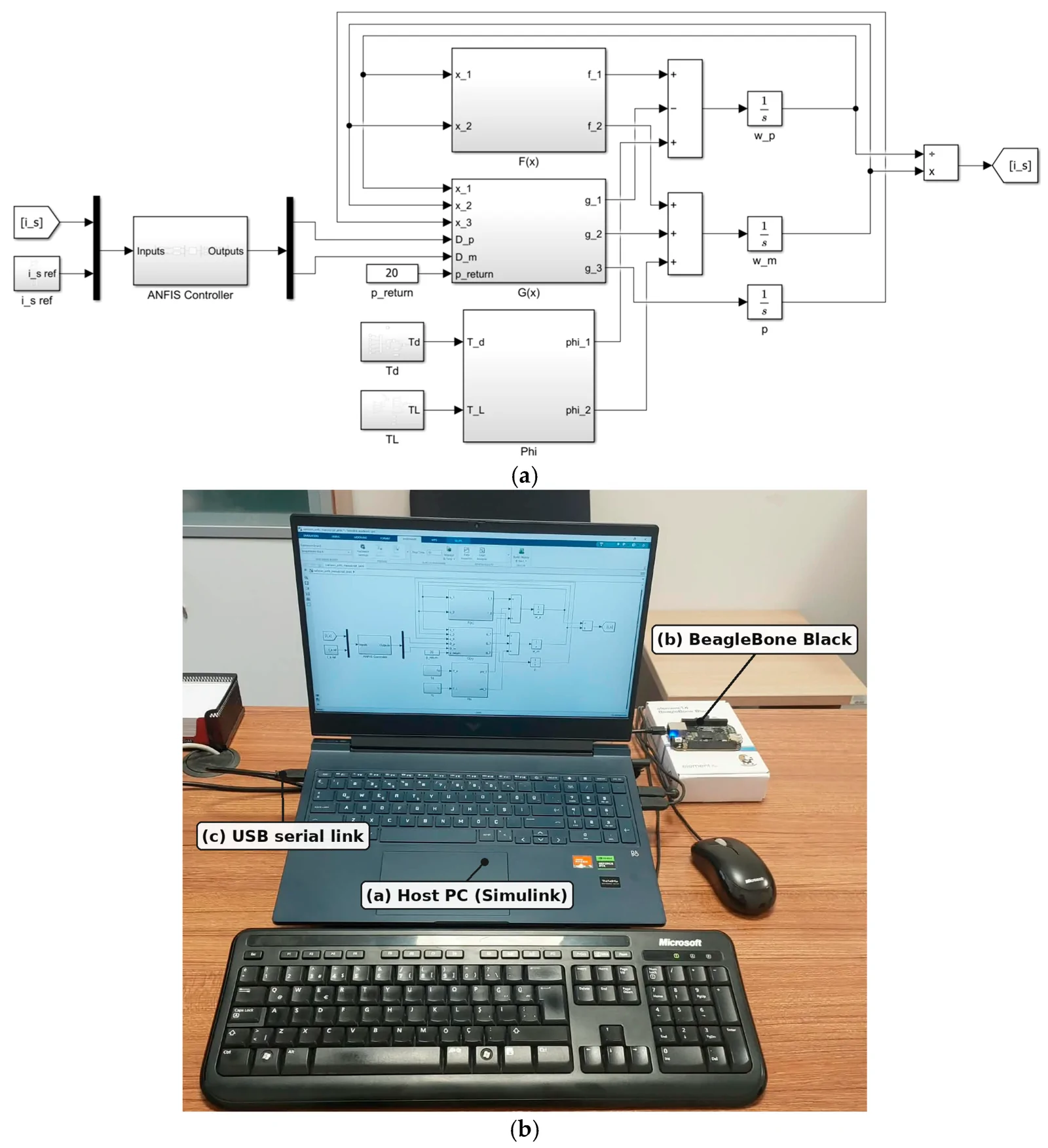
Closed-loop PIL configuration. (**a**) Block diagram of the closed-loop system with the ANFIS controller; (**b**) PIL test setup: host PC running the Simulink model, BeagleBone Black Rev C embedded target executing the controller object code, and USB serial communication link.

**Figure 7:**
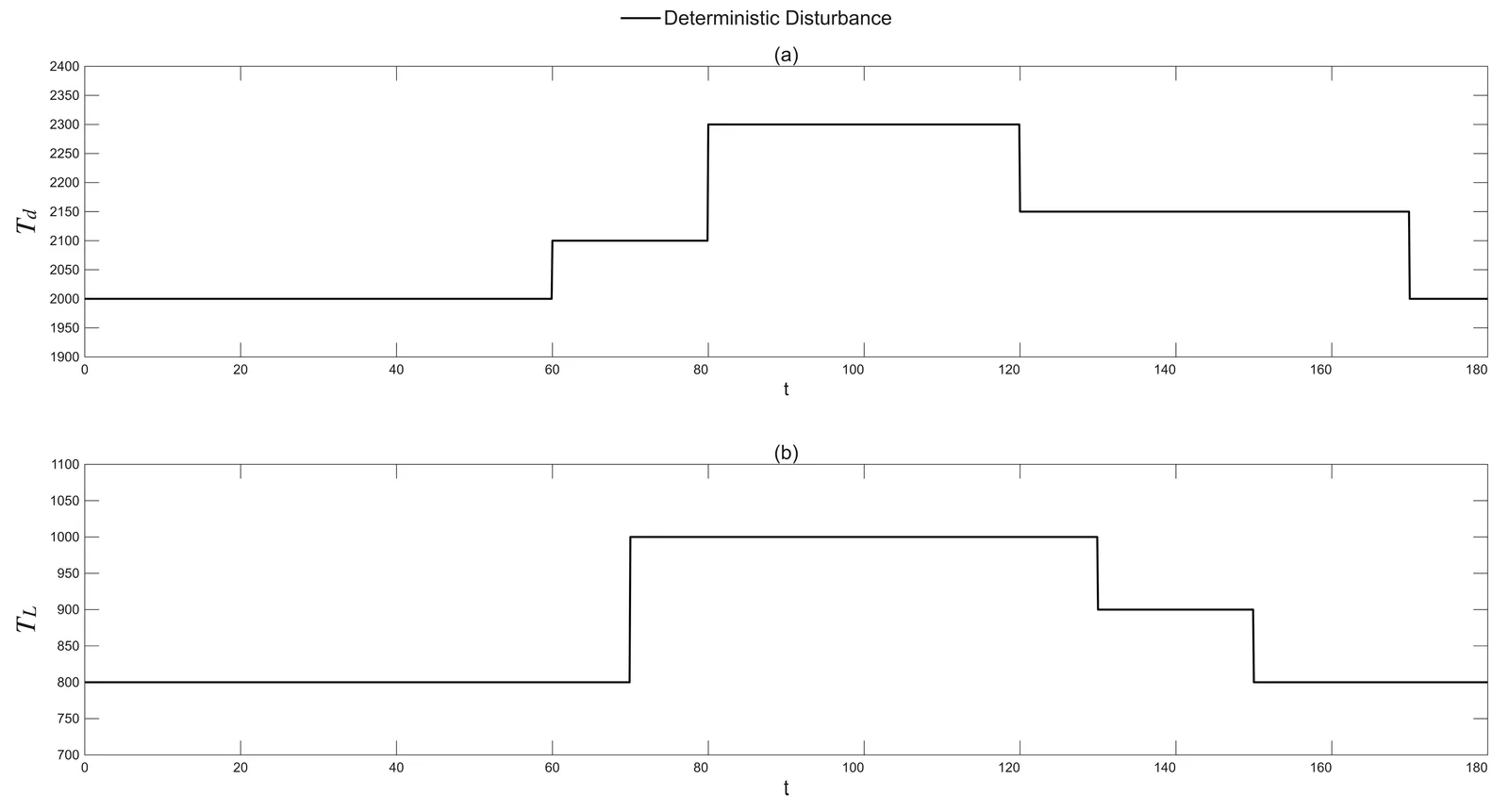
Deterministic torque disturbances: (**a**) $T_{d}$ and (**b**) $T_{L}$.

**Figure 8:**
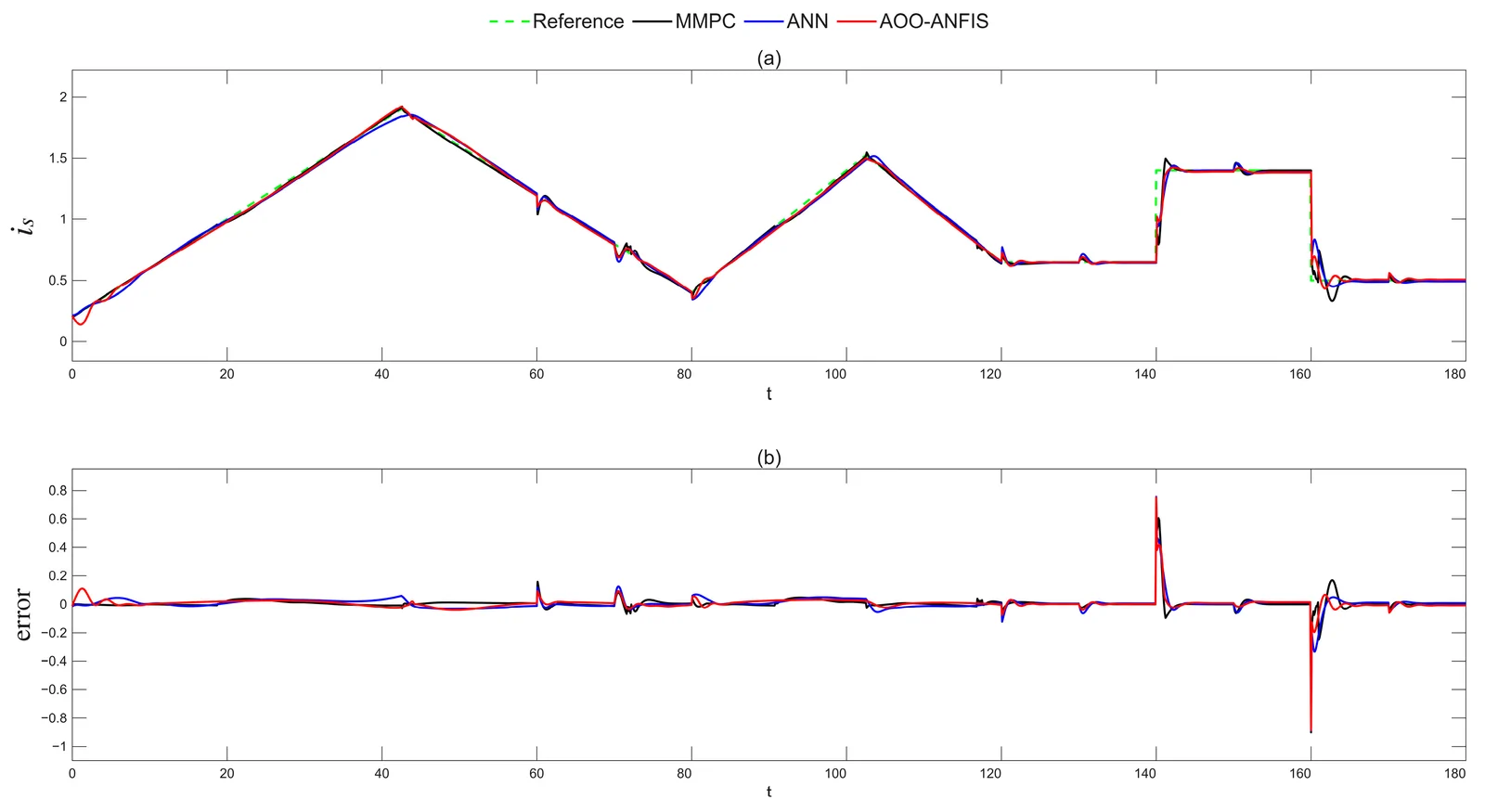
(**a**) Stroke responses and (**b**) errors of the closed-loop systems with MMPC, ANN and AOO-ANFIS in the case of deterministic torque disturbances.

**Figure 9:**
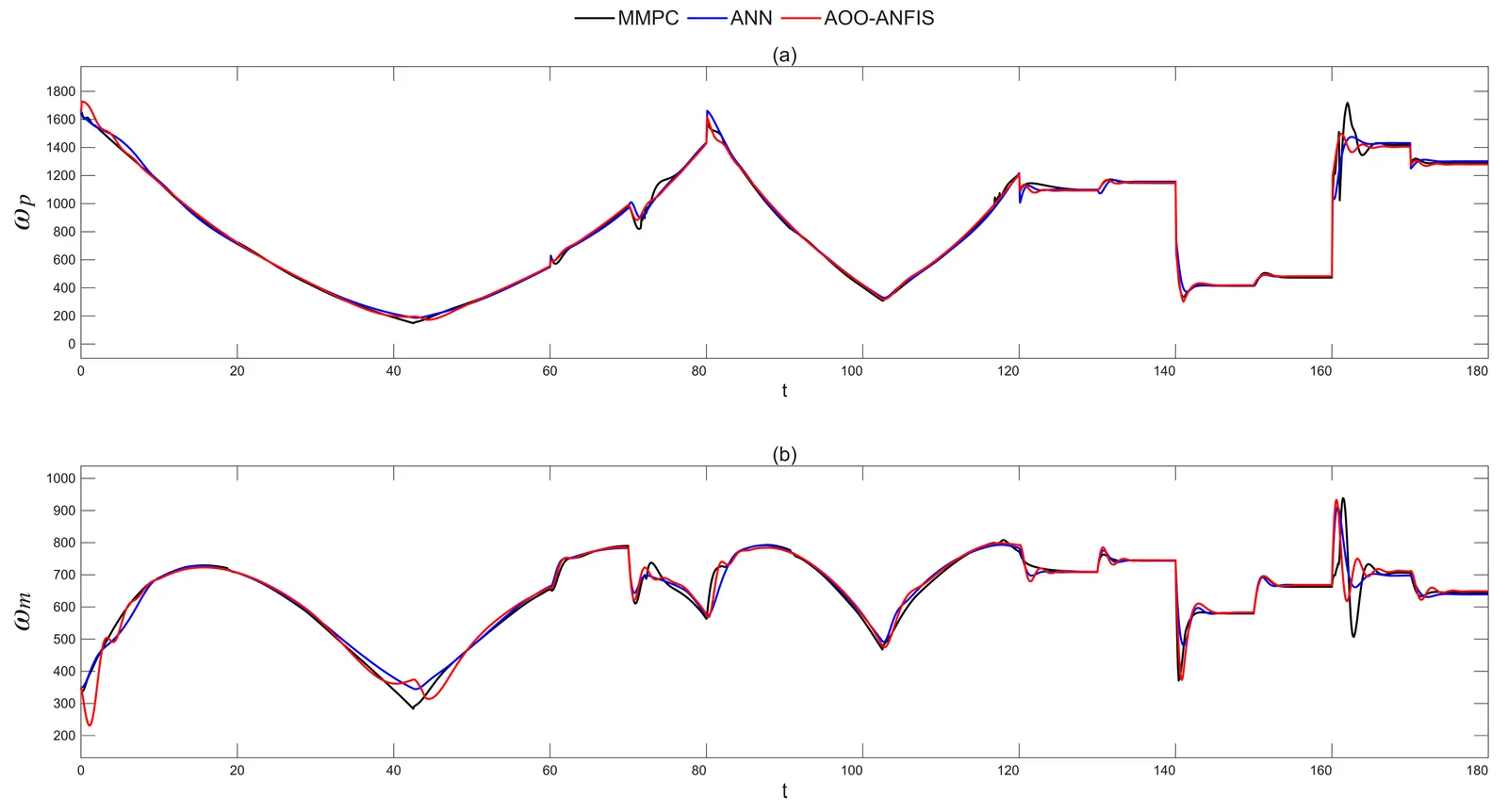
Angular speeds of the (**a**) pump and (**b**) motor of the closed-loop systems with MMPC, ANN and AOO-ANFIS in the case of deterministic torque disturbances.

**Figure 10:**
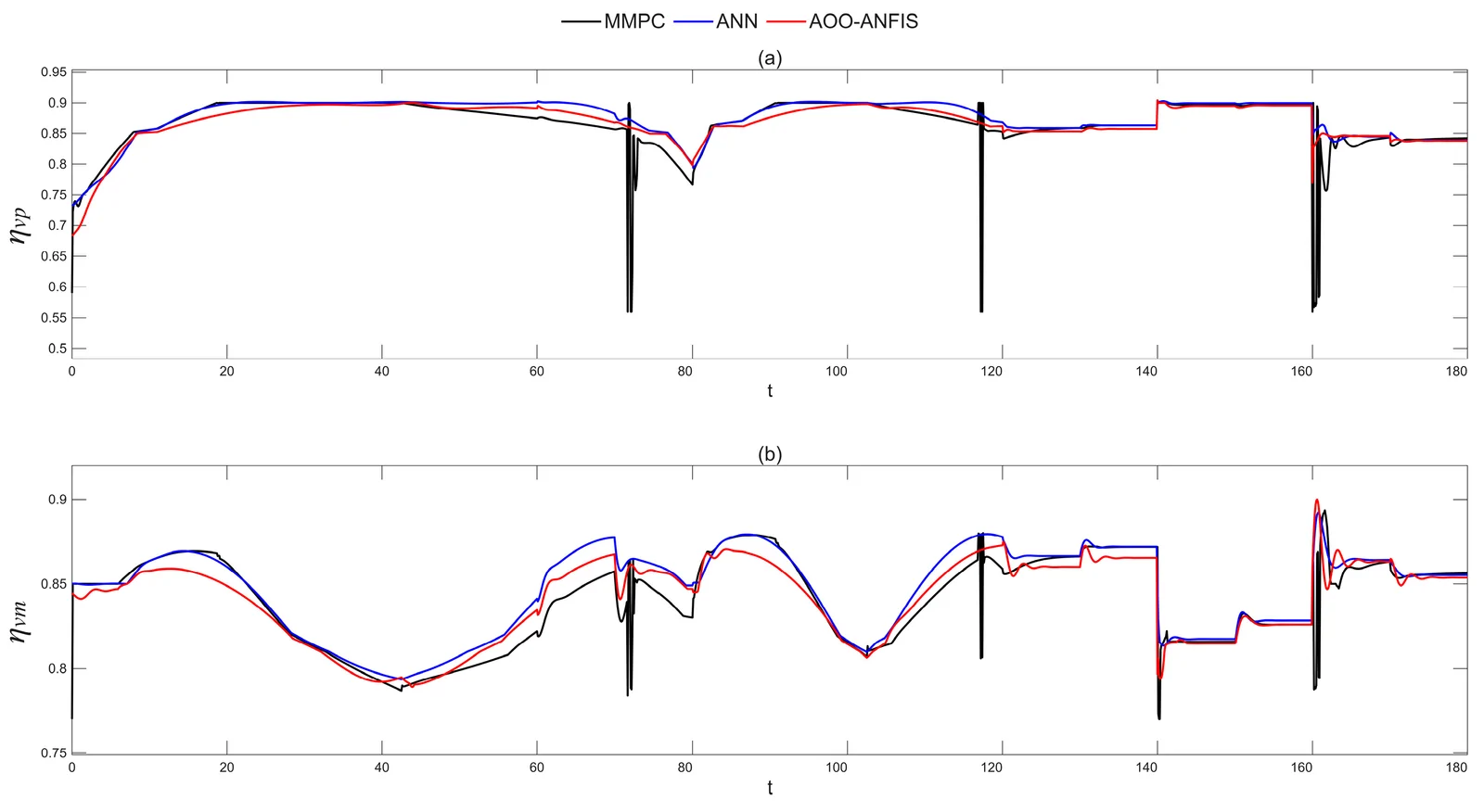
Volumetric efficiencies of the (**a**) pump and (**b**) motor of the closed-loop systems with MMPC, ANN and AOO-ANFIS in the case of deterministic torque disturbances.

**Figure 11:**
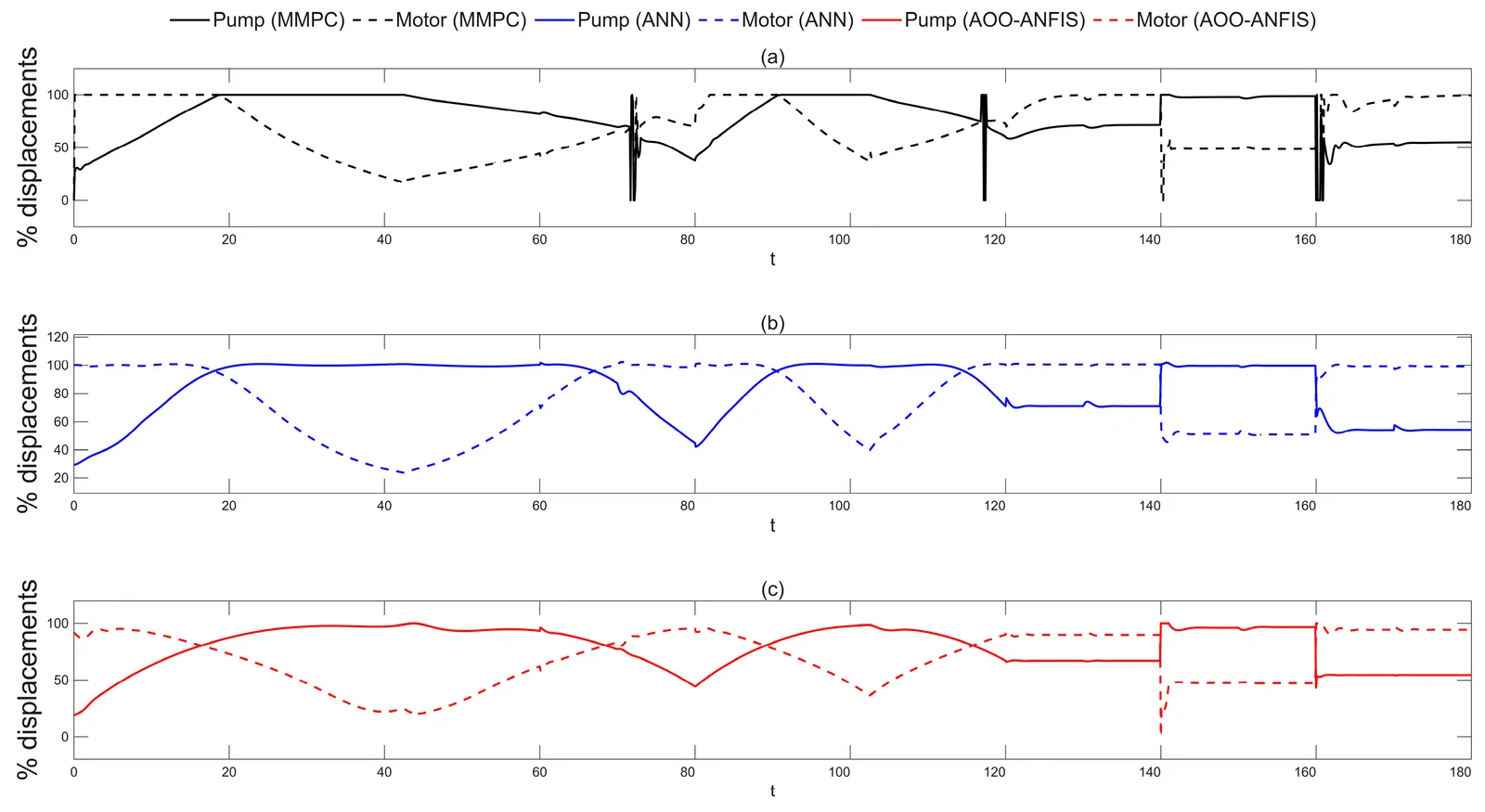
Normalized percentage of pump and motor displacements of the closed-loop systems with (**a**) MMPC, (**b**) ANN and (**c**) AOO-ANFIS in the case of deterministic torque disturbances.

**Figure 12:**
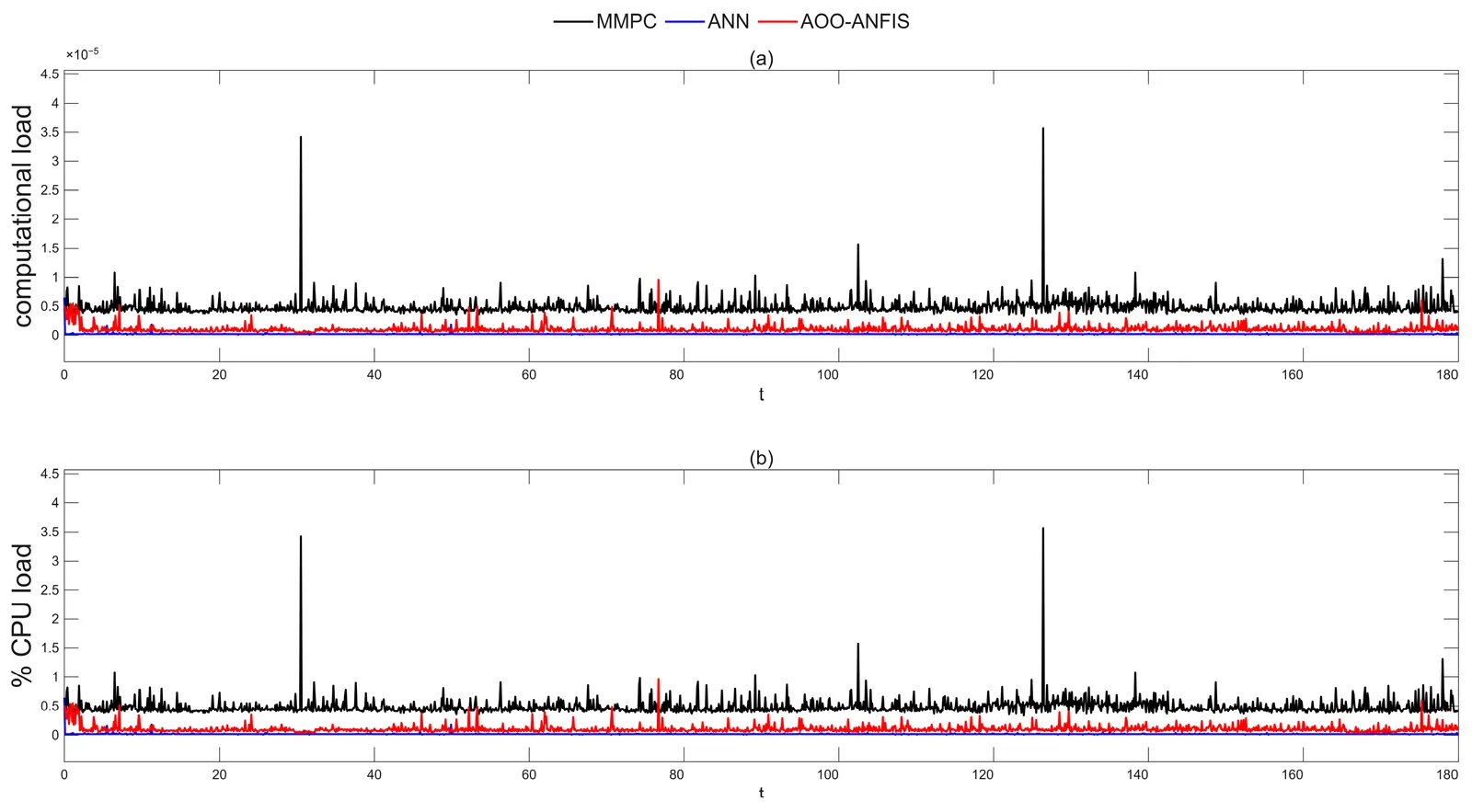
(**a**) Computational and (**b**) percentage CPU loads in the case of deterministic torque disturbances.

To begin with, the control efficacy of the AOO-ANFIS and MMPC methods was investigated on the closed-loop nonlinear time-varying HST representation shown in Figure 6 subject to deterministic disturbances and demanding trajectory targets. The results of the ANN surrogate of [19], obtained on the same scenario and the same target, are included in the tables of this section as a second reference. The benchmark goal was the accurate execution of primary and secondary control tasks. The deterministic disturbance profiles in Figure 7 comprised step and ramp transitions: $T_{d}$ increased from 2000 to 2300 Nm, $T_{L}$ stepped up from 800 to 1000 Nm and the $i_{s}$ setpoint varied from 0.2 to 1.9. All test cases were simulated over 180 s within the MATLAB R2024b environment utilizing a specialized stiff differential equation solver [18,19]. Detailed performance metrics, including stroke tracking errors ($i_{s,ref}-i_{s}$), processor load demands and percentage CPU utilization during PIL runs, are presented across Figure 8, Figure 9, Figure 10, Figure 11 and Figure 12.

**Figure 13:**
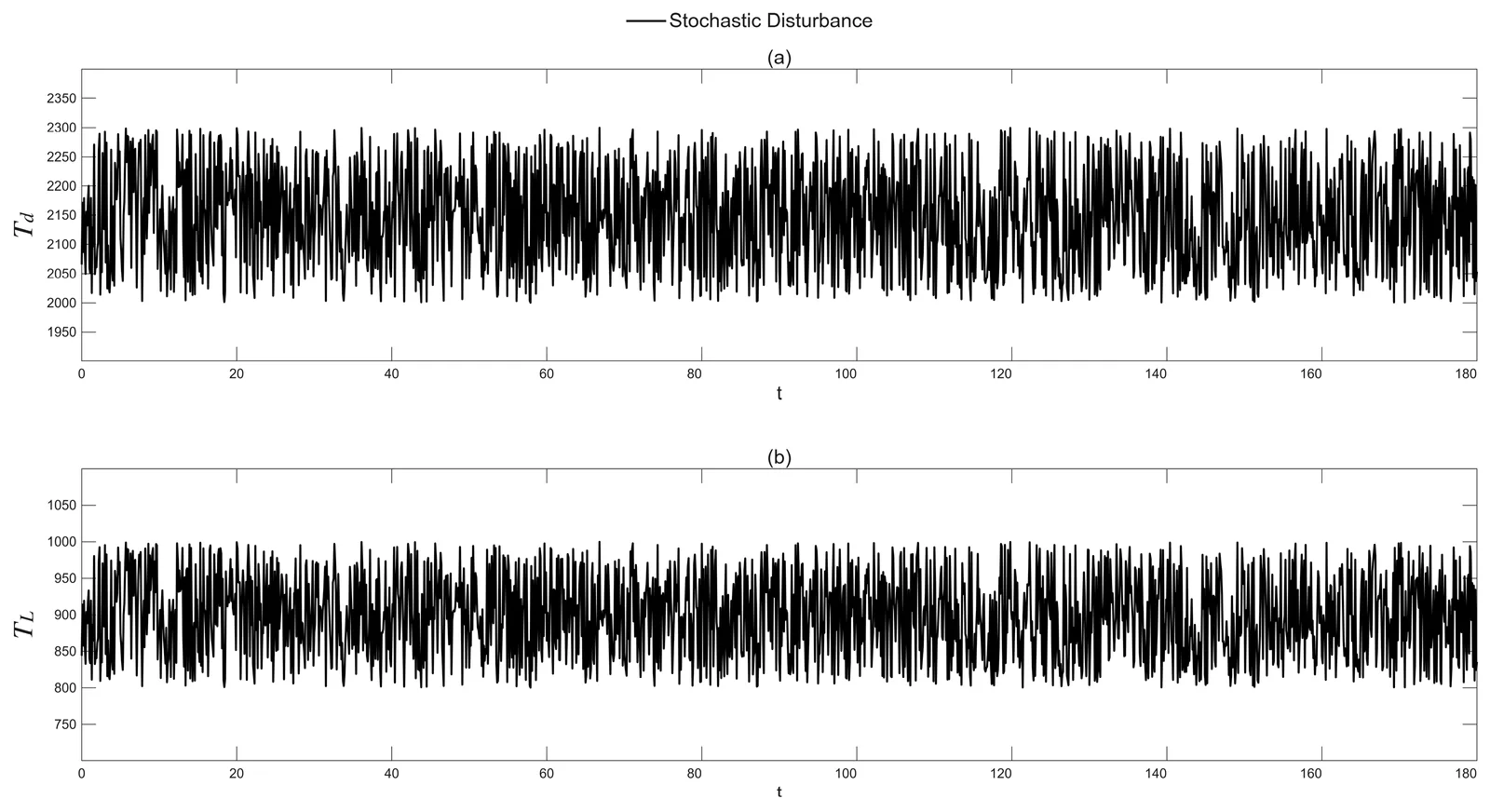
Stochastic torque disturbances: (**a**) $T_{d}$ and (**b**) $T_{L}$.

**Figure 14:**
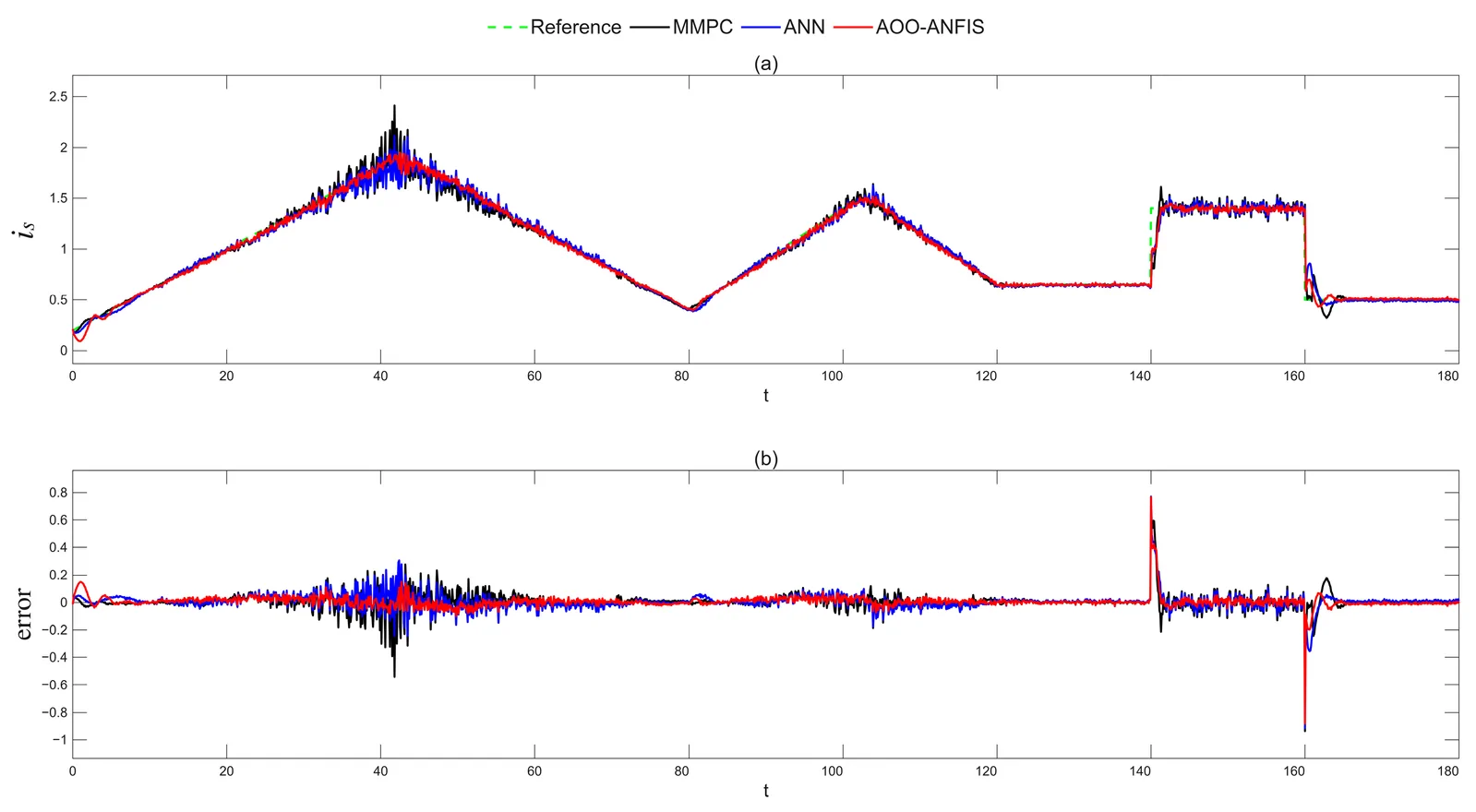
(**a**) Stroke responses and (**b**) errors of the closed-loop systems with MMPC, ANN and AOO-ANFIS in the case of stochastic torque disturbances.

**Figure 15:**
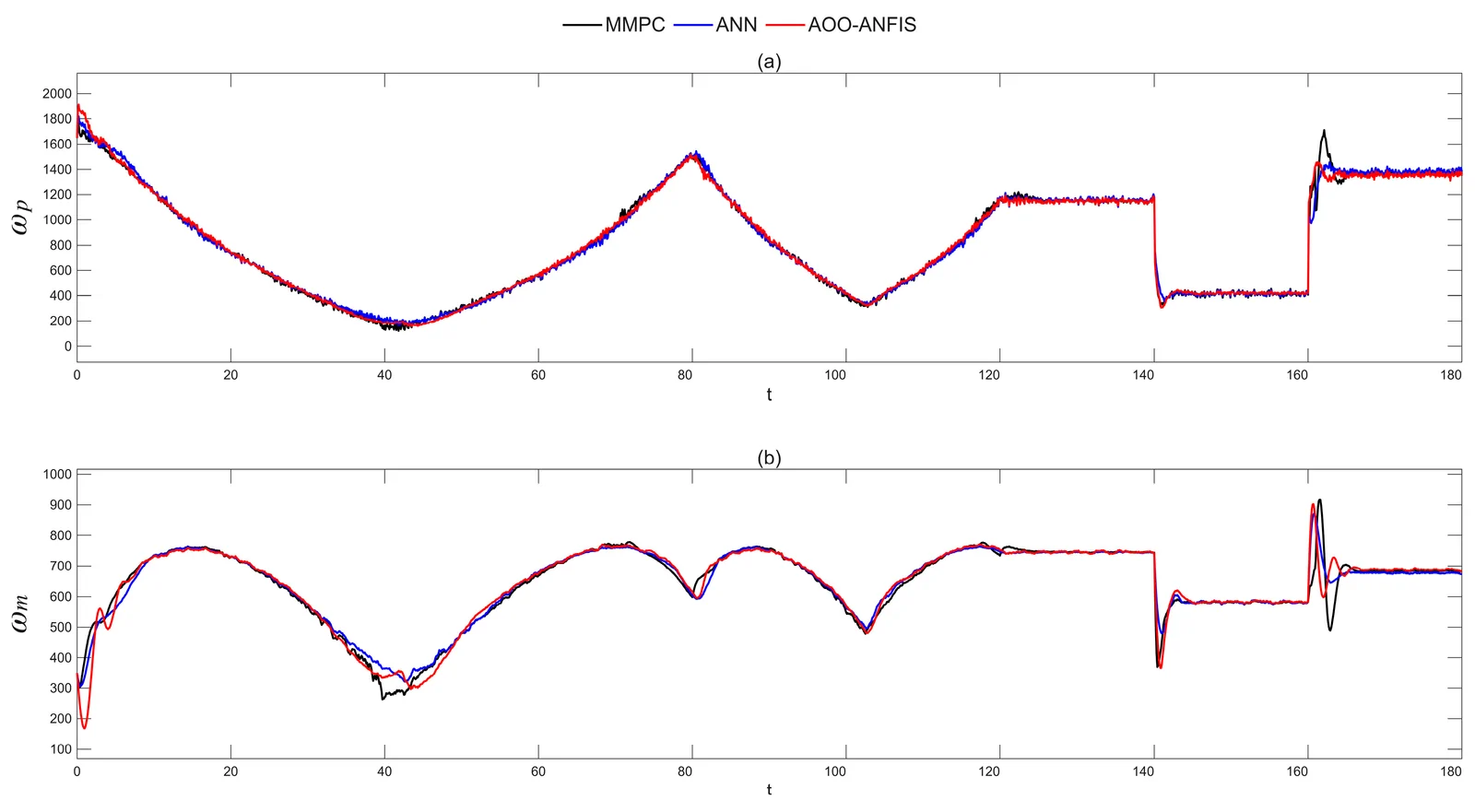
Angular speeds of the (**a**) pump and (**b**) motor of the closed-loop systems with MMPC, ANN and AOO-ANFIS in the case of stochastic torque disturbances.

**Figure 16:**
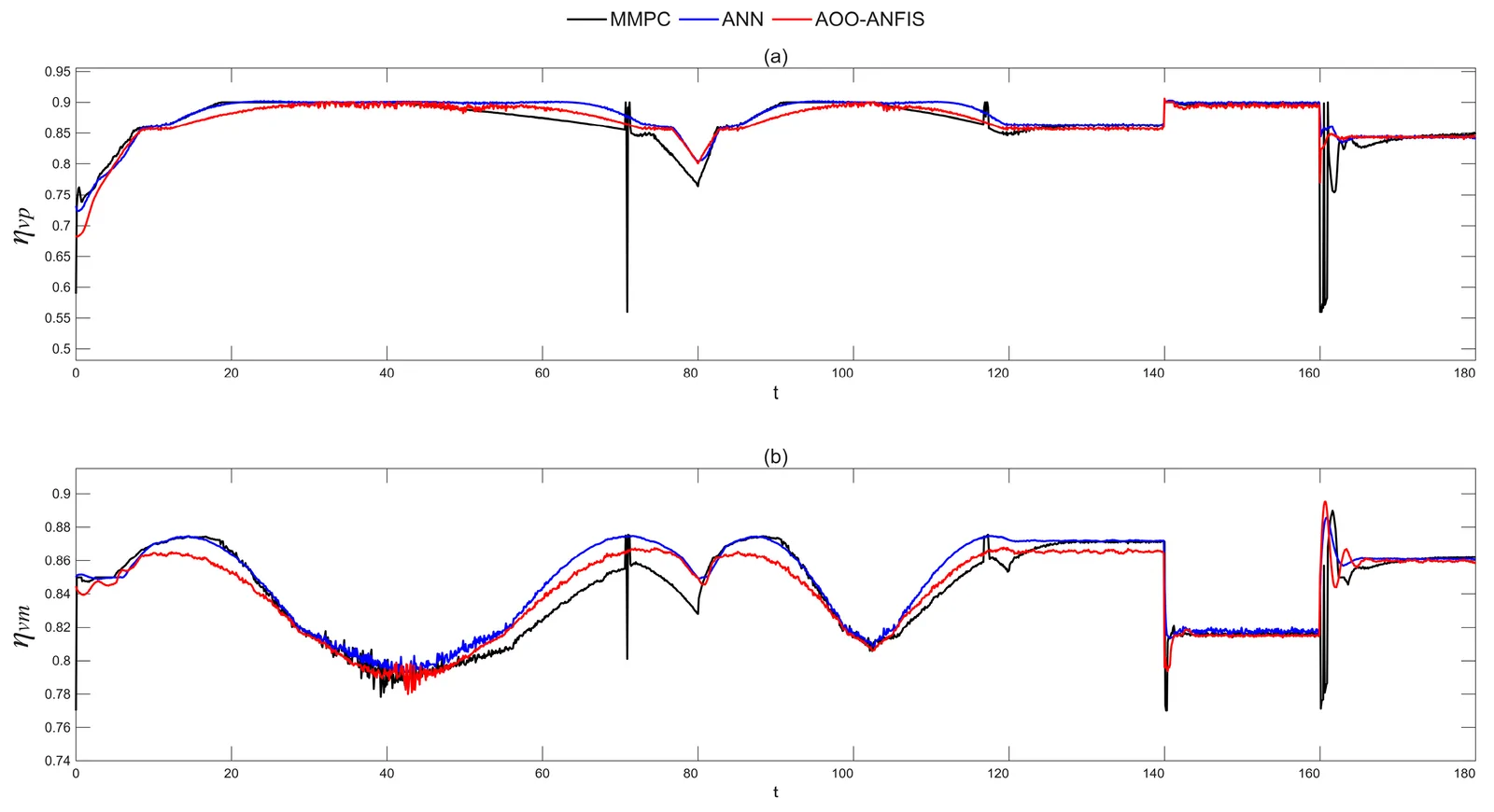
Volumetric efficiencies of the (**a**) pump and (**b**) motor of the closed-loop systems with MMPC, ANN and AOO-ANFIS in the case of stochastic torque disturbances.

**Figure 17:**
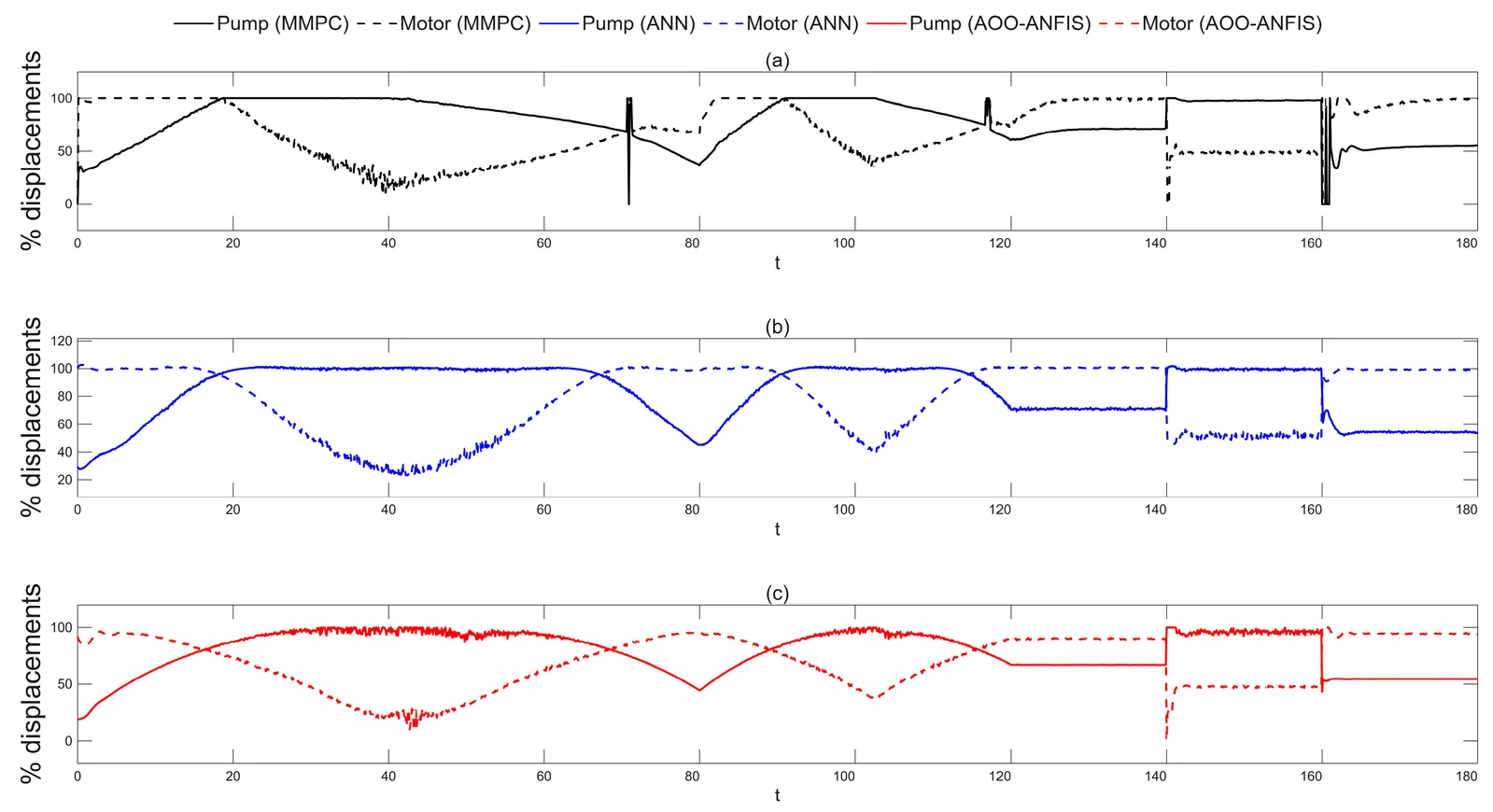
Normalized percentage of pump and motor displacements of the closed-loop systems with (**a**) MMPC, (**b**) ANN and (**c**) AOO-ANFIS in the case of stochastic torque disturbances.

**Figure 18:**
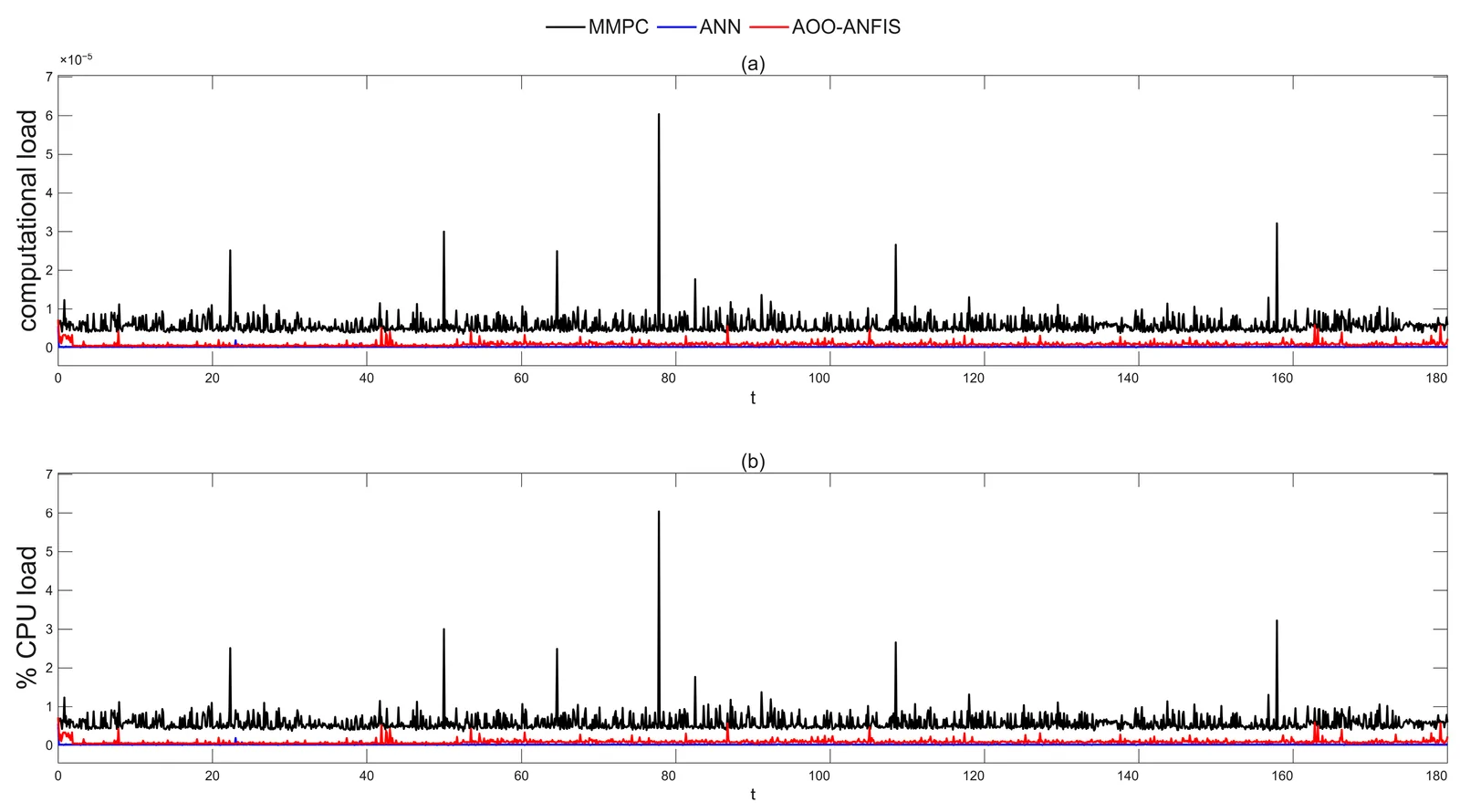
(**a**) Computational and (**b**) percentage CPU loads in the case of stochastic torque disturbances.

In the final evaluation stage, the control performance of the AOO-ANFIS and MMPC schemes was examined using the initial trajectory profile under stochastic operating conditions. These random disturbances, depicted in Figure 13, were implemented at 1 kHz using uniformly distributed noise within 2000–2300 Nm for $T_{d}$ and 800–1000 Nm for $T_{L}$. Numerical simulations of the closed-loop nonlinear models were carried out for 180 s within the MATLAB R2024b environment employing a stiff ODE solver [18,19]. Detailed performance indicators from the PIL tests, encompassing the error metrics between reference $i_{s,ref}$ and output $i_{s}$ alongside processor load requirements, are provided in Figure 14, Figure 15, Figure 16, Figure 17 and Figure 18.

The maximum overshoot (MO), peak time ($t_{p}$) and RMSE values for the PIL simulations under both deterministic and stochastic disturbances, as shown in Figure 8 and Figure 14, are presented in Table 8 and Table 9, together with the values of the ANN surrogate of [19] on the same scenario.

Both data-driven surrogates reduce the overshoot of the MMPC. Under deterministic disturbances, the ANN of [19] achieves the lowest overshoot, while the AOO-ANFIS achieves the lowest RMSE and reaches its peak slightly earlier. Under stochastic disturbances, the AOO-ANFIS achieves a 23% lower RMSE than the ANN and a considerably shorter peak time of 1.8 s versus 2.8 s, whereas the ANN retains a lower overshoot.

The error indices are given in Table 9 for the three controllers.

Under the stochastic disturbances, the AOO-ANFIS attains the lowest values among the four indices, with IAE, ISE, ITAE and ITSE, respectively, 34%, 51%, 34% and 41% below those of the MMPC and 31%, 40%, 32% and 35% below those of the ANN. Under deterministic disturbances, the AOO-ANFIS has the lowest squared-error indices in terms of ISE 15% and ITSE 21% below the MMPC. In contrast, the MMPC has the lowest absolute-error indices, with IAE at 19% and ITAE at 7%, below the AOO-ANFIS. The two families of indices weight the error differently: IAE and ITAE penalize the small but sustained tracking lag that the smoother surrogate exhibits along the ramps, while the short, large transients dominate ISE and ITSE that the MMPC produces at the reference steps and at its switching instants. The ANN of [19] lies between the two under stochastic disturbances and has the highest absolute-error indices under deterministic disturbances.

The percentage average effort (AE) and average computational load (ACL) in terms of the metrics for controller effort were obtained from PIL simulations for both deterministic and stochastic disturbances and are given in Table 10.

Both surrogates substantially reduce the computational load of the MMPC, by about 96% for the ANN and 76–84% for the AOO-ANFIS. The ANN, whose online evaluation reduces to a few small matrix–vector products, has the lowest execution time per step, about four to six times lower than the AOO-ANFIS. All three controllers execute two to four orders of magnitude faster than the 1 ms sample time, so the differences between the two surrogates are not decisive for this application. The AOO-ANFIS uses the lowest average control effort of the three controllers.

### 3.3. Generalization to an Extended Scenario Outside the Training Data

The training data of the surrogate were generated under the 180 s reference trajectory of [18] in Figure 8. To examine the controller outside this envelope, the scenario was extended to 300 s. The first 180 s reproduce the original trajectory exactly, so that the results of Section 3.1 and Section 3.2 can be verified within the same run; the remaining 120 s contain four classes of unseen excitation.

For the unseen reference levels and rates, a ramp from 0.5 to 0.3 (180–190 s), a steep ramp from 0.3 to 1.7 (190–200 s, 0.14 s^−1^, about 2.8 times the fastest ramp of the training data, 0.05 s^−1^) and a ramp down to 1.0 (200–210 s), followed by a plateau at 1.0 (210–240 s), a level that is only crossed, never held, in the training data.

For the unseen signal type, a sinusoidal reference 1.0 + 0.6 sin(2π(t − 240)/20) for 240–300 s (period 20 s, range 0.4–1.6, maximum rate 0.19 s^−1^, about 3.8 times the fastest training ramp); no periodic reference exists in the training data.

For the unseen disturbance type, trapezoidal torque disturbances instead of the steps used in training—the load torque $T_{L}$ rises linearly from 800 to 1000 Nm during 220–230 s, is held until 240 s and returns linearly to 800 Nm by 250 s; the drive torque $T_{d}$ rises from 2000 to 2300 Nm during 260–270 s, is held until 280 s and returns by 290 s.

For the stochastic variant, the same reference with the uniform stochastic torque noise of Section 3.2 was applied over the full 300 s. The ANN of [19], unchanged, was run on the same scenario for comparison. All runs are PIL simulations on the BeagleBone Black target. A 20 s period with a range of 0.4–1.6 was chosen for the sinusoid.

Figure 19, Figure 20, Figure 21, Figure 22, Figure 23 and Figure 24 and Figure 25, Figure 26, Figure 27, Figure 28, Figure 29 and Figure 30 show the two surrogates under the deterministic and stochastic variants.

It was not necessary to obtain MO and $t_{p}$ values of the PIL simulations for the extended scenario as the closed-system response up to 180 s was identical to that of previous simulations, as expected.

Table 11 gives the tracking metrics over the unseen segment (180–300 s) and the complete run. There was no need to calculate the tracking metrics over the trained segment (0–180 s) of the PIL simulations for the extended scenario, since the closed-system response up to 180 s was identical to that of previous simulations, as expected.

Both surrogates follow the unseen segment under both disturbance profiles; neither loses stability or drifts. Under deterministic disturbances the AOO-ANFIS attains the lower RMSE on the unseen segment as 0.0345 against 0.0557 for the ANN and the smaller maximum deviation there, 0.14 against 0.17; its error over the unseen segment is in fact lower than over the trained segment as 0.0447, which shows that the controller has learned the control law rather than the trajectory. The ANN tracks the steep ramp and the sinusoid with a slightly larger lag and shows a small steady offset about 0.02 on the 1.0 plateau, a level that is only crossed in the training data. Under stochastic disturbances, both surrogates remain bounded over the whole 300 s in unseen-segment RMSE 0.0406 for the AOO-ANFIS and 0.0616 for the ANN; the noise band of the AOO-ANFIS response is visibly narrower, in particular at the high stroke levels of the 1.7 peak and the sinusoidal maxima, which is consistent with the 23% lower RMSE of the AOO-ANFIS under stochastic disturbances on the design scenario in Table 9. The first 180 s of every run reproduce the metrics of Section 3.1 and Section 3.2 as AOO-ANFIS 0.0447 and 0.0485; ANN 0.0513 and 0.0627.

The AE and ACL values were obtained from the PIL simulations for the extended scenario and are given in Table 12.

Compared with the 180 s runs of Table 10, the control effort of both surrogates rises by one to two percentage points, to 79% for the ANN and 70% for the AOO-ANFIS, because the unseen segment holds the stroke at higher levels, up to 1.7 and 1.6, which require larger displacement commands; the nine-point advantage of the AOO-ANFIS is preserved. The execution time per step of the ANN is unchanged at about 2.2 × 10^−7^ s. That of the AOO-ANFIS, 4.8 × 10^−7^ and 5.7 × 10^−7^ s, is lower than in the 180 s runs although the controller and its code are identical; since the evaluation of nine Gaussian rules does not depend on the input values, this spread reflects the variability in the execution-time measurement on the embedded target rather than a property of the controller. In every run, the AOO-ANFIS remains two to three times slower than the ANN and more than three orders of magnitude faster than the 1 ms sample time.

### 3.4. Sensitivity to Volumetric-Efficiency Degradation

Throughout both disturbance scenarios, the pump and motor volumetric efficiencies vary with operating point according to their two-dimensional maps [8]; under the AOO-ANFIS controller, $\eta_{vp}$ ranges approximately from 0.80 to 0.90 and $\eta_{vm}$ from 0.79 to 0.90 after the initial transient in Figure 10 and Figure 16, i.e., the effective volumetric gains of the plant change by about 11–12% within a single run. This variation is part of the nominal plant model. To assess sensitivity to deviations from the nominal plant, the outputs of both volumetric-efficiency maps were additionally reduced to 85%, i.e., a simultaneous loss of approximately 15% in pump and motor volumetric efficiency, representing wear. Positive deviations were not considered because the nominal maps already reach 0.93 (pump) and 0.97 (motor), so that a comparable increase would yield physically impossible efficiencies above unity. The degraded plant was controlled by the ANN of [19] and the AOO-ANFIS, both with their nominal parameters, over the complete extended 300 s scenario of Section 3.3 under both disturbance profiles, so that the degradation is superimposed on the unseen reference segment and the unseen disturbance events.

Figure 31, Figure 32, Figure 33, Figure 34 and Figure 35 and Figure 36, Figure 37, Figure 38, Figure 39 and Figure 40 show the two surrogates under the deterministic and stochastic variants.

The MO and $t_{p}$ values for the PIL simulations for extended with degradation scenario under both deterministic and stochastic disturbances are presented in Table 13.

The reduced volumetric efficiencies lower the effective gain of the plant, so the initial reference step is reached more slowly by both controllers: the peak time of the ANN grows from 1 s to 3.2 s under deterministic and from 2.8 s to 3.0 s under stochastic disturbances, that of the AOO-ANFIS from 0.9 s to 2.1 s and from 1.8 s to 2.2 s. The overshoot of the ANN is unchanged at 10%, whereas that of the AOO-ANFIS increases from 14% to 16%. The two surrogates therefore keep their respective characters under degradation, the ANN with the lower overshoot and the AOO-ANFIS with the shorter peak time, and the peak time of the AOO-ANFIS on the degraded plant is still shorter than that of the ANN on the nominal plant under stochastic disturbances.

Table 14 gives the tracking metrics over the trained segment (0–180 s), the unseen segment (180–300 s) and the complete run with degradation.

Both surrogates retain stable, bounded tracking with the degraded system. The RMSE of the AOO-ANFIS over the full 300 s increases from 0.0409 to 0.0516 under deterministic disturbance and from 0.0456 to 0.0608 under stochastic disturbance; that of the ANN increases from 0.0531 to 0.0602 and from 0.0623 to 0.0685. The ANN is thus relatively less sensitive to the degradation as +13% and +10% versus +26% and +33%, whereas the AOO-ANFIS keeps the lower absolute error in both cases; on the unseen segment, the margin of the AOO-ANFIS over the ANN is preserved as 0.0433 against 0.0656 under deterministic disturbance, 0.0485 against 0.0707 under stochastic disturbance.

The AE and ACL values were obtained from the PIL simulations for extended with degradation scenario in Table 15.

The average control effort of both surrogates is unchanged at the 1% resolution of the metric, 79% for the ANN and 70% for the AOO-ANFIS. This is a direct consequence of the surrogate structure; both controllers are static maps of the measured pair $i_{s}$ and $i_{s,ref}$ without integral action, so the loss of plant gain is not compensated by larger commands but appears as the larger tracking error and the longer peak times. The execution time of the ANN is again about 2.2 × 10^−7^ s, and that of the AOO-ANFIS, 6.3 × 10^−7^ and 7.3 × 10^−7^ s, lies within the range observed over all PIL runs of this study, between 4.8 × 10^−7^ and 1.2 × 10^−6^ s.

## 4. Discussion

As shown in Table 2, AOO achieved both a lower mean validation fitness and a markedly lower standard deviation than HHO, indicating that AOO converged not only to better but also to more consistently reproducible ANFIS parameterizations across independent runs. The same ordering holds on the held-out test set, where the AOO-tuned ANFIS achieved a lower RMSE for both $D_{p}$ and $D_{m}$. AOO’s curve lies below HHO’s in Figure 2 for the entire run and converges to a visibly lower, tighter plateau, consistent with the summary statistics of Table 2. Based on this comparison, the AOO-tuned ANFIS was selected for the closed-loop PIL evaluation. The Wilcoxon rank-sum test with Holm correction for the four comparisons of Section 3.1 yielded 9.85 × 10^−4^, rejecting the null hypothesis that the two algorithms’ best-fitness distributions are identical and confirming that AOO’s improvement over HHO is statistically significant at the 10-run sample size used. In Figure 3, AOO’s 10 runs are tightly clustered near its median. In contrast, HHO’s runs are both higher in central tendency and considerably more dispersed, including two runs that failed to descend below $J\left(x^{*}\right)=0.09$. In terms of computational cost, the mean wall-clock time per run was 316.7 s for AOO and 211.8 s for HHO on the same hardware, which means that AOO required approximately 50% more time per run to reach its considerably better and more consistent solution. This trade-off is favorable given that ANFIS tuning is an offline, one-time design step rather than a real-time computation. AOO’s membership functions span a mixture of narrow and moderately wide shapes with clearly separated peaks, whereas HHO’s membership functions are consistently broader and more mutually overlapping, particularly for $i_{s}$, where two of its three widths sit at the upper bound of the search range as σ=0.9975≈1 in Figure 4. This lower effective resolution in the input space is a plausible mechanistic explanation for HHO’s higher and less consistent tuning error reported in Table 2. The membership-function-width contrast in Figure 4 is the most visible structural difference between the two tuned solutions and is consistent with HHO’s higher and less stable validation error reported in Table 2. AOO outperforms HHO on MAE and $R^{2}$ metrics, as shown in Table 7, though the margin is more modest than for RMSE, reflecting that the same small number of difficult samples dominates both errors. In Figure 5, the bulk of the test samples for all five optimizers lies close to the identity line, with a visible band of moderately under-predicted points at high $D_{p}$/$D_{m}$ values and a single strong outlier near the lower bound of each output’s physical range at the same transient sample identified during dataset inspection as an isolated, physically anomalous point rather than a general modeling failure.

Under the deterministic disturbance profile, the AOO-ANFIS controller achieved a closed-loop tracking root-mean-squared error of RMSE=0.0447 in Table 9, compared to RMSE=0.0485 for MMPC, resulting in a reduction of approximately 7.84%. Under the stochastic disturbance profile, the improvement was more pronounced as the AOO-ANFIS controller achieved RMSE=0.0485 in Table 9, compared to RMSE=0.0694 for MMPC, resulting in a reduction of approximately 30.12%. As detailed in Table 8 and Table 9, the closed-loop implementation with the AOO-ANFIS structure achieves lower MO and RMSE than the MMPC configuration in both deterministic and stochastic disturbance scenarios. Figure 8 and Figure 14 further demonstrate the reference-tracking capability of the proposed AOO-ANFIS design under both disturbance profiles. The two disturbance tests exercise the controller’s rejection of external torque disturbances and of the time-varying volumetric efficiencies in Figure 10 and Figure 16, but they do not by themselves constitute a parametric robustness test; the sensitivity to a degradation of the plant’s volumetric efficiencies is therefore examined separately in Section 3.4. Fluctuations in volumetric efficiencies and the corresponding variations in pump and motor angular velocities (Figure 9, Figure 10, Figure 15 and Figure 16) lead to more frequent overshoots under the MMPC compared to the AOO-ANFIS framework. Additionally, Figure 8, Figure 9, Figure 10 and Figure 11 and Figure 14, Figure 15, Figure 16 and Figure 17 confirm that the MMPC algorithm induces heightened oscillatory behavior. Consequently, the proposed approach proves superior to the MMPC scheme in rejecting both deterministic in Figure 7 and stochastic in Figure 13 perturbations. Quantitative metrics from Table 10, alongside Figure 11, Figure 12, Figure 17 and Figure 18, highlight that the AOO-ANFIS scheme operates with a lower AE value of about 10% and lower ACL values of about 76% (3.65 × 10^−6^ s) under deterministic disturbances and 84% (4.797 × 10^−6^ s) under stochastic disturbances, offering a smoother control action with lower average control effort (AE). Conversely, the MMPC architecture exhibits higher values; its elevated computational demands reflect an aggressive control strategy characterized by high-frequency oscillations. Overall, the AOO-ANFIS achieved lower overshoot, lower tracking error and lower control effort than the MMPC under both disturbance profiles. The PIL simulations verified that the object code generated for the BeagleBone Black reproduced the control signals of the Simulink model to within numerical precision, and that the measured execution time per controller step, the ACL of Table 10, was roughly three orders of magnitude below the controller sample time at 1 ms, about 850 times smaller in the deterministic and 1100 times smaller in the stochastic case. Furthermore, the AOO-ANFIS controller outperformed the MMPC framework in terms of AE and ACL, as summarized in Table 10. These results validate the suitability of the BeagleBone Black board as an efficient target hardware platform.

Overall, the PIL results affirm the high efficacy of the proposed AOO-ANFIS architecture. It successfully maintained precise trajectory tracking under abrupt and stochastic torque disturbances and under the time-varying volumetric efficiencies of the plant, yielding favorable MO, RMSE and $t_{p}$ values compared to the MMPC, which continuously optimizes $D_{p}$ and $D_{m}$. Functioning as a data-driven surrogate model trained on operational data generated by the MMPC, the proposed AOO-ANFIS emulates the robust control capability of the optimization-heavy MPC while eliminating its computational bottleneck during implementation.

At first sight, it may appear paradoxical that a controller fitted to the MMPC’s outputs achieves lower overshoot and lower tracking error than the MMPC itself. Three mechanisms explain the result, and the first is directly visible in the closed-loop signals. The MMPC of [18] consists of two MPCs designed on discrete-time linear models of the primary and secondary regulation operating points, with a switching signal selecting the active controller. Figure 11a shows that at the switching instants at t ≈ 72, 118 and 161 s, the MMPC momentarily drives the pump displacement from its upper limit to zero and back. Because the pump volumetric efficiency is lowest at zero displacement as 0.56, these events appear as sharp drops of $\eta_{vp}$ in Figure 10a, and at t ≈ 71 and 161 s in Figure 16a, and as overshoot in the stroke response. The AOO-ANFIS interpolates smoothly between its nine rules and does not produce such pump-displacement collapses in Figure 11b; only a single brief motor-displacement transient occurs at t ≈ 141 s. The fuzzy approximation therefore acts as a regularization of the MMPC policy, removing its switching transients while preserving the underlying low-frequency control law. The prediction models of the MMPC are linear time invariant, so the volumetric efficiencies inside them are fixed at their operating-point values. In contrast, in the nonlinear plant, they vary with speed and displacement, approximately between 0.80 and 0.90 for $\eta_{vp}$ and between 0.79 and 0.90 for $\eta_{vm}$ during the runs, as Figure 10 and Figure 16 show. The MMPC’s predictions are therefore systematically biased away from its design points. The surrogate has no internal model; it was fitted to closed-loop data generated with the full nonlinear, time-varying plant, and its output depends only on the measured ($i_{s}$, $i_{s,ref}$) pair. Candidates are scored on a validation subset in Equation (39) rather than on the training subset, which biases the optimizer toward parameter vectors that reproduce the MMPC’s average behavior rather than its sample-to-sample variations. Similar observations have been reported for neural-network approximations of MPC [72,73] and motivate the approximate-MPC literature reviewed in [80]. The price of this regularization is evident in the stochastic case, where the AOO-ANFIS’s smoother action increases the peak time from 1 s to 1.8 s in Table 8, and in the deterministic absolute-error indices in Table 9, which penalize the sustained small lag in the smoother response. The extended scenario of Section 3.3 shows that the same smoothness carries over outside the training data: the regularized surrogate remains bounded and accurate on reference segments and disturbance classes it has never seen. Across all PIL runs of this study, including the extended and degraded ones, the execution time per step remained at about 2.2 × 10^−7^ s for the ANN and between 4.8 × 10^−7^ and 1.2 × 10^−6^ s for the AOO-ANFIS, independent of the scenario and the plant condition.

## 5. Conclusions

This study proposed AOO-ANFIS, a novel controller architecture that is the first to combine an ANFIS with the AOO algorithm and the first bio-inspired optimization-tuned ANFIS controller applied to HST stroke control. Five population-based tuning algorithms, AOO, HHO, PSO, a real-coded GA and CMA-ES, were compared on an identical 66-parameter tuning task derived from a previously validated MMPC dataset [18]. Across 10 independent runs, AOO achieved the lowest mean validation fitness of about $3.65\times 10^{-2}$ against 6.44 × 10^−2^ for HHO, 3.68 × 10^−2^ for PSO, 3.90 × 10^−2^ for GA and 3.99 × 10^−2^ for CMA-ES; its advantage over HHO, GA and CMA-ES is statistically significant after Holm correction, whereas AOO and PSO are statistically indistinguishable. Inspection of the tuned membership functions of AOO and HHO showed that HHO converged to broader, more mutually overlapping fuzzy sets than AOO, offering a plausible mechanistic explanation for its weaker and less consistent performance.

The AOO-ANFIS was deployed in PIL simulation on the same BeagleBone Black target and under the same deterministic and stochastic torque-disturbance profiles used to validate the MMPC. Under the deterministic profile, the AOO-ANFIS reduced the closed-loop tracking RMSE by approximately 7.84% relative to the MMPC; under the stochastic profile, the reduction was approximately 30.12%. The maximum overshoot fell from 25% to 14% and from 24% to 14%, respectively; the integral squared-error indices ISE and ITSE fell by 15% and 21% under deterministic and by 51% and 41% under stochastic disturbances, and under stochastic disturbances, all four integral indices were the lowest of the three controllers compared. The average control effort was about 10 percentage points lower, and the average computational load was 76% lower under deterministic and 84% lower under stochastic disturbances than that of the MMPC, at the cost of a longer peak time under stochastic disturbances, 1.8 s instead of 1 s. For an embedded real-time controller, this reduction in computational burden is as practically significant as the accompanying improvements in tracking error and overshoot, since it directly relaxes the processing requirements of the target hardware.

In an extended 300 s scenario containing reference levels, ramp rates, a sinusoidal segment and trapezoidal torque disturbances absent from the training data, the AOO-ANFIS tracked the unseen segment with an RMSE of 0.035 under deterministic and 0.041 under stochastic disturbances, lower than the 0.056 and 0.062 of the ANN surrogate of [19] trained on the same data, and both surrogates retained stable tracking with the volumetric-efficiency maps of the plant degraded by about 15%. The AOO-ANFIS again had lower error, and both had unchanged control effort and execution time per step. The surrogate has therefore learned the control law of its teacher rather than the training trajectory, and within the physical envelope of the plant, it generalizes to reference segments and disturbance classes that were never part of its training data.

Future work will extend the surrogate with an online adaptation mechanism so that it can track operating regimes beyond the envelope examined here, and apply the AOO-ANFIS framework to other fuzzy-system architectures.

## Figures and Tables

**Figure 19:**
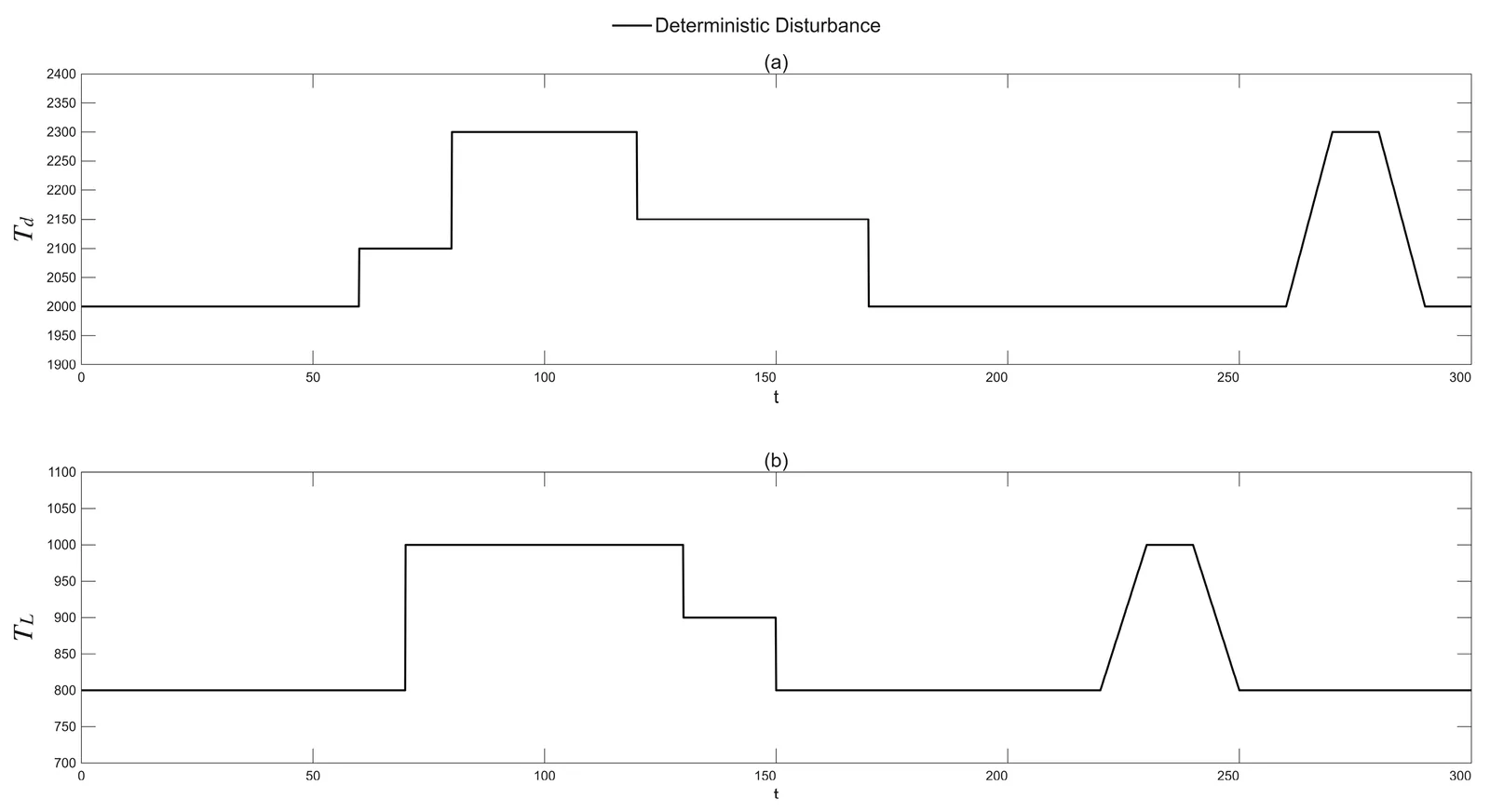
Deterministic torque disturbances for extended scenario: (**a**) $T_{d}$ and (**b**) $T_{L}$.

**Figure 20:**
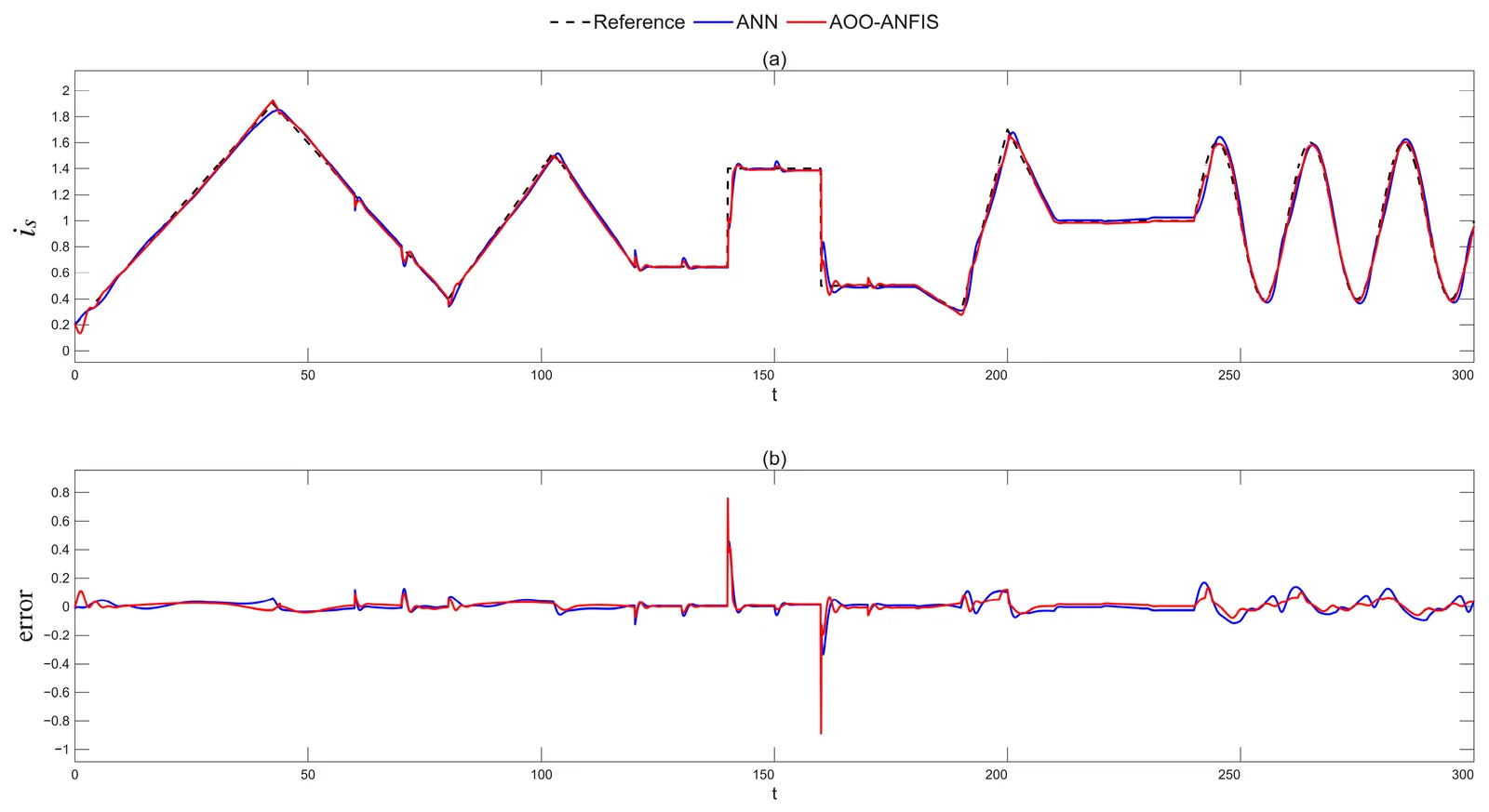
(**a**) Stroke responses and (**b**) errors of the closed-loop systems with ANN and AOO-ANFIS in the case of deterministic torque disturbances for extended scenario.

**Figure 21:**
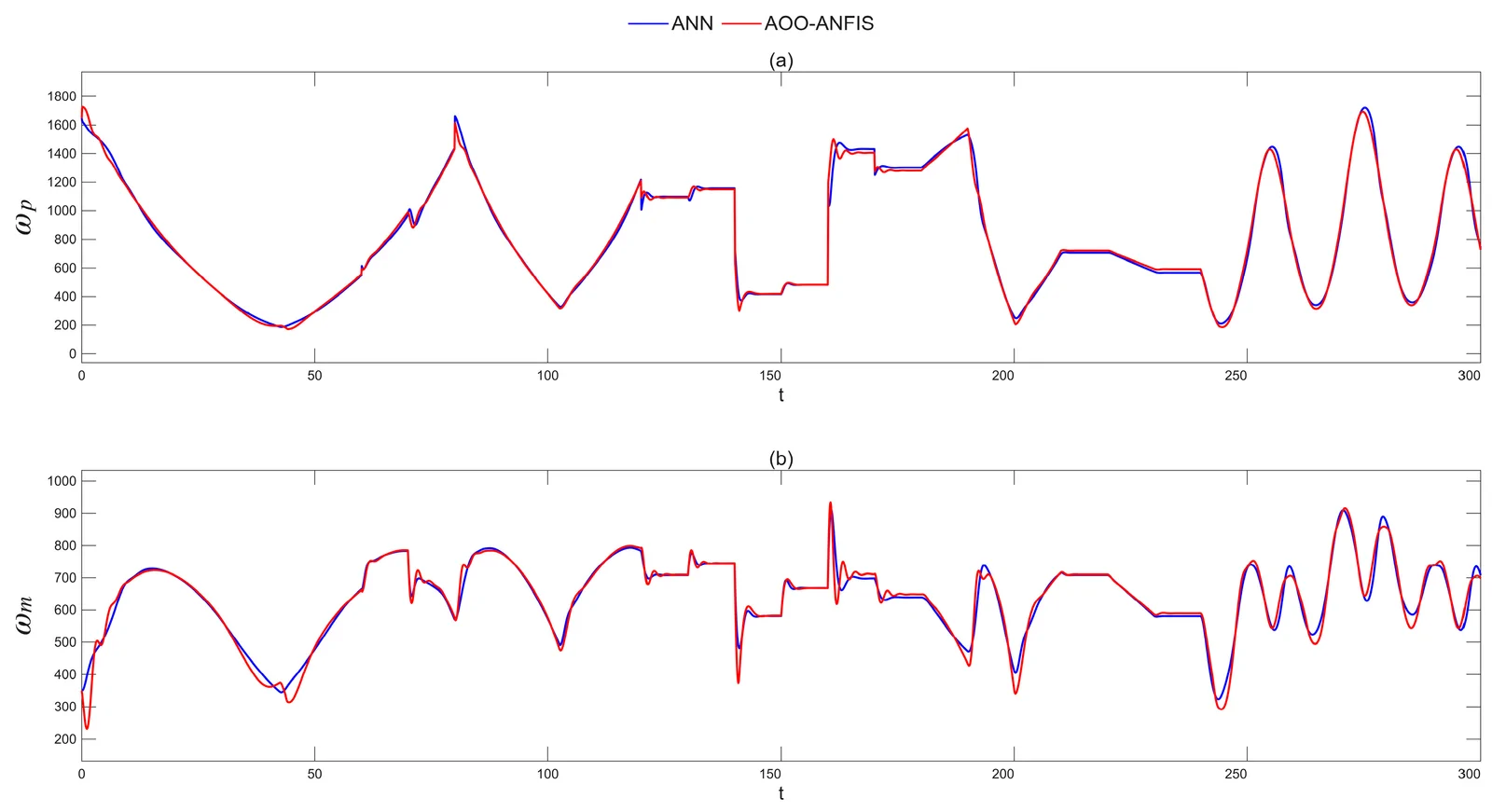
Angular speeds of the (**a**) pump and (**b**) motor of the closed-loop systems with ANN and AOO-ANFIS in the case of deterministic torque disturbances for extended scenario.

**Figure 22:**
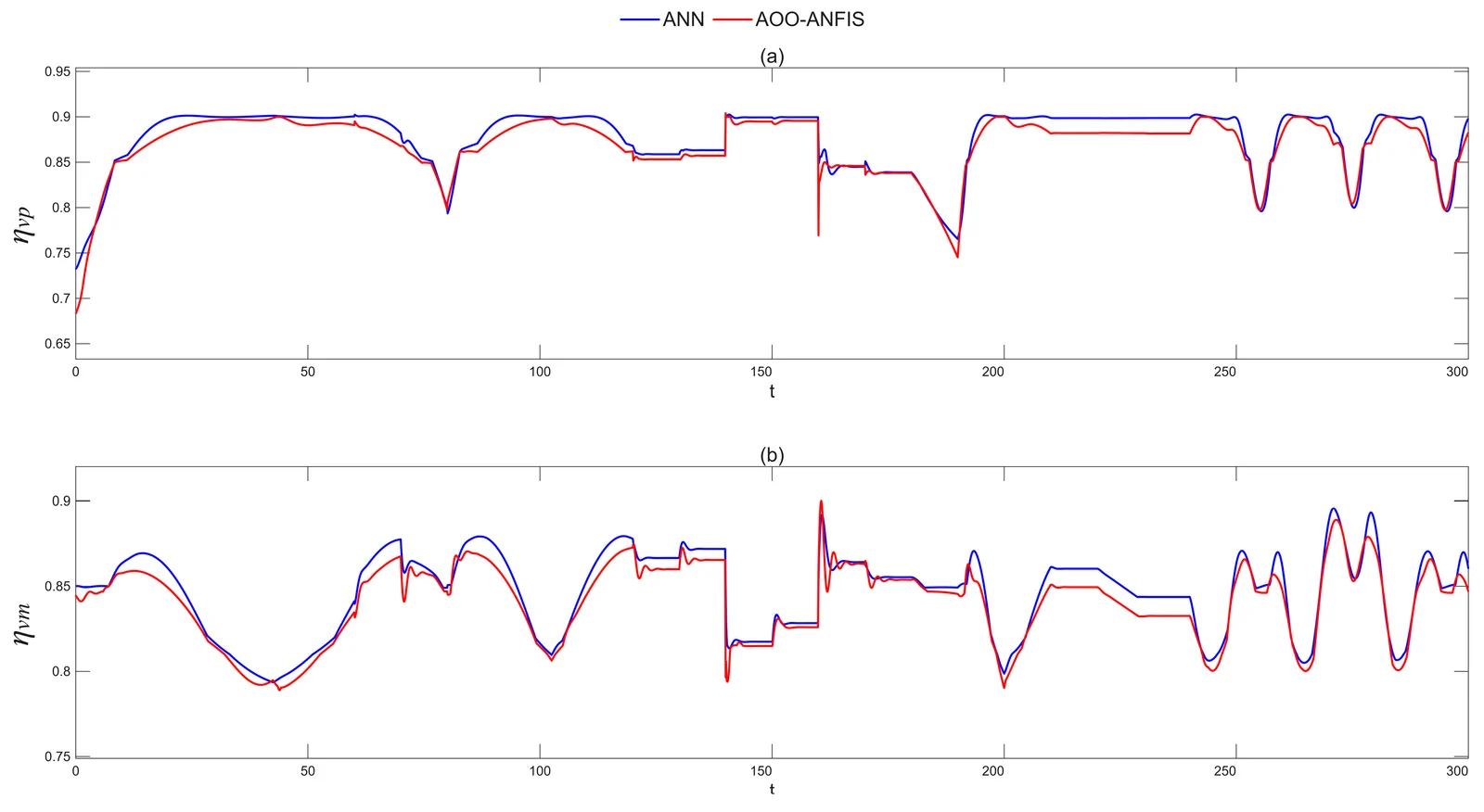
Volumetric efficiencies of the (**a**) pump and (**b**) motor of the closed-loop systems with ANN and AOO-ANFIS in the case of deterministic torque disturbances for extended scenario.

**Figure 23:**
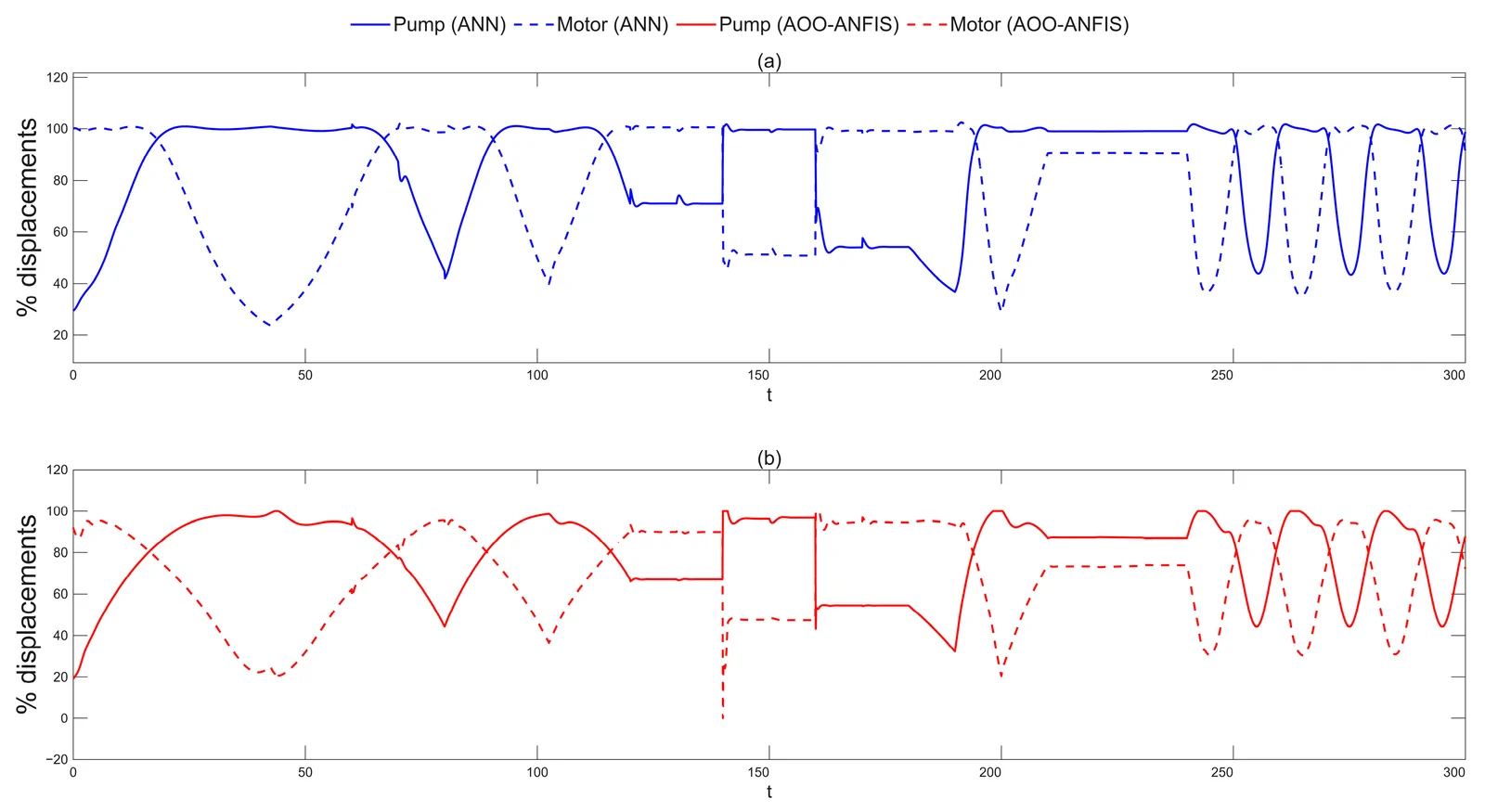
Normalized percentage of pump and motor displacements of the closed-loop systems with (**a**) ANN and (**b**) AOO-ANFIS in the case of deterministic torque disturbances for extended scenario.

**Figure 24:**
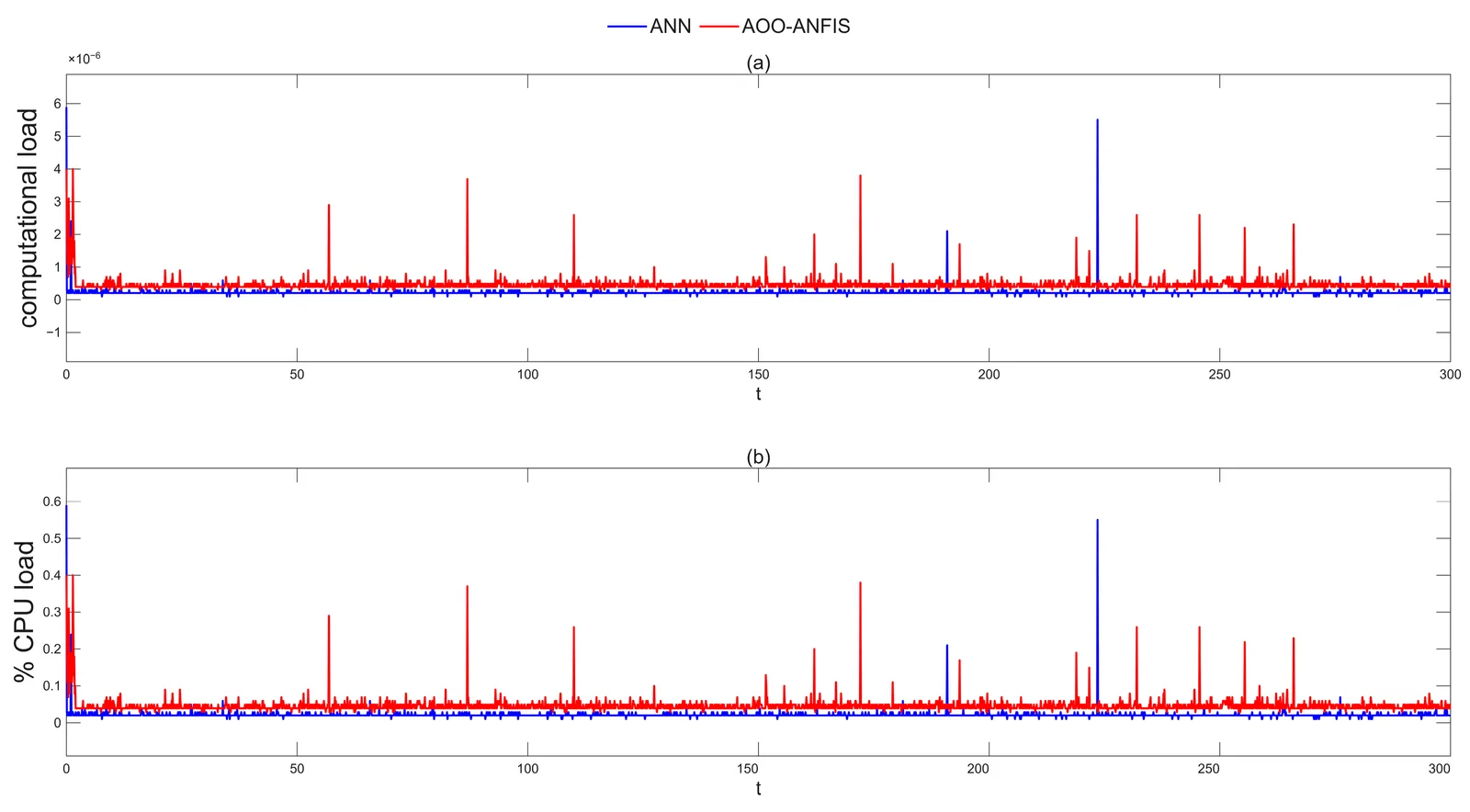
(**a**) Computational and (**b**) percentage CPU loads in the case of deterministic torque disturbances for extended scenario.

**Figure 25:**
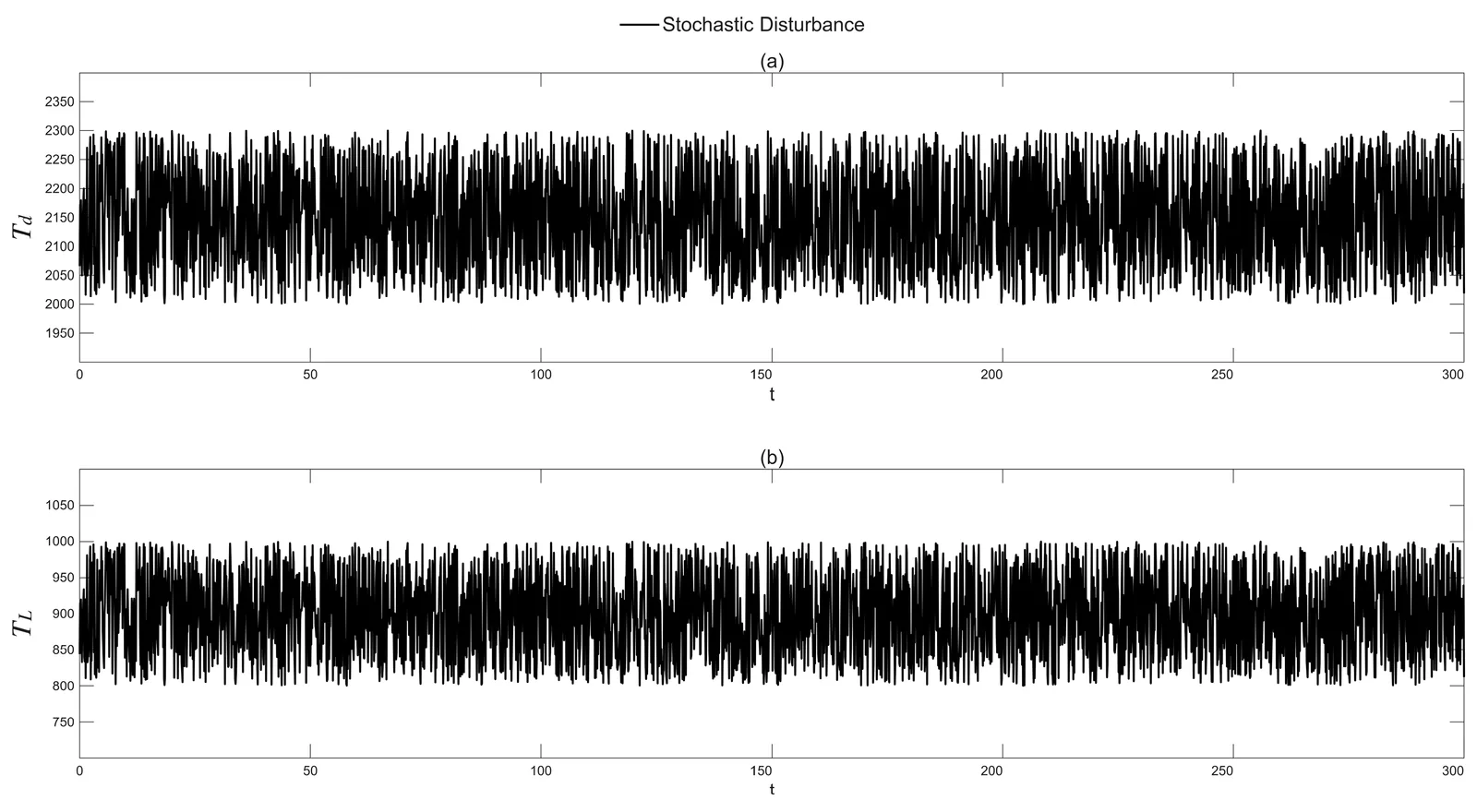
Stochastic torque disturbances for extended scenario: (**a**) $T_{d}$ and (**b**) $T_{L}$.

**Figure 26:**
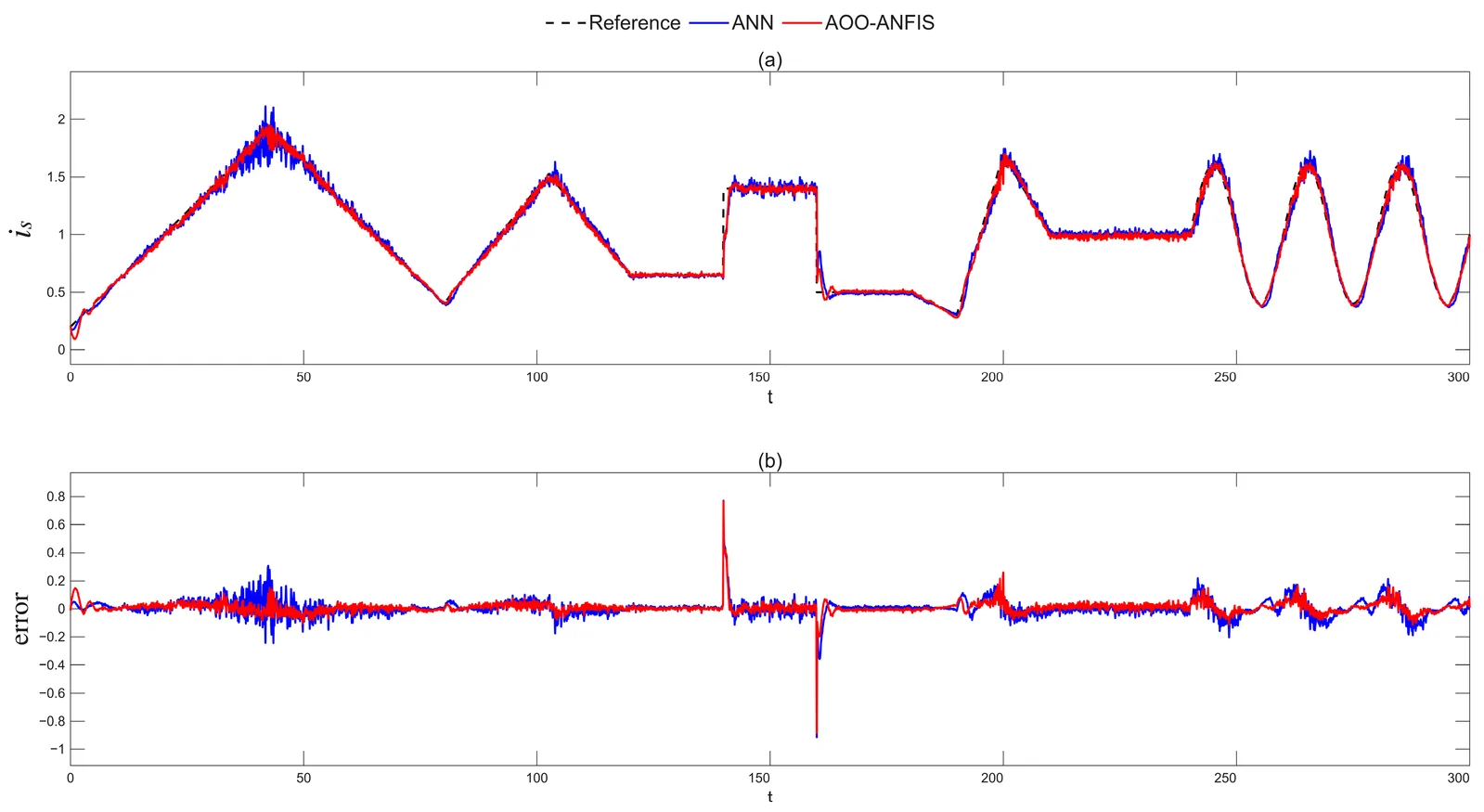
(**a**) Stroke responses and (**b**) errors of the closed-loop systems with ANN and AOO-ANFIS in the case of stochastic torque disturbances for extended scenario.

**Figure 27:**
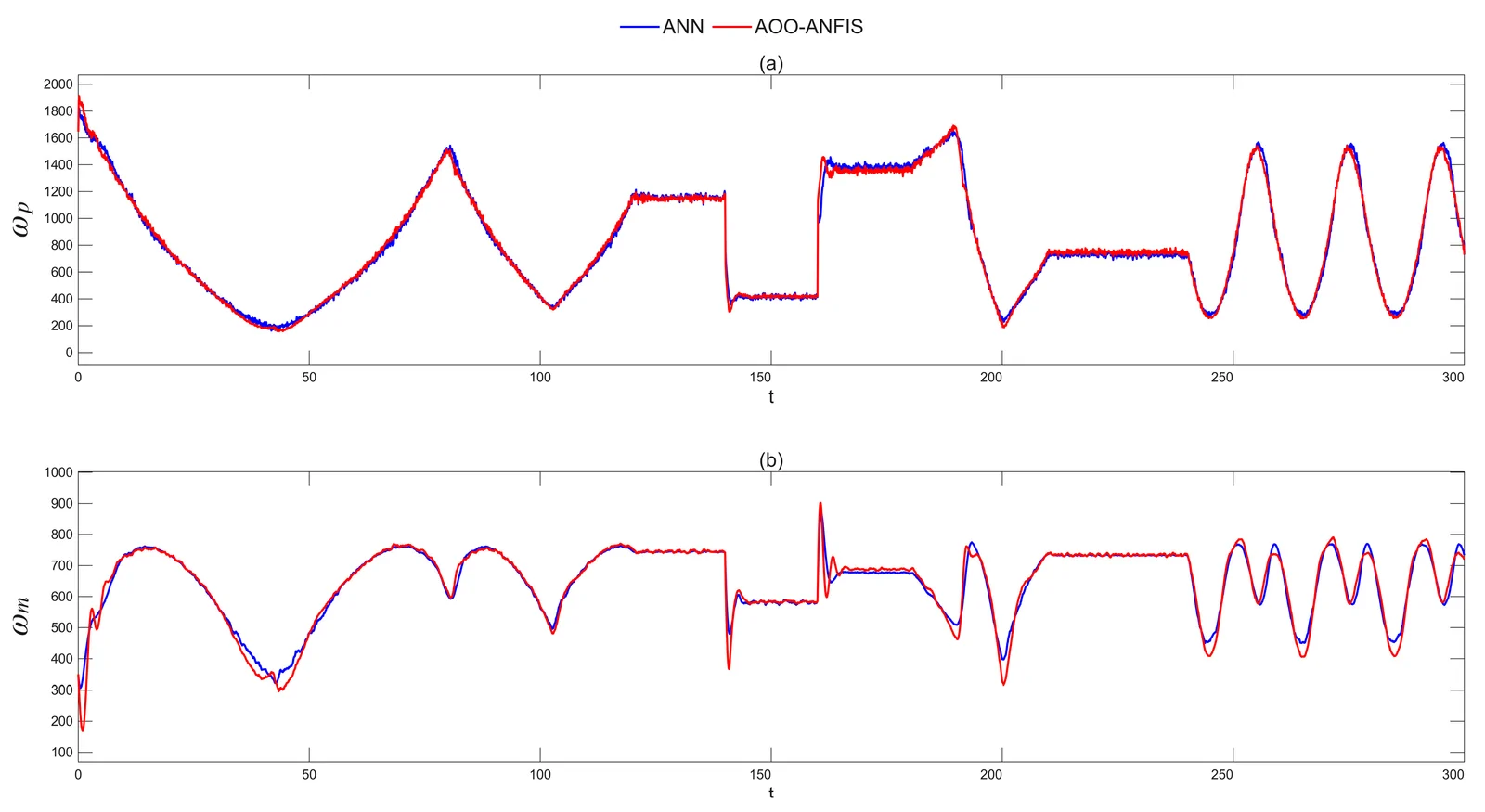
Angular speeds of the (**a**) pump and (**b**) motor of the closed-loop systems with ANN and AOO-ANFIS in the case of stochastic torque disturbances for extended scenario.

**Figure 28:**
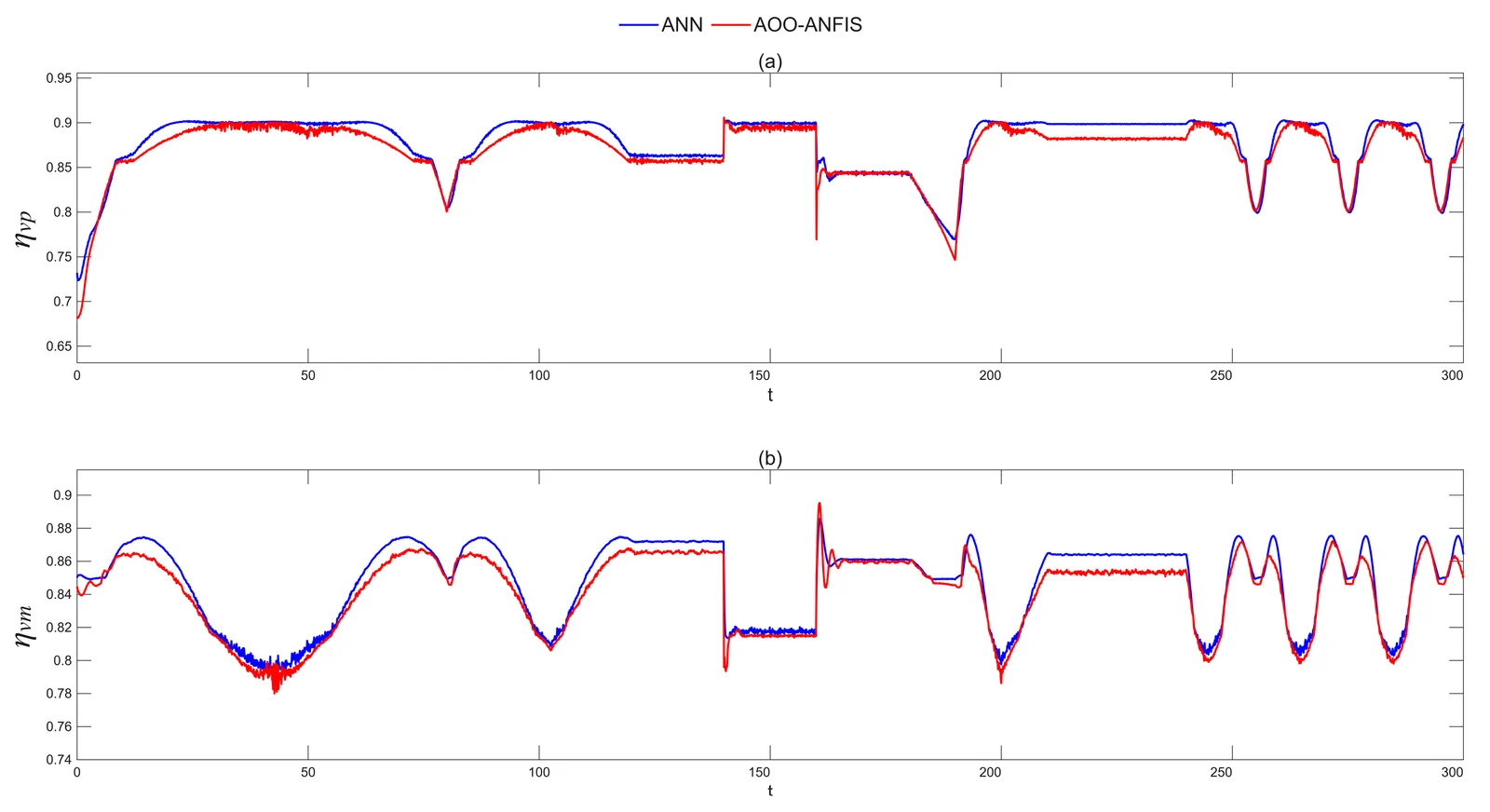
Volumetric efficiencies of the (**a**) pump and (**b**) motor of the closed-loop systems with ANN and AOO-ANFIS in the case of stochastic torque disturbances for extended scenario.

**Figure 29:**
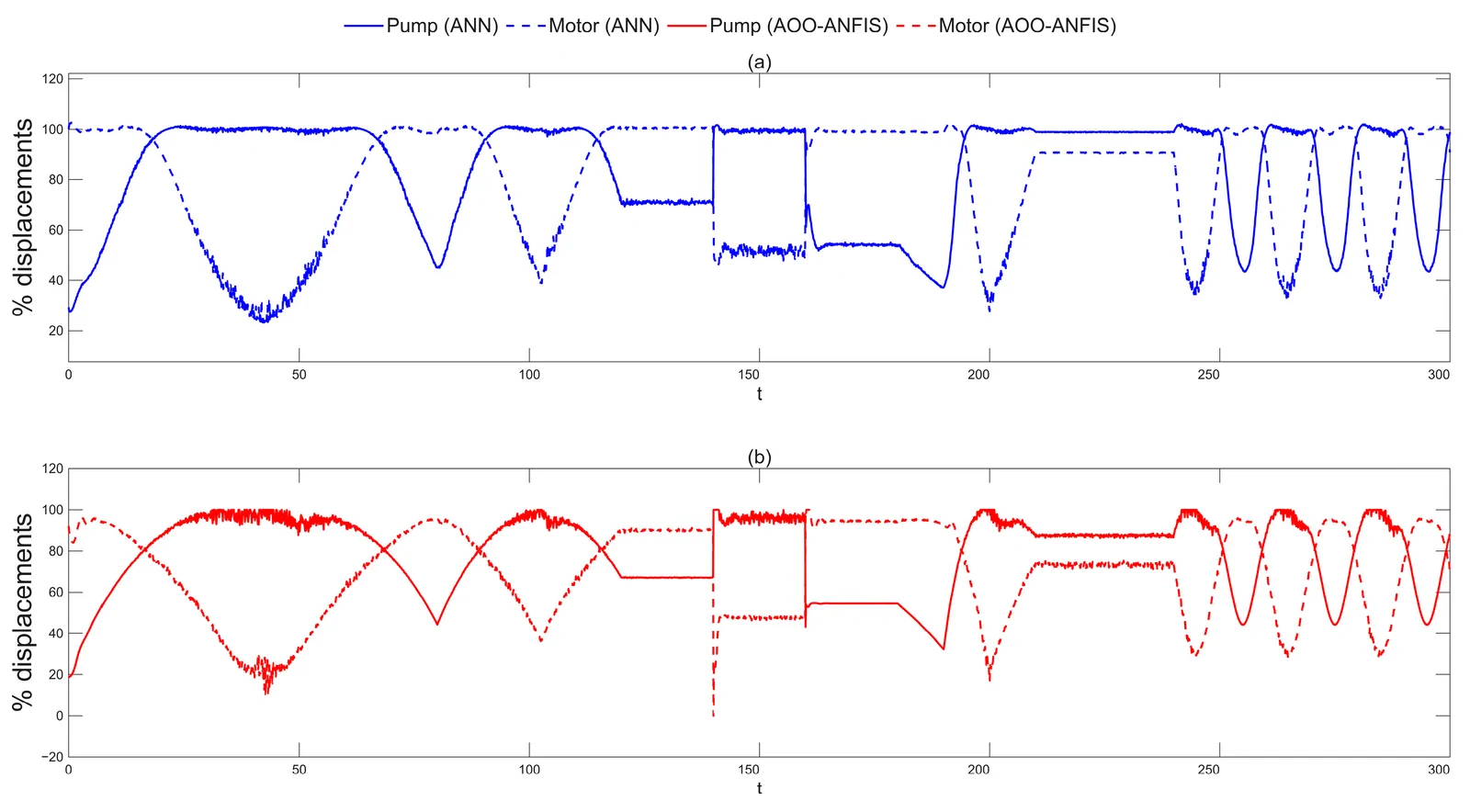
Normalized percentage of pump and motor displacements of the closed-loop systems with (**a**) ANN and (**b**) AOO-ANFIS in the case of stochastic torque disturbances for extended scenario.

**Figure 30:**
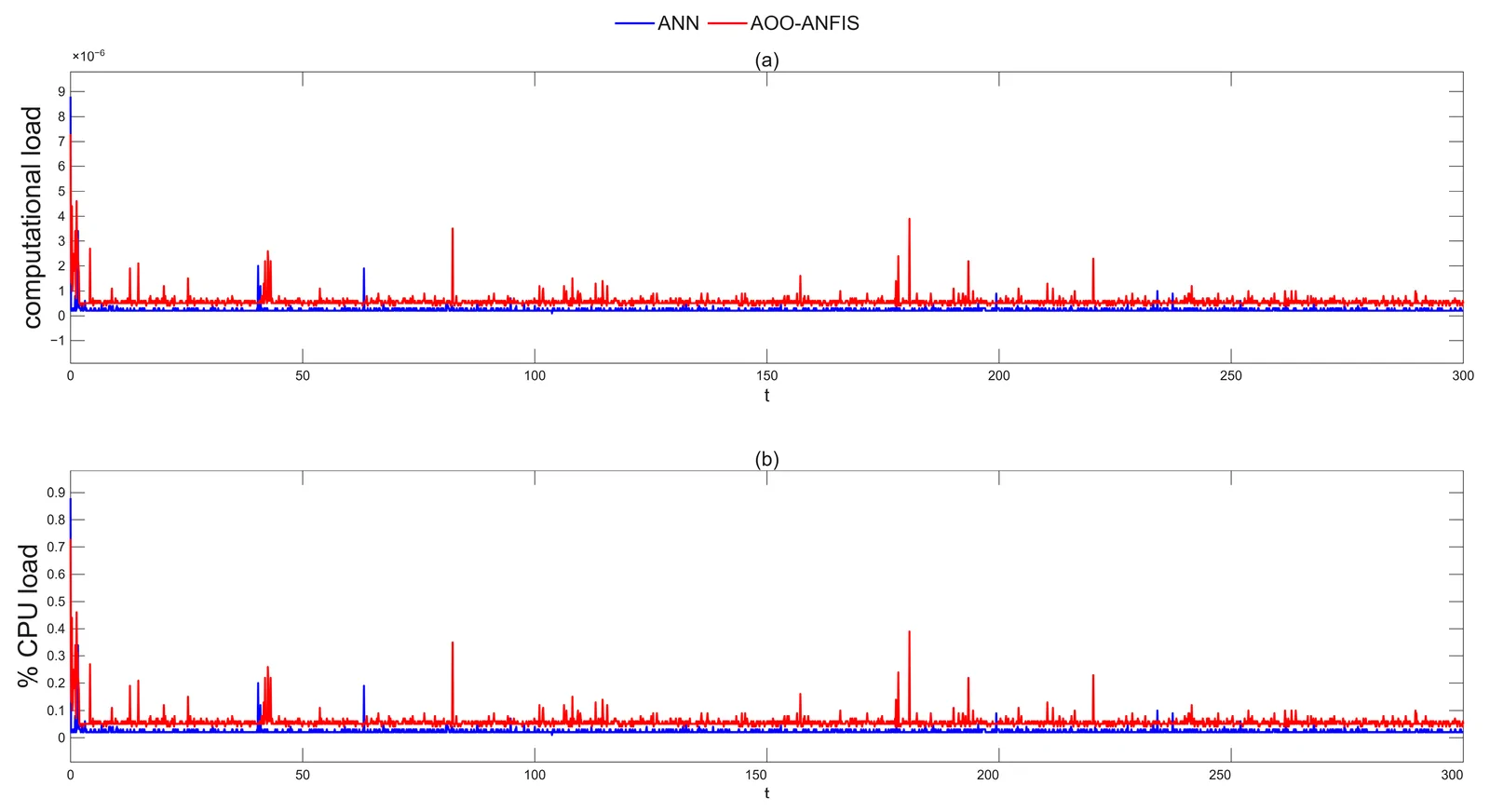
(**a**) Computational and (**b**) percentage CPU loads in the case of stochastic torque disturbances for extended scenario.

**Figure 31:**
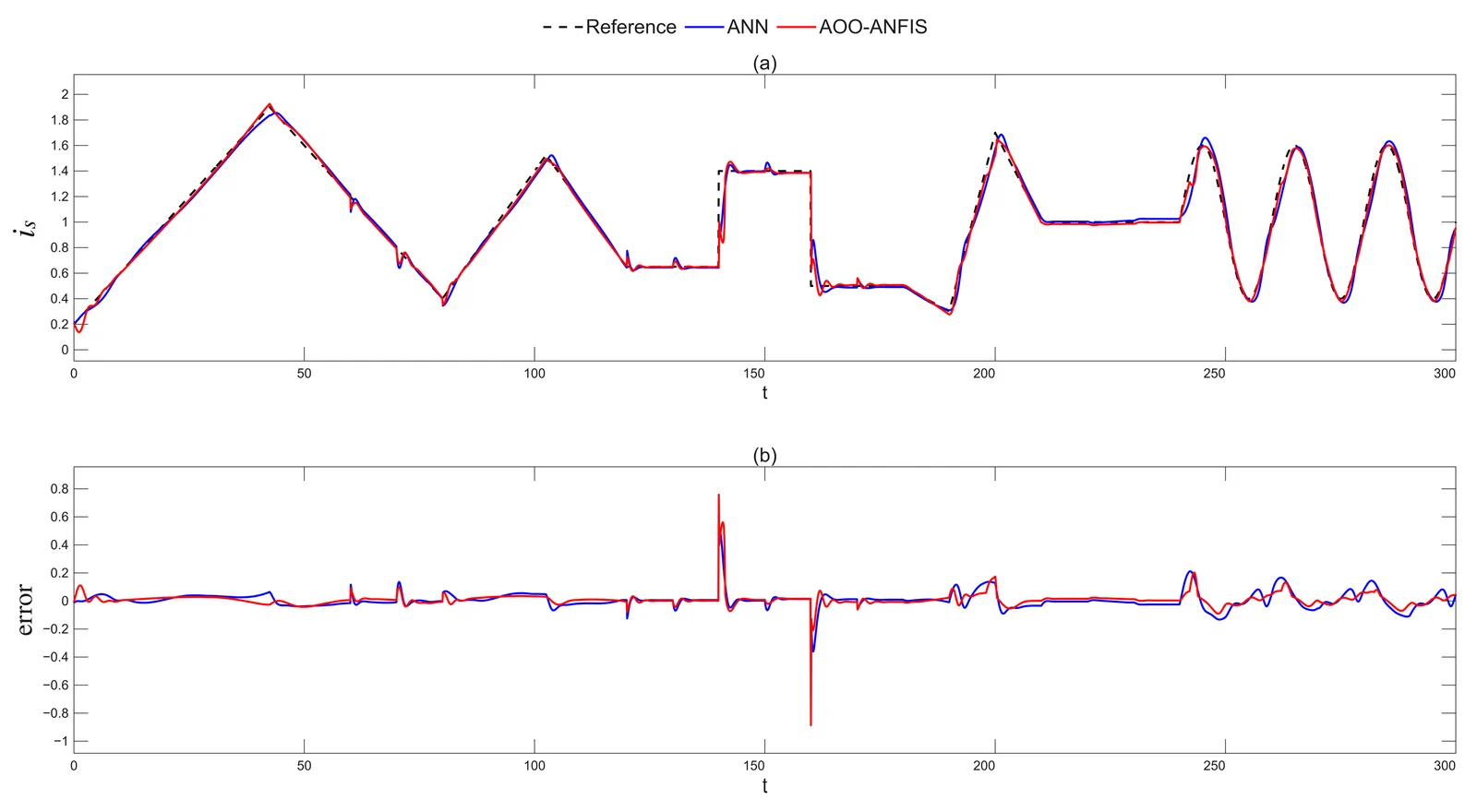
(**a**) Stroke responses and (**b**) errors of the closed-loop systems with ANN and AOO-ANFIS in the case of deterministic torque disturbances for extended with degradation scenario.

**Figure 32:**
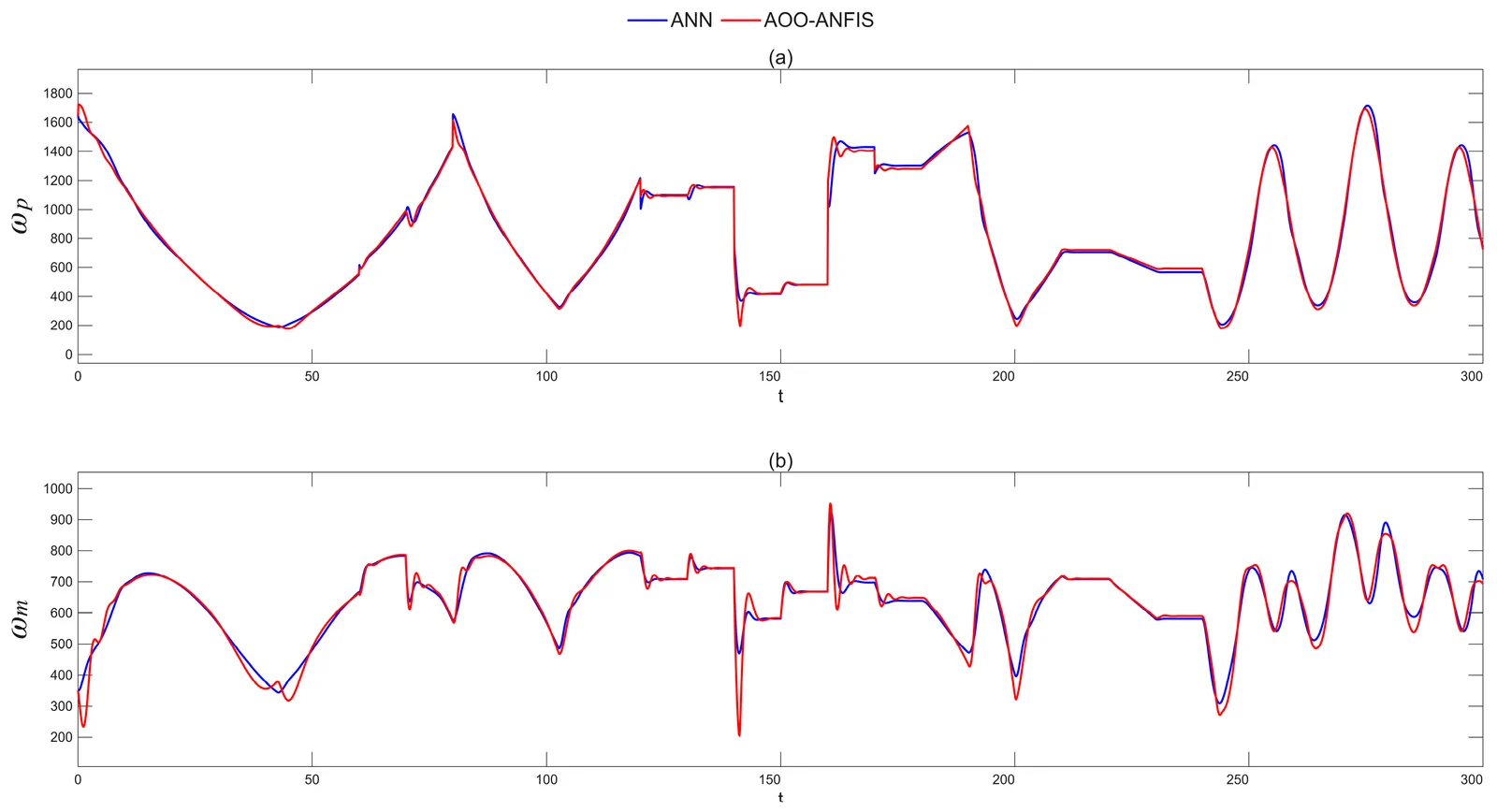
Angular speeds of the (**a**) pump and (**b**) motor of the closed-loop systems with ANN and AOO-ANFIS in the case of deterministic torque disturbances for extended with degradation scenario.

**Figure 33:**
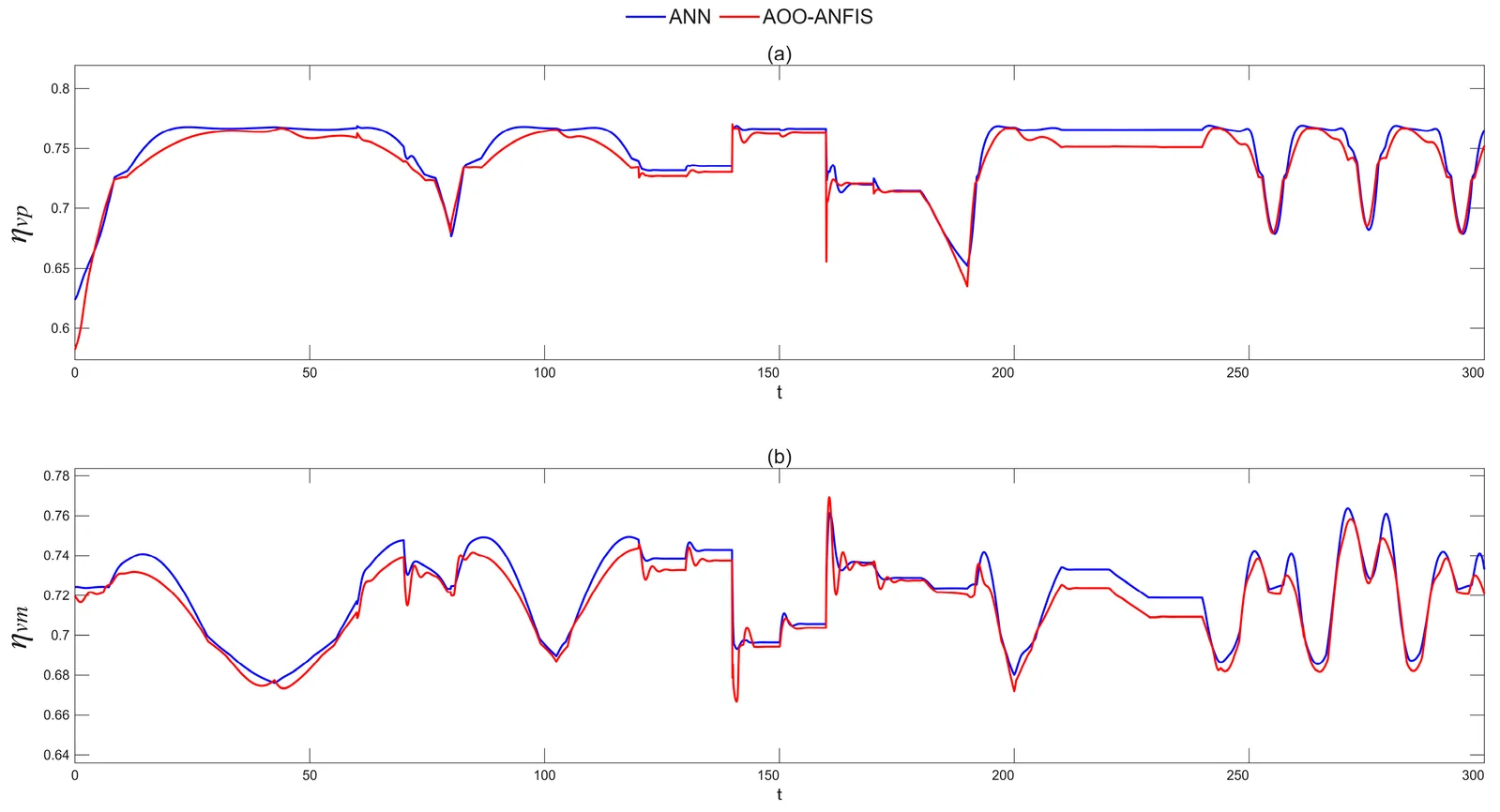
Volumetric efficiencies of the (**a**) pump and (**b**) motor of the closed-loop systems with ANN and AOO-ANFIS in the case of deterministic torque disturbances for extended with degradation scenario.

**Figure 34:**
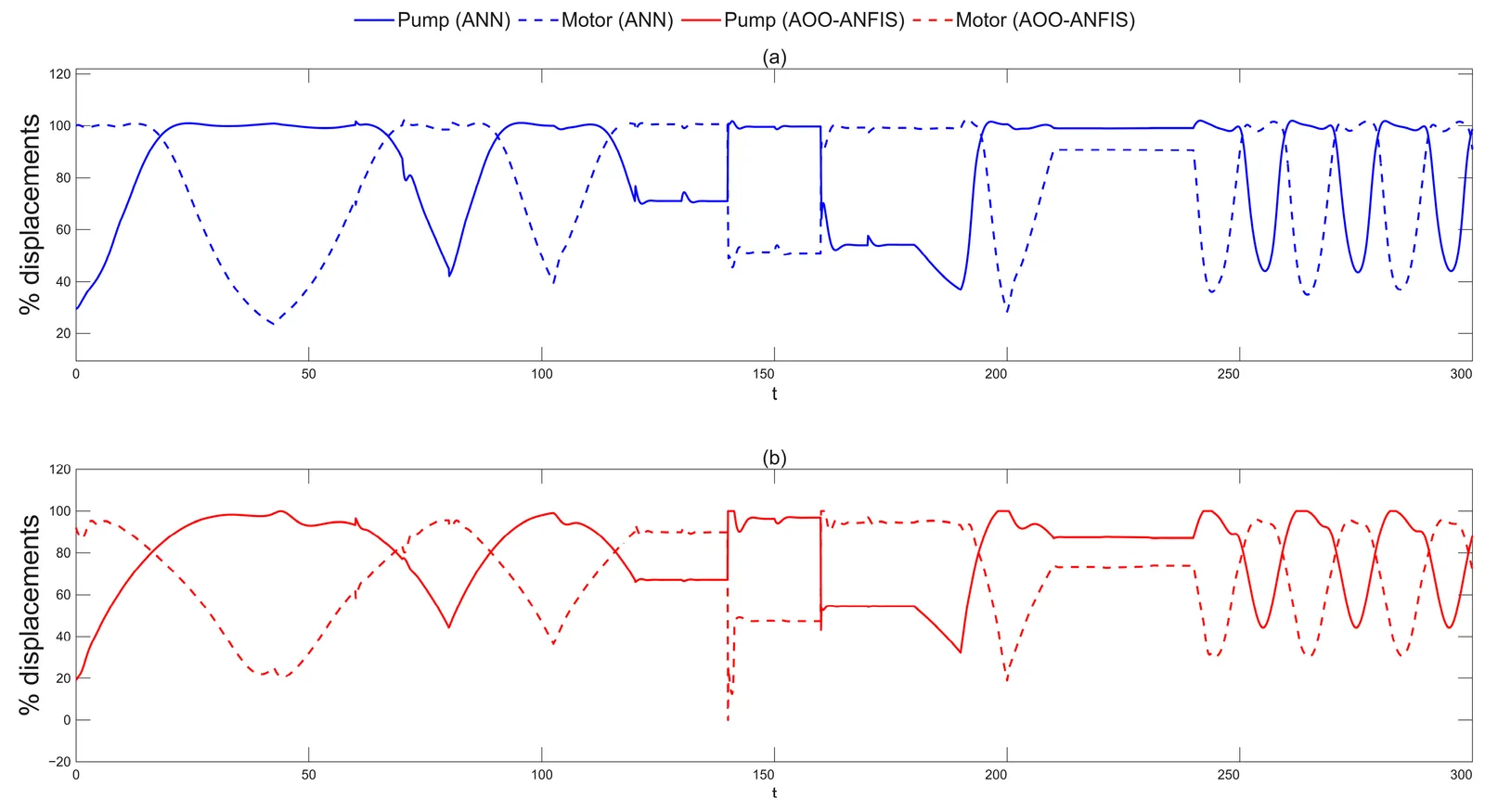
Normalized percentage of pump and motor displacements of the closed-loop systems with (**a**) ANN and (**b**) AOO-ANFIS in the case of deterministic torque disturbances for extended with degradation scenario.

**Figure 35:**
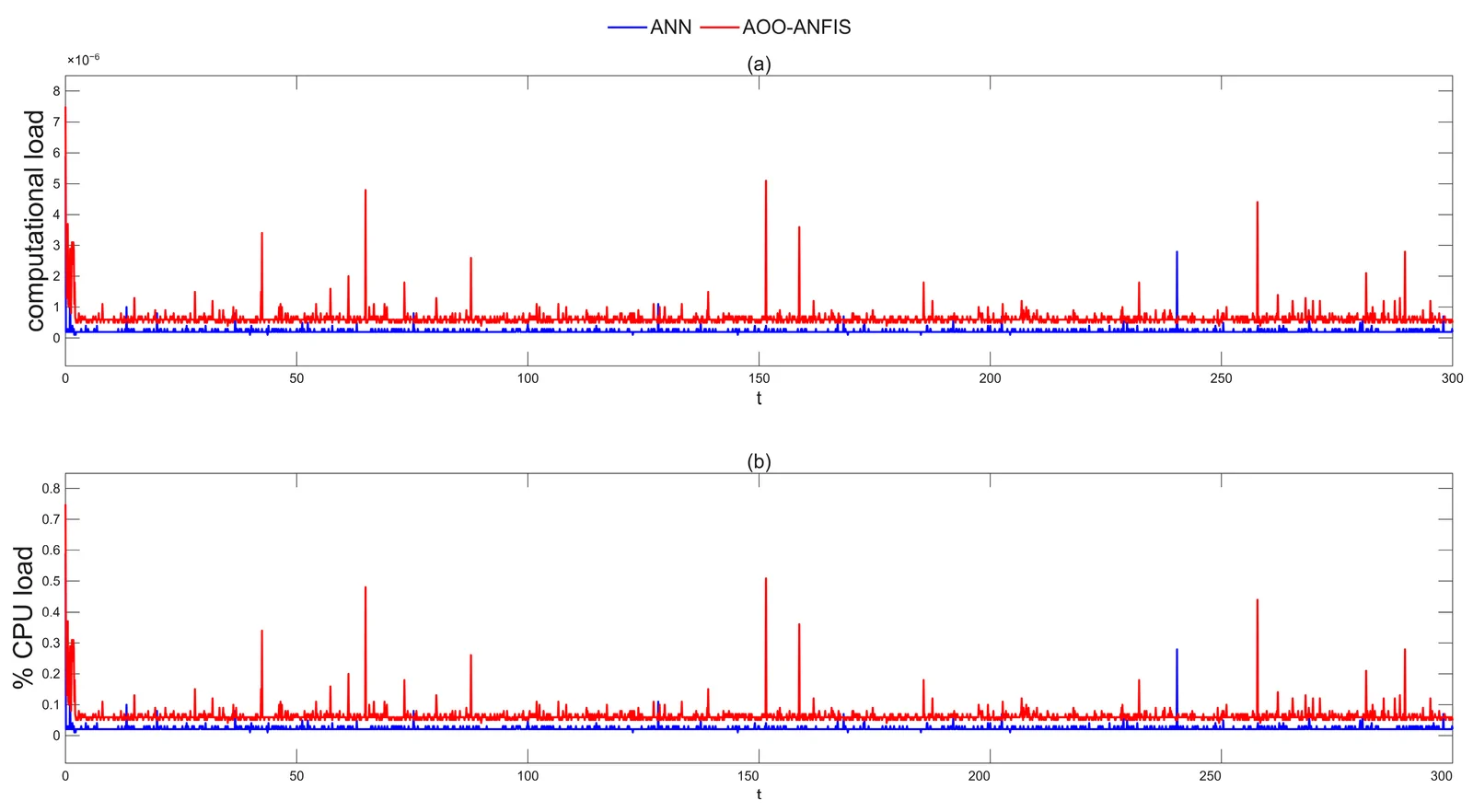
(**a**) Computational and (**b**) percentage CPU loads in the case of deterministic torque disturbances for extended with degradation scenario.

**Figure 36:**
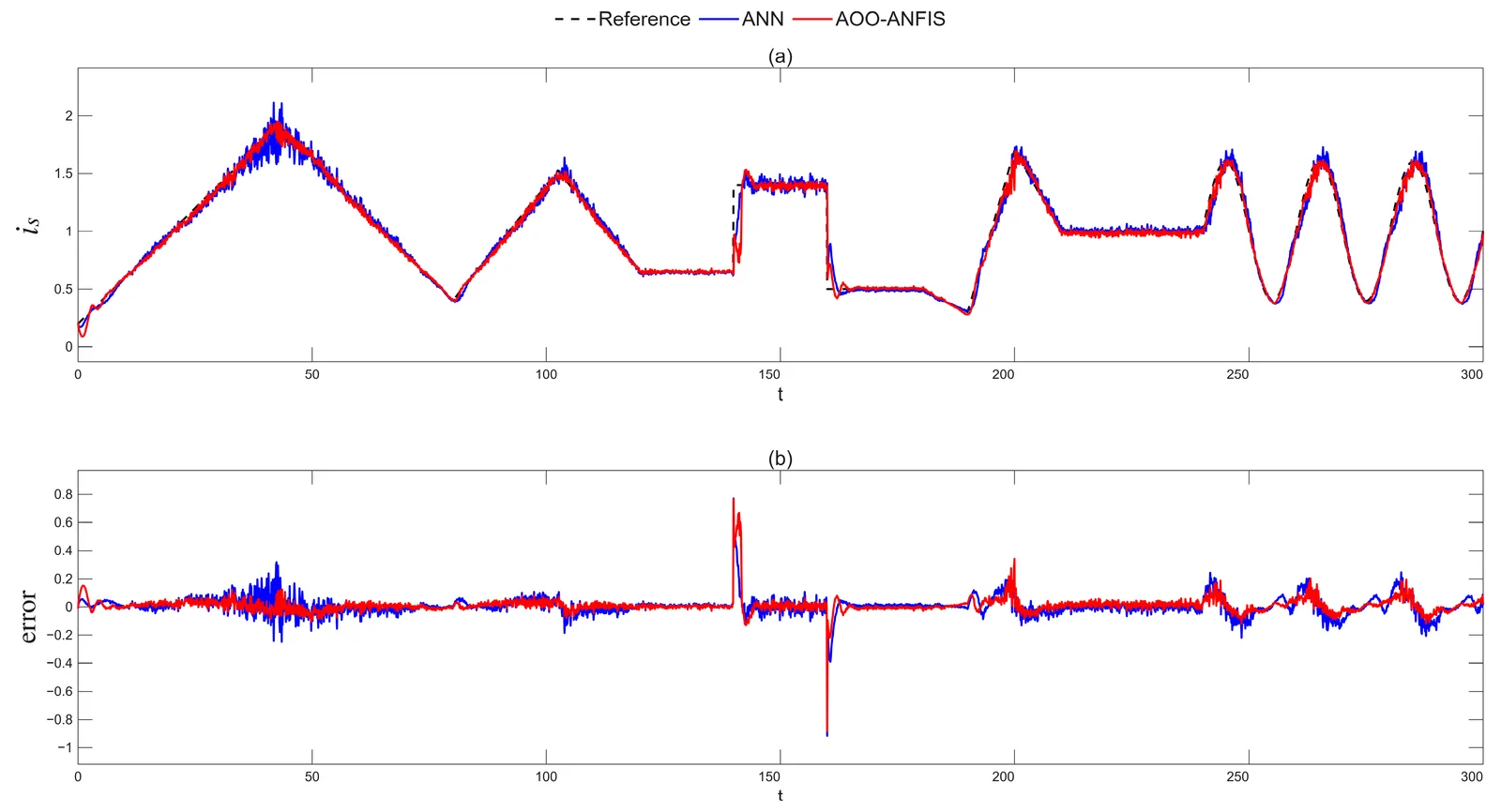
(**a**) Stroke responses and (**b**) errors of the closed-loop systems with ANN and AOO-ANFIS in the case of stochastic torque disturbances for extended with degradation scenario.

**Figure 37:**
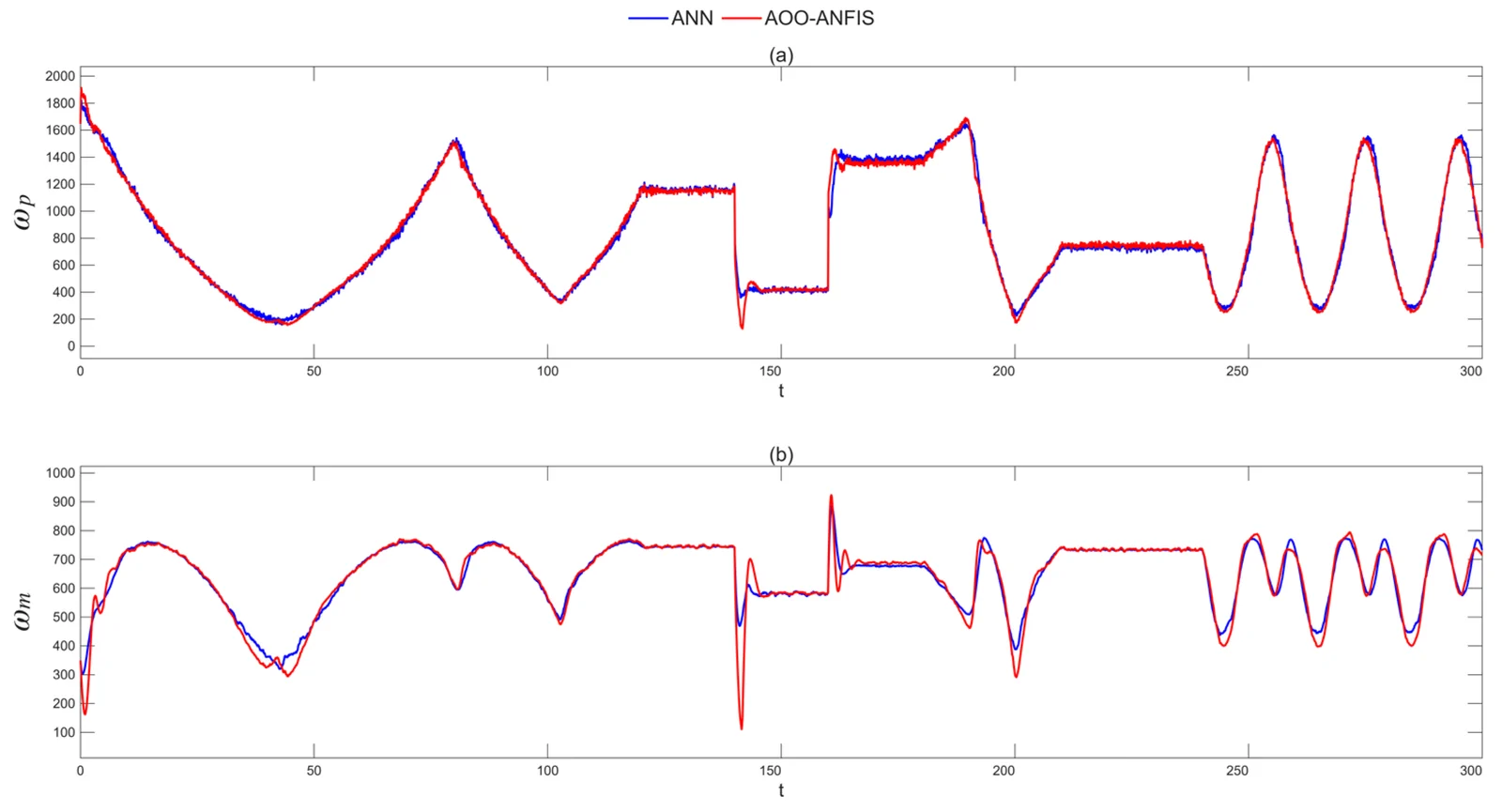
Angular speeds of the (**a**) pump and (**b**) motor of the closed-loop systems with ANN and AOO-ANFIS in the case of stochastic torque disturbances for extended with degradation scenario.

**Figure 38:**
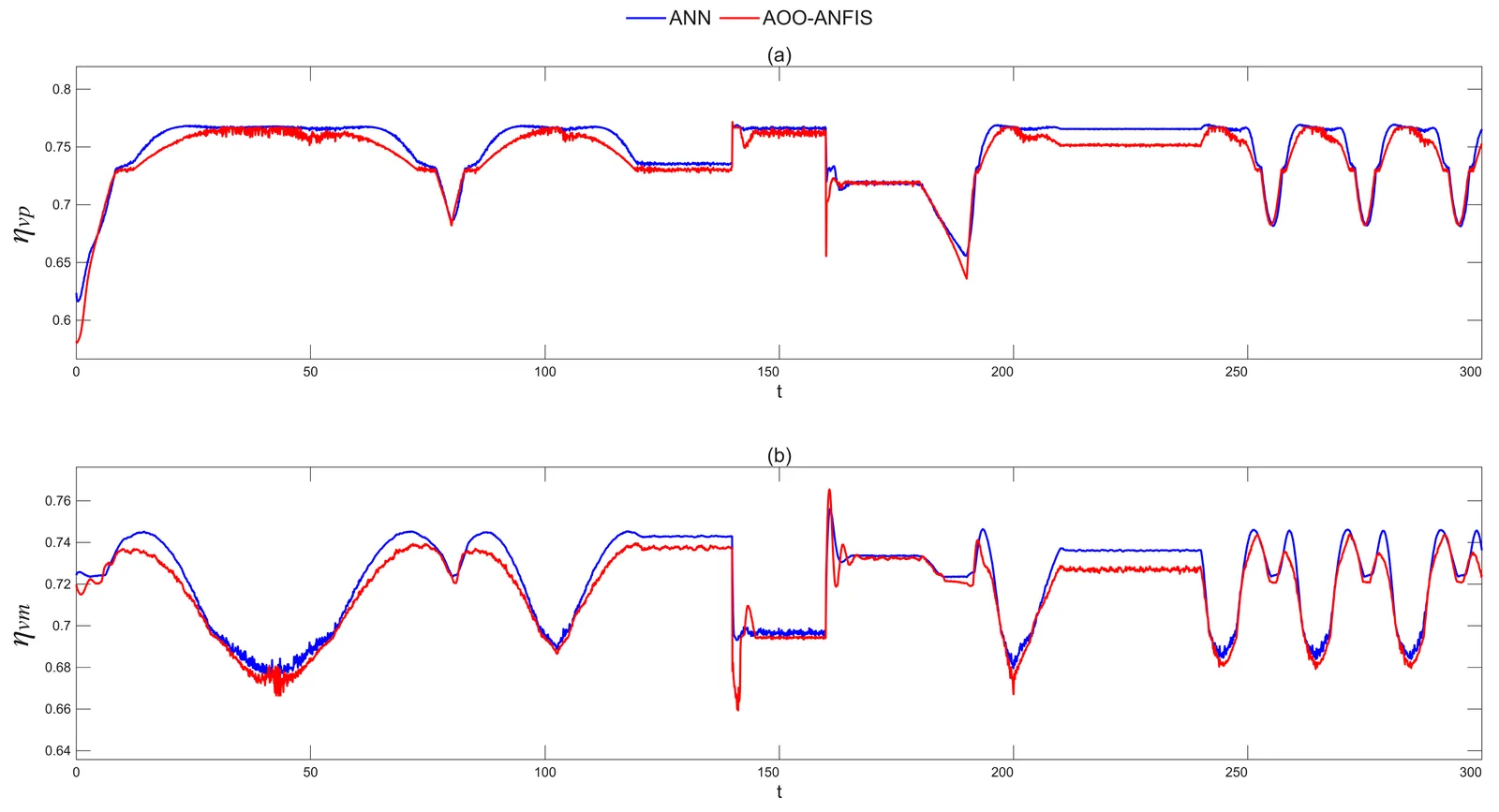
Volumetric efficiencies of the (**a**) pump and (**b**) motor of the closed-loop systems with ANN and AOO-ANFIS in the case of stochastic torque disturbances for extended with degradation scenario.

**Figure 39:**
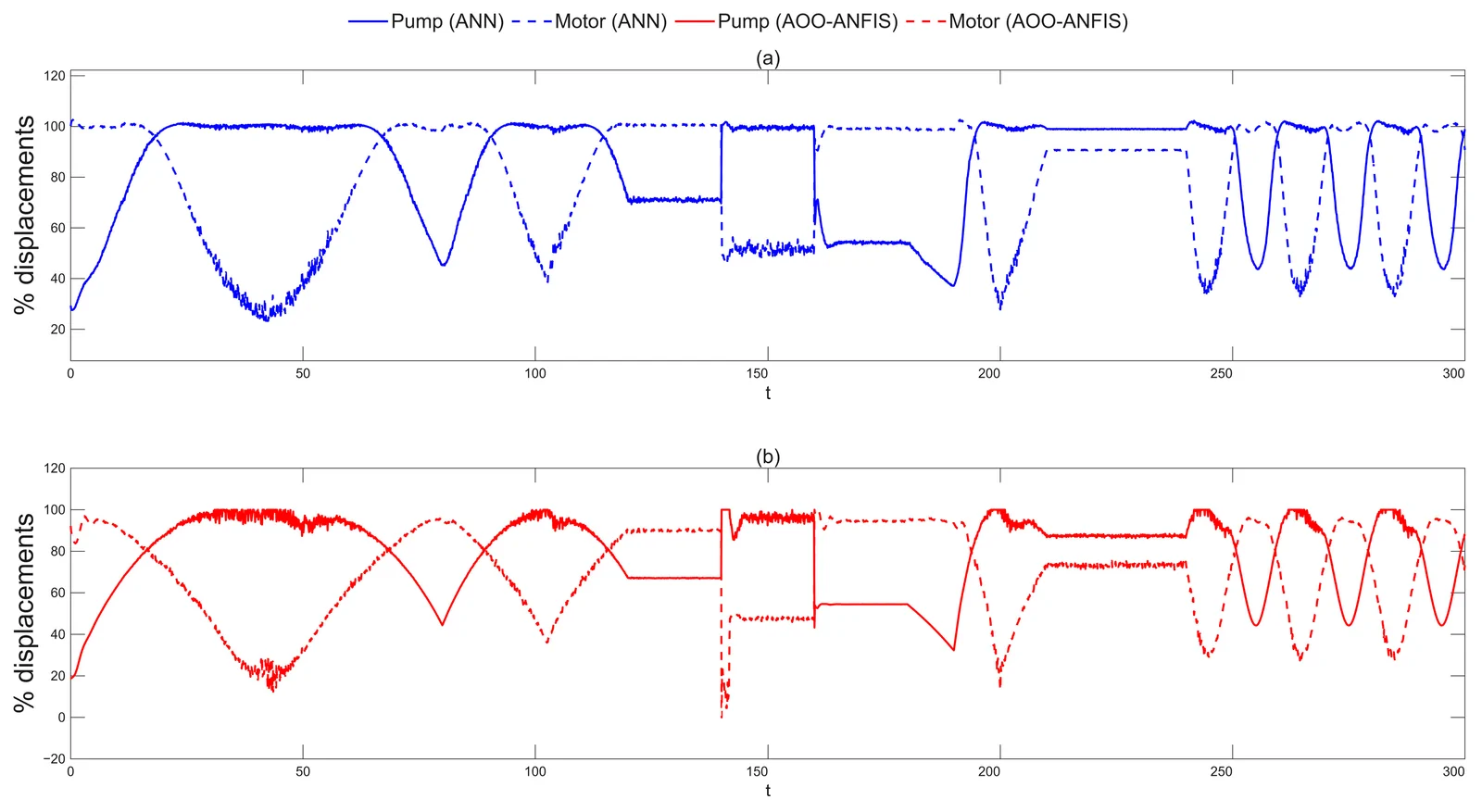
Normalized percentage of pump and motor displacements of the closed-loop systems with (**a**) ANN and (**b**) AOO-ANFIS in the case of stochastic torque disturbances for extended with degradation scenario.

**Figure 40:**
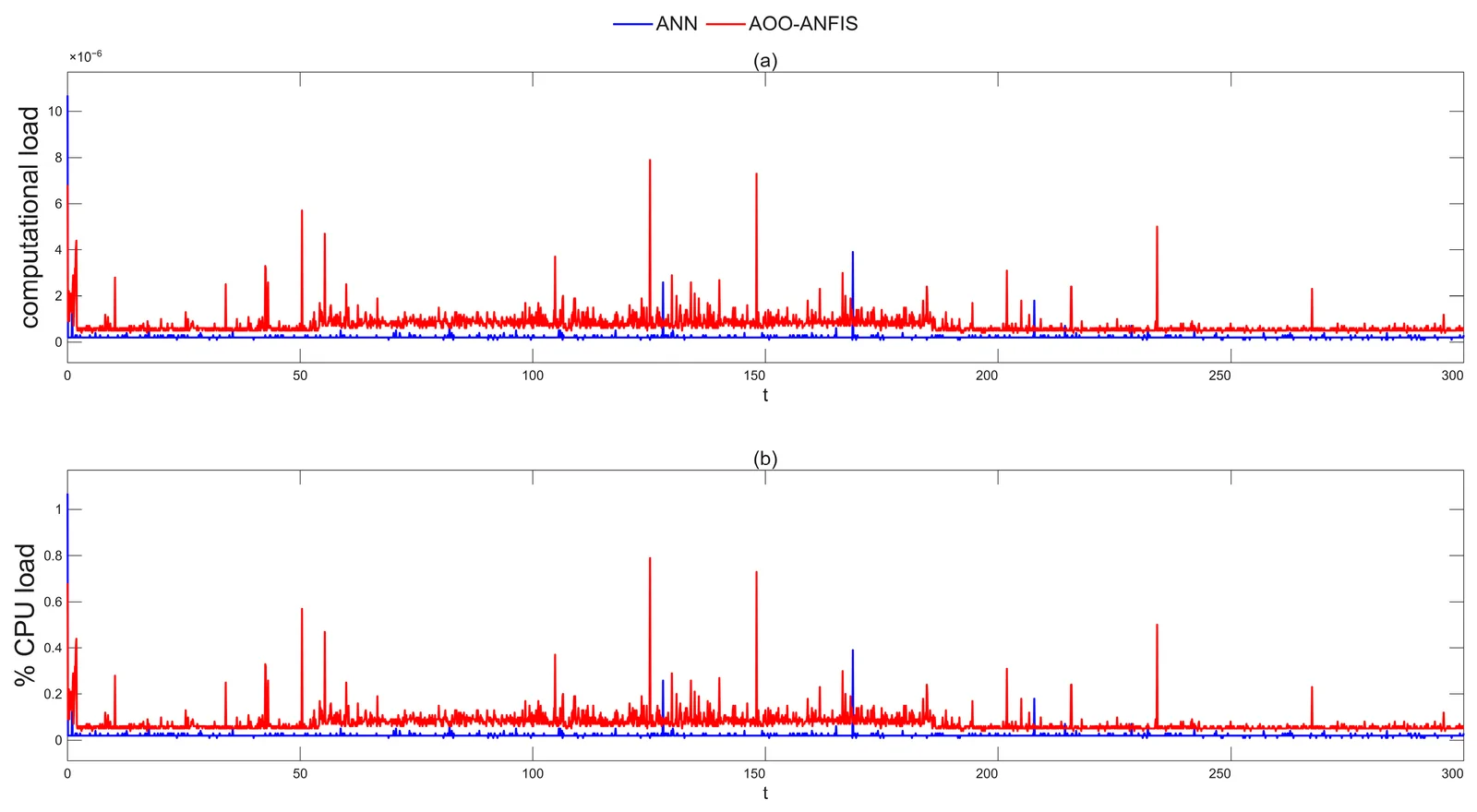
(**a**) Computational and (**b**) percentage CPU loads in the case of stochastic torque disturbances for extended with degradation scenario.

**Table 2:** ANFIS tuning performance over 10 independent runs (N=30, T=100).

Algorithm	Mean $J(x^{*})$	Standard Deviation	Best $J(x^{*})$	Test RMSE $D_{p}$	Test RMSE $D_{m}$
AOO	$3.6469\times 10^{-2}$	$2.1501\times 10^{-3}$	$3.5042\times 10^{-2}$	11.29	10.45
HHO	$6.4429\times 10^{-2}$	$2.3914\times 10^{-2}$	$3.9632\times 10^{-2}$	12.03	10.74
PSO	3.6820 × 10^−2^	2.2970 × 10^−3^	3.4620 × 10^−2^	11.40	10.23
GA	3.9020 × 10^−2^	2.3720 × 10^−3^	3.5840 × 10^−2^	11.66	10.26
CMA-ES	3.9860 × 10^−2^	8.940 × 10^−4^	3.8280 × 10^−2^	12.35	10.44

**Table 7:** MAE and $R^{2}$ values for the best run of each of the five population-based optimizers.

Algorithm	MAE $D_{p}$	MAE $D_{m}$	$R^{2}\;D_{p}$	$R^{2}\;D_{m}$
AOO	7.77	7.34	0.855	0.822
HHO	8.12	7.71	0.835	0.812
PSO	7.60	7.13	0.852	0.829
GA	7.76	7.25	0.845	0.829
CMA-ES	8.44	7.65	0.826	0.822

**Table 8:** Transient state performance measures of the PIL simulations.

Controller	Disturbance	MO (%)	$t_{p}$ (s)
MMPC	Deterministic	25	1
ANN	Deterministic	10	1
AOO-ANFIS	Deterministic	14	0.9
MMPC	Stochastic	24	1
ANN	Stochastic	10	2.8
AOO-ANFIS	Stochastic	14	1.8

**Table 9:** Steady state performance measures of the PIL simulations.

Controller	Disturbance	RMSE	IAE	ISE	ITAE	ITSE
MMPC	Deterministic	0.0485	2.80	0.424	290.3	59.2
ANN	Deterministic	0.0513	4.27	0.474	406.6	62.8
AOO-ANFIS	Deterministic	0.0447	3.44	0.360	312.5	46.5
MMPC	Stochastic	0.0694	6.22	0.869	527.4	85.3
ANN	Stochastic	0.0627	5.94	0.709	516.6	77.3
AOO-ANFIS	Stochastic	0.0485	4.09	0.424	349.4	50.2

**Table 10:** Controller performances during the PIL simulations.

Controller	Disturbance	AE (%)	ACL (s)
MMPC	Deterministic	79	4.83 × 10^−6^
ANN	Deterministic	77	2.08 × 10^−7^
AOO-ANFIS	Deterministic	69	1.18 × 10^−6^
MMPC	Stochastic	79	5.7 × 10^−6^
ANN	Stochastic	77	2.14 × 10^−7^
AOO-ANFIS	Stochastic	69	9.03 × 10^−7^

**Table 11:** Steady state performance measures of the PIL simulations for extended scenario.

Controller	Disturbance	RMSE 180–300 s	RMSE 0–300 s	Max. Abs. Error 180–300 s	IAE 180–300 s
ANN	Deterministic	0.0557	0.0531	0.17	4.85
AOO-ANFIS	Deterministic	0.0345	0.0409	0.14	3.01
ANN	Stochastic	0.0616	0.0623	0.22	5.24
AOO-ANFIS	Stochastic	0.0406	0.0456	0.26	3.52

**Table 12:** Controller performances during the PIL simulations for extended scenario.

Controller	Disturbance	AE (%)	ACL (s)
ANN	Deterministic	79	2.18 × 10^−7^
AOO-ANFIS	Deterministic	70	4.78 × 10^−7^
ANN	Stochastic	79	2.32 × 10^−7^
AOO-ANFIS	Stochastic	70	5.65 × 10^−7^

**Table 13:** Transient state performance measures of the PIL simulations for extended with degradation scenario.

Controller	Disturbance	MO (%)	$t_{p}$ (s)
ANN	Deterministic	10	3.2
AOO-ANFIS	Deterministic	16	2.1
ANN	Stochastic	10	3
AOO-ANFIS	Stochastic	16	2.2

**Table 14:** Steady state performance measures of the PIL simulations for extended with degradation scenario.

Controller	Disturbance	RMSE 0–180 s	RMSE 180–300 s	RMSE 0–300 s	Max. Abs. Error 180–300 s	IAE 180–300 s
ANN	Deterministic	0.0562	0.0656	0.0602	0.21	5.59
AOO-ANFIS	Deterministic	0.0565	0.0433	0.0516	0.20	3.54
ANN	Stochastic	0.0669	0.0707	0.0685	0.26	5.96
AOO-ANFIS	Stochastic	0.0678	0.0485	0.0608	0.34	4.04

**Table 15:** Controller performances during the PIL simulations for extended with degradation scenario.

Controller	Disturbance	AE (%)	ACL (s)
ANN	Deterministic	79	2.22 × 10^−7^
AOO-ANFIS	Deterministic	70	6.32 × 10^−7^
ANN	Stochastic	79	2.15 × 10^−7^
AOO-ANFIS	Stochastic	70	7.27 × 10^−7^

## Data Availability

The data presented in this study are available on request from the author.

## Appendix A

Table A1 lists the plant, simulation, controller and optimizer settings used in this study.

**Table A1:** Settings used in the closed-loop PIL comparison and in the ANFIS tuning.

Item	Value
Plant model	Nonlinear time-varying HST, Carlsson [8], parameters as in [18,19]
Simulation environment	MATLAB/Simulink R2024b
Solver	ode23tb (stiff; trapezoidal rule + backward differentiation formula), as in [18]
Controller sample time	Ts = 0.001 s (1 kHz)
PIL target/link	BeagleBone Black Rev C (AM3358 ARM Cortex-A8, 1 GHz, 512 MB DDR3), USB serial link to host PC, as in [18]
Simulation length	180 s (validation scenario of [18]); 300 s (extended scenario, Section 3.3)
Data logging	0.1 s interval, 1801/3001 samples, for the error metrics of Table 9, Table 11 and Table 14; AE and ACL from the PIL run itself
MMPC	2 MPCs on discrete-time linear models (primary/secondary regulation operating points), switching by sign of normalized $D_{p}$ − $D_{m};$ prediction horizon = 10, control horizon = 2; input weights = [10^−3^, 1] (primary), [1, 10^−4^] (secondary); output weights = 10^5^ (primary), 10^4^ (secondary); manipulated-variable rate weights 1; constraints $D_{p}\in \left[0,147\right],$ $D_{m}\in \left[68,160\right]$ cm^3^/rev
AOO-ANFIS structure	2 inputs, 2 outputs, 3 Gaussian MFs per input, 9 first-order Sugeno rules, 66 parameters
Input/output normalization	min–max to [−1, 1]; $D_{p}\in \left[0,147\right],$ $D_{m}\in \left[68,160\right]$ (Equation (40))
Dataset	1801 MMPC samples; 70/15/15% train/validation/test split, fixed random seed
Fitness	validation MSE averaged over both outputs (Equation (39))
AOO	*N* = 30, *T* = 100, β = 1.5, g = 9.8
HHO	*N* = 30, *T* = 100, β = 1.5 (Lévy)
PSO	swarm 30, 100 iterations, c_1_ = c_2_ = 1.49, inertia ∈ [0.1, 1.1]
GA	population 30, 100 generations, crossover fraction 0.8, elite 2, tournament selection (size 4)
CMA-ES	λ = 30, μ = 15, 100 generations, σ^0^ = 0.3 × mean range
Independent runs	10 per optimizer (PSO/GA/CMA-ES: seeds 1–10)
Disturbances	deterministic: Figure 7; stochastic: uniform noise at 1 kHz, $T_{d}\in \left[2000,2300\right]$ Nm, $T_{L}\in \left[800,1000\right]$ Nm; extended scenario: Section 3.3
Efficiency degradation test	outputs of both volumetric-efficiency maps reduced by 15%, both surrogates (AOO-ANFIS, ANN), 300 s scenario
